# Self-Assembly of Alkynylplatinum(II) Complexes for Sialic Acid Detection and Differentiation of Cancer Cells from Normal Cells

**DOI:** 10.1021/jacs.5c03210

**Published:** 2025-06-12

**Authors:** Jungu Guo, Eric Ka-Ho Wong, Guang-Xi Xu, Angela Sin-Yee Law, Michael Ho-Yeung Chan, Jonathan Lam, Ziyong Chen, Kenneth Kam-Wing Lo, Vivian Wing-Wah Yam

**Affiliations:** a Institute of Molecular Functional Materials and Department of Chemistry, 25809 The University of Hong Kong, Pokfulam Road, Hong Kong 999077, People’s Republic of China; b Chemistry and Chemical Engineering of Guangdong Provincial Laboratory, No. 1, College Road, Tuojiang Street, Jinping District, Shantou, Guangdong 515000, People’s Republic of China; c Department of Chemistry, 53025 City University of Hong Kong, Tat Chee Avenue, Kowloon, Hong Kong 999077, People’s Republic of China

## Abstract

A series of platinum­(II) complexes incorporating a histidine moiety and/or positively charged group has been designed and synthesized to explore the specific binding between platinum­(II) complexes and the analyte and their sensing ability. The specific hydrogen bonding between the histidine moiety and sialic acids, and the electrostatic interaction between the positively charged trimethylammonium group of the platinum­(II) complexes and negatively charged carboxylate groups of sialic acids have been found to enhance the binding affinity. The supramolecular assembly of platinum­(II) complexes upon binding to sialic acids induces remarkable luminescence changes due to noncovalent Pt­(II)**···**Pt­(II) and π–π stacking interactions, achieving sensing and visualization of sialic acids. A novel luminescence assay has been developed to differentiate cancer cells from normal cells based on the supramolecular assembly of platinum­(II) complexes upon binding to a high level of sialic acids due to its excellent imaging ability toward sialic acids in live cells. The complexes have been adopted to visualize the variation of sialic acids in live cells after treatment with enzymes to remove sialic acids, paving the way for screening of novel anticancer agents. The present sensing strategy for differentiating cancer cells from normal cells facilitates early diagnosis and therapeutic guidance.

## Graphical abstract

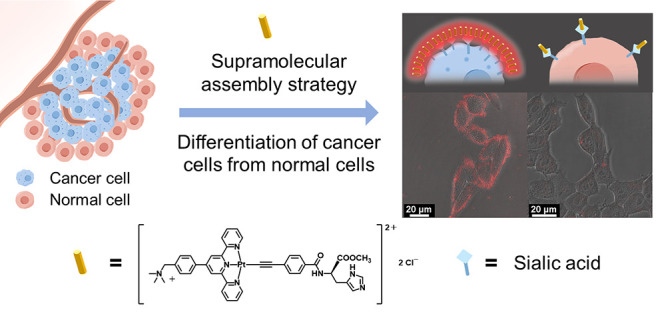

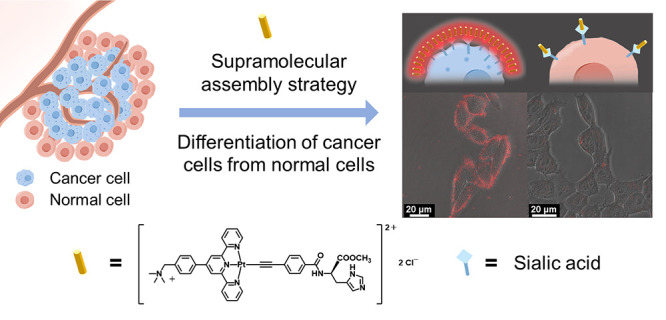

## Introduction

All eukaryotic cells are covered with a dense and complex layer of glycans, which play an essential role in cell trafficking and signaling in the form of glycoproteins and glycolipids.^1–3^ Sharing a nine-carbon backbone, sialic acids are typically located at the terminal ends of the glycan chain on the cellular surfaces.^4,5^ Sialic acids modulate various biological processes, contributing to signal transduction and the immune response. To evade recognition from the immune system and facilitate the metastatic spread, the increased level of sialic acids on the surface of cancer cells is used as a biological mask.^6–10^ Thus, the overexpressed sialic acids can be utilized as diagnostic biomarkers for cancer.

Common methods to quantify sialic acids include high-performance liquid chromatography (HPLC), mass spectrometry (MS), and colorimetric assay.^11–14^ These assays are unable to provide dynamic information *in vivo*, thereby limiting their application in diagnosing cancer. An efficient approach for real-time and *in vivo* visualization of sialic acids to facilitate diagnosis and therapeutic guidance is highly desirable. The recent sensing strategies consist of bioaffinity, chemical, metabolic, and linkage labeling, but most of them would suffer from high cost, low selectivity, and challenging synthesis.^2,11–22^ In order to improve the selectivity, a biocompatible histidine moiety has been introduced for recognition of sialic acids *via* the antibody screening experiment.^23,24^ However, the rather small influence exerted on the electronic properties of the histidine moiety after binding with sialic acids resulted in negligible luminescence signal changes, greatly hindering its application through incorporation to commercially available fluorescent dyes.

Low-energy red to near-infrared (NIR) luminescent platinum­(II) complexes have attracted much attention because of their self-assembly ability associated with their square-planar geometry driven by Pt­(II)**···**Pt­(II) and π–π stacking interactions.^25–35^ Triggered by the addition of biological analytes or variation of microenvironment, the supramolecular assembly behaviors of platinum­(II) complexes have led to remarkable enhancement of low-energy red to NIR emission, which can be utilized to visualize biological species *in vivo* and achieve organelle imaging.^36^ Several series of alkynylplatinum(II) complexes have been reported to detect and/or visualize various biological species, such as single-stranded DNA,^37^ G-quadruplex,^38^ monosaccharides,^39^ aptamers,^40^ human serum albumin (HSA),^41^ heparin,^42^ trypsin,^42^ adenosine triphosphate (ATP),^43^ acetylcholinesterase,^44^ amyloid protein,^45^ and RNA.^46^ In sharp contrast to small-molecule organic fluorophores, platinum­(II) complexes exhibit large Stokes shift contributing to low-energy emission in the red to NIR region as well as enhanced photostability and excited-state lifetime in the microsecond range.^47^ Such low-energy emissions can achieve deeper penetration depth, minimize background autofluorescence, facilitate time-resolved gated detection, and achieve higher signal-to-background ratio.^48^ These excellent features favor the application of platinum­(II) complexes in live-cell and tissue imaging.

Incorporation of histidine into platinum­(II) complexes would endow the complexes with a specific binding affinity to sialic acids (Scheme S1). It is envisaged that the abundance of sialic acids on the surface of cancer cells could bring the platinum­(II) complexes into close proximity, leading to the supramolecular self-assembly of the platinum­(II) complex with luminescence turn-on in the red to NIR region. More interestingly, only very few sialic acids on the surface of normal cells are known to be capable of binding to the histidine group that is attached to the platinum­(II) complexes, disfavoring supramolecular assembly of the platinum­(II) complexes. Thus, the supramolecular self-assembly behavior of platinum­(II) complexes may provide a novel and efficient detection strategy for different levels of sialic acids. Common probes, including indocyanine green (ICG) and 5-aminolevulinic acid (5-ALA), have been used for cancer cell differentiation in clinical use. The cancer cells are identified by the difference in intensity of the fluorescence signals, which suffers from strong background signals that interferes with detection accuracy. Since normal cells will not be stained by platinum­(II) complexes, the proposed method can accurately detect cancer cells with a low false-positive rate in comparison to the current commercial probes. Such distinct responsive behaviors toward varying levels of sialic acids can minimize the possibility of false-positive results commonly encountered in traditional turn-on sensors. In order to investigate the sensing ability of platinum­(II) complexes in aqueous solution, *N*-acetylneuraminic acid (Neu5Ac) and polysialic acid (polySia) have been chosen as *in vitro* models for normal and cancer cells, respectively (Scheme 1).

**Scheme 1:**
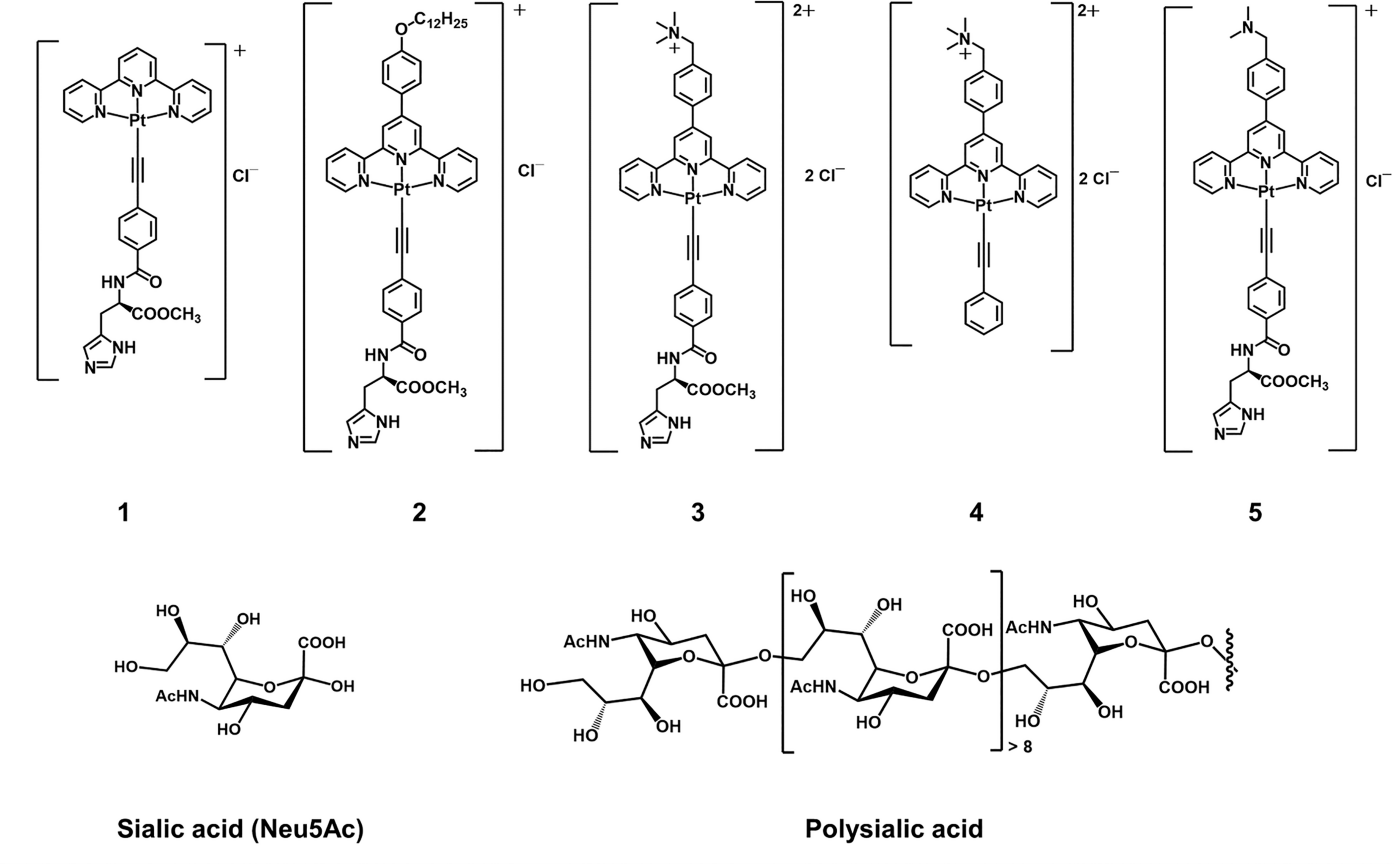
Chemical Structures of **1**–**5**, Neu5Ac, and Polysialic Acid (polySia)

In the present study, alkynylplatinum­(II) terpyridine complexes incorporated with a histidine group are designed and characterized for specific binding interaction with sialic acids (Scheme 1, Schemes S2 and S3, and Figures S1–S16). Subtle changes in the pincer ligands would be made to explore the effects of amphiphilicity on the sensing capability of the platinum­(II) complexes and compared them to a control complex without the histidine moiety.

## Results and Discussion

The photophysical properties of **1**–**5** have been studied in Tris-HCl buffer (10 mM Tris, 10 mM NaCl, pH = 8.0). The UV–vis spectra of **1–5** exhibit high-energy absorption bands at 260**–**290 nm and low-energy bands at 440–470 nm (Figures S17a–S21a). The high-energy absorption bands are ascribed to intraligand (π → π*) transitions of terpyridine and alkynyl ligands,^49–51^ while the lower-energy absorption bands are assigned as an admixture of metal-to-ligand charge transfer (MLCT) [dπ­(Pt) → π*­(tpy)] and ligand-to-ligand charge transfer (LLCT) [π­(C≡C) → π*­(tpy)] transitions.^49–51^

Upon gradual addition of polySia ([sialic acid] = 0–60 μM) to an aqueous buffer solution of **3**, a decline and slight red shift of the low-energy absorption band at 445 nm and the apparent growth of the lower-energy absorption shoulder at 600 nm are observed with an isosbestic point at 530 nm (Figure S19a). The occurrence of the absorption shoulder can be ascribed to the metal–metal-to-ligand charge transfer (MMLCT) transition. This demonstrates that the addition of polySia facilitates the close proximity of complex molecules of **3**, leading to the formation of metal–metal interactions that lead to the MMLCT bands. Further addition of polySia ([sialic acid] = 60–114 μM) makes no significant difference to the absorption band of **3**, suggesting that the binding might have reached an equilibrium (Figure S19b). The assembly of **3** induced by polySia has been revealed by emission titration experiments. The emission band at *ca*. 760 nm, which exhibits an obvious increase in intensity upon addition of polySia, is typical of the triplet metal–metal-to-ligand charge transfer (^3^MMLCT) excited state (Figure 1).^52–54^ Such a remarkable enhancement of the emission band in the NIR region suggests the formation of self-assembled platinum­(II) complexes.

**Figure 1:**
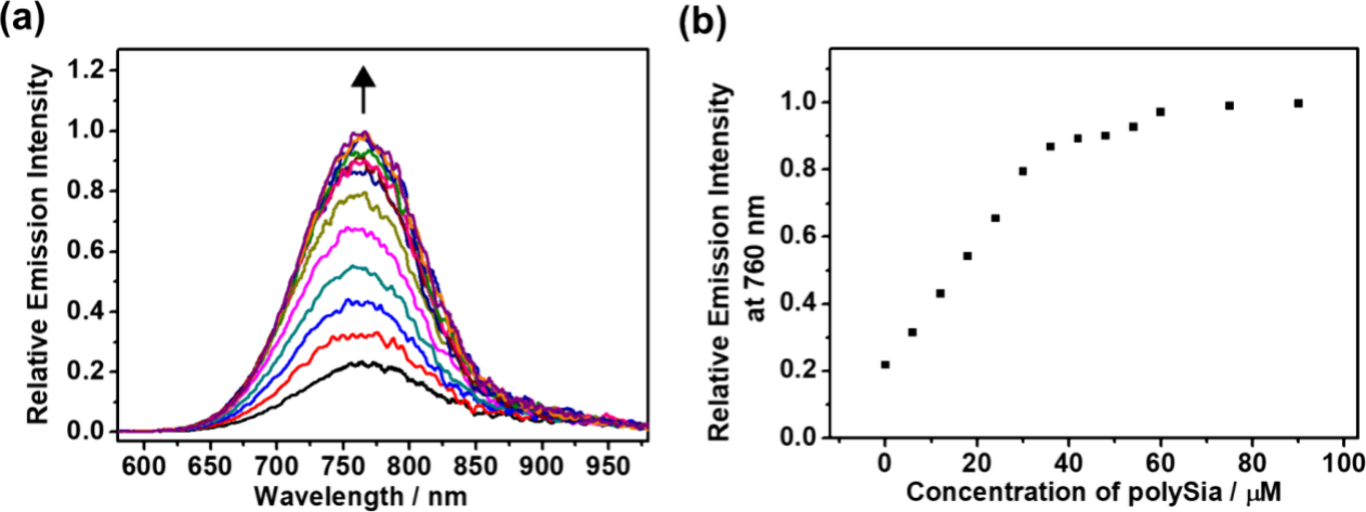
(a) Emission spectra of **3** (30 μM) upon the addition of polySia ([sialic acid] = 0–90 μM) in Tris-HCl buffer (10 mM Tris, 10 mM NaCl, pH = 8.0). Excitation wavelength was at 530 nm. (b) The relative emission intensity at 760 nm versus concentration of polySia in a range of 0–90 μM.

UV–Vis and emission titration experiments have also been carried out to investigate the sensing behaviors of **1** (Figure S17), **2** (Figure S18), **4** (Figure S20), and **5** (Figure S21). Compared to the UV–vis spectral changes of **3** upon the addition of polySia, similar spectral changes can be observed for **5** due to their similar structures (Figure S21a). The emission titration spectra of **5** exhibit a less drastic increase in the intensity upon photoexcitation at the isosbestic wavelength, suggesting the important role of the trimethylammonium group of **3** (Figure S21b). Addition of polySia to a solution of **1** or **4** results in an increase in the low-energy absorption band at around 450–650 nm, accompanied by an obvious decrease of the high-energy absorption band at 280–290 nm (Figures S17a and S20a). The emission titration spectral changes of **1** and **4** also demonstrate enhancement of the ^3^MMLCT emission band (Figures S17b and S20b). However, the addition of polySia to an aqueous solution of **2** shows insignificant changes to the absorption band (Figure S18a). The emission titration spectra exhibit a slight enhancement of the ^3^MMLCT emission band (Figure S18b). The titration spectra of the platinum­(II) complexes suggest the formation of supramolecular assemblies after the addition of polySia.

In view of the drastic spectroscopic changes of the platinum­(II) complexes upon addition of polySia, transmission electron microscopy (TEM) has also been conducted to visualize the formation of supramolecular aggregates (Figures S22–S26), which can help to explain the effect of different functional recognition moieties on their self-assembly and sensing properties. Before addition of polySia, the TEM image of **2** shows nanoaggregates (Figure S23a), indicating that the long alkyl chain induces nanostructure formation and facilitates the self-assembly properties.^55^ The low-energy absorption tail at *ca*. 600 nm and emission band at 728 nm suggest the formation of self-assemblies of **2** (Figure S18). The presence of ground-state aggregates of **2** might hinder the binding to polySia, which corroborates with the insignificant photophysical and morphological changes of **2** after the addition of polySia (Figure S23b). The TEM images of **1** and **5** before the addition of polySia show polydisperse nanostructures, suggesting the formation of supramolecular assemblies (Figures S22a and S26a). No obvious nanoaggregates can be observed from the TEM images of **3** and **4** (Figures S24a and S25a), suggesting that the platinum­(II) complexes incorporating a positively charged trimethylammonium group are well-dispersed in aqueous solution. These results corroborate with the UV–vis and emission studies of the platinum­(II) complexes. The smaller extent of self-assembly of **3** and **4** might be attributed to the aryl groups containing the more bulky positively charged trimethylammonium group, which would introduce steric hindrance and also stronger charge repulsion between the dicationic complexes.^54^ The trimethylammonium group can suppress the self-assembly of the complexes, facilitating significant spectral changes of **3** and **4** toward polySia.

After the addition of polySia, TEM images of **1**, **3**, **4**, and **5** show nanofibrous structures with widths of about 20–60 nm for **1**, **3**, and **5** and 200 nm for **4** (Figures S22b,c, S24b,c, S25b, and S26b,c). Results from dynamic light scattering (DLS) studies also exhibit a gradual increase in an average hydrodynamic diameter of **3** from 410 to 868 nm upon the addition of polySia (Figure S27, Table S1), confirming the formation of supramolecular assemblies. Scanning transmission electron microscopy-energy-disperse X-ray spectroscopy (STEM-EDX) has been used to conduct the elemental analysis to confirm the presence of platinum­(II) complexes. The signals at 2.04 and 9.45 keV are typical of platinum (M_ω_, L_α_) in the nanoaggregates formed from platinum­(II) complexes (Figures S22d, S23c, S24d, S25c, and S26d). A strong emission intensity at 650–750 nm, characteristic of ^3^MMLCT emission, can be observed in the confocal images (Figures S28–S30), further confirming that the nanoaggregates originate from supramolecular assembly of platinum­(II) complexes, aided by formation of the platinum complex−polySia ensemble. In addition, the SAED patterns of **1**, **3**, and **4** upon addition of 1 equiv of polySia display rings or arcs with *d*-spacings of 3.2–3.5 Å (Figure 2), which is suggestive of the involvement of Pt­(II)**···**Pt­(II) and/or π–π stacking interactions in the supramolecular assemblies (Table S2).

**Figure 2:**
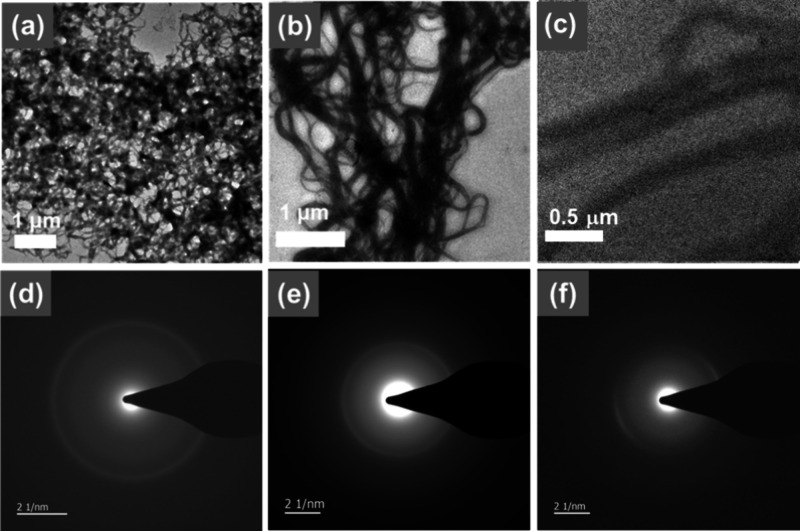
(a–c) TEM images and (d–f) SAED patterns of the nanoaggregates formed from (a, d) **1**, (b, e) **3**, and (c, f) **4** (30 μM) upon addition of polySia (30 μM) in the H_2_O.

The apparent binding constants (log *K*) and dissociation constant (*K*_d_) of the platinum­(II) complexes toward polySia have been determined by the Hill equation to compare their sensing abilities (Table 1), with the exception of **2** due to insignificant spectral changes. The values of the Hill coefficients of **3**–**5** are larger than 1, indicative of positive cooperative binding modes. On the other hand, the value of Hill coefficient of **1** is smaller than 1, which implies a negative cooperative binding mode. The trend of the dissociation constants *K*_d_ shows that the sensing ability toward polySia follows an order of **3** > **5** > **4** > **1**, and such differences are associated with the different functional groups in the molecular structure.

**Table 1:** Binding Constants (log *K*), Dissociation Constant (*K*_d_), and Hill Coefficients (*n*)­^a^ of the Complexes Determined by Hill Plots in Tris-HCl Buffer (10 mM Tris, 10 mM NaCl, pH = 8.0)

sample^a^	log *K*	*K*_d_ (M)	*n*
**1**	4.45	3.55 × 10^–5^	0.83
**3**	8.02	9.52 × 10^–9^	1.75
**4**	6.30	5.01 × 10^–7^	1.46
**5**	6.85	1.40 × 10^–7^	1.47

a The binding constant of **2** cannot be determined.

Given that both the positively charged trimethylammonium group and the histidine moiety in the platinum­(II) complexes could potentially bind to polySia *via* electrostatic interaction and specific hydrogen bonds, respectively, to bring the complexes into close proximity and lead to a switch-on of ^3^MMLCT emission bands, density functional theory (DFT) calculations have been conducted to investigate the binding modes of **3** and the sialic acid. The noncovalent interaction (NCI) analysis has been conducted with a color gradient corresponding to the interaction strength. Strong N–H**···**O and O–H**···**N hydrogen bonds between the hydroxy group of the sialic acid and the imidazole moiety of **3** are found, appearing as blue circles on the interaction surface (Figure S31). The protonation and deprotonation of pH-sensitive moieties on platinum­(II) complexes could be monitored by UV–vis absorption spectroscopy. Since the supramolecular assembly behavior of these complexes would be modulated by pH, a plot of the absorbance of low-energy MMLCT band of the complexes against pH could be employed to determine the p*K*_a_ value of the imidazolium moiety.^54^ The p*K*_a_ value of the imidazolium moiety is found to be around 5.5 (Figure S32). A less significant change in ^3^MMLCT emission of **3** in aqueous buffer (pH 6.2 and 5.5), along with a decrease in binding constants (log *K*) of **3** toward polySia with decreasing pH from 8.0 to 5.5, is observed (Figure S33, Table S3). These suggest that protonation of the imidazole moiety at lower pH levels could affect the binding of the complex to sialic acid, which could be related to the disruption of the O–H**···**N hydrogen bond, as further supported by NCI analysis on a system comprising protonation form of **3** (the imidazolium moiety) and sialic acid (Figure S34). These results explain the specific binding ability of the histidine moiety in **3** toward sialic acid. On the other hand, it has been reported that the p*K*_a_ of a positively charged −CH_2_NHMe_2_^+^ group on the terpyridine ligand of platinum­(II) complexes is 8.53.^54^ By comparing the binding constants of **5** toward polySia in Tris-HCl buffer (10 mM Tris, 10 mM NaCl) with varying pH, the effect of positive charge on binding affinity toward polySia can be investigated. Interestingly, a decrease in binding constants (log *K*) of **5** is observed with increasing pH from 8.0 to 9.0 (Figure S35, Table S4), indicating the important role of the positive charge in binding toward polySia. The imidazole form should exist as the predominant species in the platinum­(II) complexes at pH values from 8.0 to 9.0, indicating that the possible existence of an extra positive charge arising from the protonation of imidazole to give the imidazolium moiety is minimal, with insignificant interference toward the sensing capability of **3**. The zeta potential measurements for **3** have been conducted in aqueous solution to study the involvement of the electrostatic interaction in the binding. With the increasing concentration of polySia, the zeta potential gradually drops from +23 to −30 mV (Figure S36), in line with the electrostatic interaction between **3** and polySia, which alters the overall charge from positive to negative. The electrostatic potential (ESP) surface has also been computed to confirm the presence of the electrostatic interaction in the ensemble (Figure S37). The most electron-rich area on the ESP surface of sialic acid is found at the negatively charged carboxylate group, while the electron-deficient region on the ESP surface of **3** is located at the positively charged trimethylammonium and phenyl groups. The high electrostatic potential difference is neutralized in the ensemble consisting of **3** and sialic acid, which suggests the significant contribution of electrostatic interaction in the binding ability of the platinum­(II) complexes. The participation of polySia in the binding interaction is present even in their ^1^H NMR spectra in DMSO-*d*_6_ with a similar and very low level of water, where the disappearance of signal at δ 12.93 ppm, assignable to the proton from −COOH is observed (Figure S38). Further analyses on the optimized geometries and the calculated binding energies of ensembles comprising platinum­(II) complexes and sialic acid have been carried out (Figure 3 and Table S5). Notably, the Δ*E*_bind_ values for (**3**(His)-sialic acid), (**3**(NMe_3_)-sialic acid), and (**5**(NMe_2_)-sialic acid) are calculated to be −6.0, −9.3, and −4.9 kcal mol^–1^, respectively. **3** is therefore shown to have the strongest binding affinity, with both moieties interacting more strongly to sialic acid than that of **5**, in agreement with results from spectroscopic studies.

**Figure 3:**
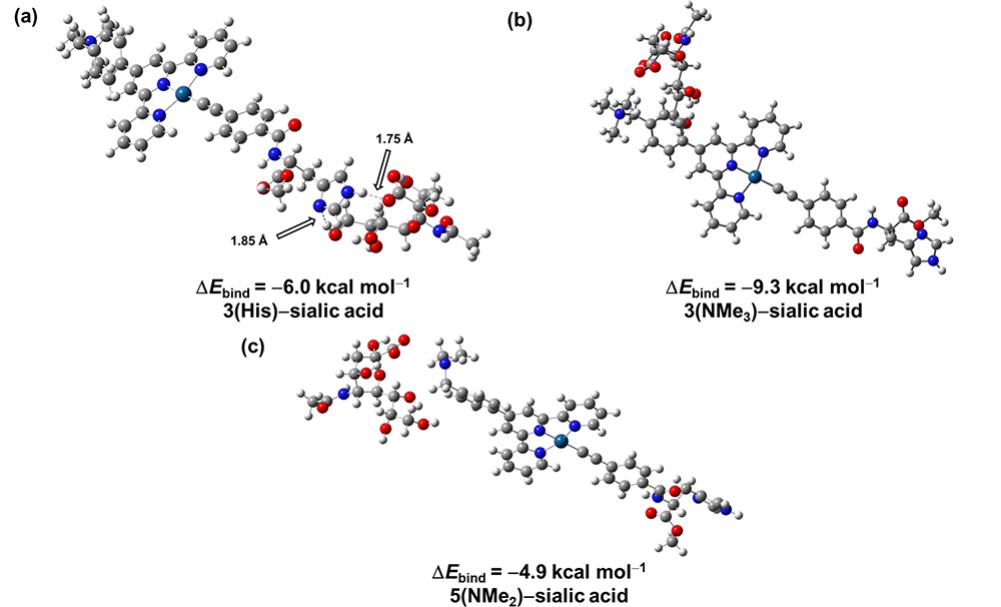
Optimized geometries and the corresponding binding energy (kcal mol^–1^) of the ensemble of (a) **3**(His)-sialic acid, (b) **3**(NMe_3_)-sialic acid, and (c) **5**(NMe_2_)-sialic acid.

It is found that **3** with both functional groups exhibits the most obvious emission enhancement and the highest binding constants to polySia due to the synergistic effect of the histidine and trimethylammonium group. Thus, **3** can be represented as the most optimal sensor for polySia. The titration experiments of **3** upon the addition of Neu5Ac (0–90 μM) have been carried out to investigate the responsive behaviors toward sialic acids in the monomeric form as an *in vitro* model of normal cells in an aqueous buffer. Upon photoexcitation, a slight drop in the ^3^MMLCT emission has been observed, indicating an insignificant change in the assembly behavior of **3** in the presence of Neu5Ac (Figure 4). This corroborates with the corresponding HR-ESI mass spectrum, which exhibits the existence of [**3**+Neu5Ac–H]^+^, confirming the formation of ensemble and binding interaction between platinum­(II) complex molecules and sialic acids (Figure S39). ^1^H–^1^H Nuclear Overhauser effect spectroscopy (NOESY) NMR experiments have further been conducted in the solution of **3** to investigate the intermolecular interactions upon addition of polySia and Neu5Ac (Figures S40–S44). New NOE signals between terpyridine protons (H_e_ and H_g_, H_e_ and H_c_, as well as H_f_ and H_d_), which were absent in a solution of **3** and Neu5Ac, are observed in the NOESY NMR spectrum of **3** and polySia, suggesting the formation of self-assemblies of **3** after the addition of polySia. The completely distinct spectroscopic behaviors toward Neu5Ac and polySia suggest that **3** is a promising candidate in differentiation of cancer cells from normal cells, which can avoid false-positive results in bioimaging.

**Figure 4:**
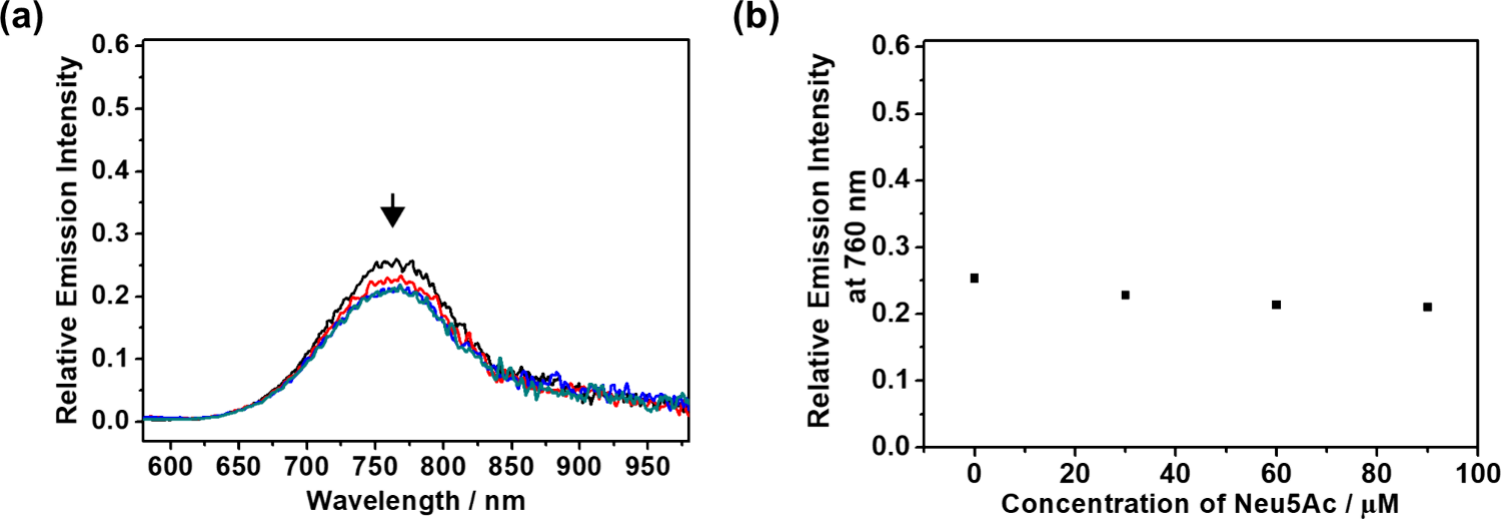
(a) Emission spectra of **3** (30 μM) upon the addition of Neu5Ac (0–90 μM) in Tris-HCl buffer (10 mM Tris, 10 mM NaCl, pH = 8.0). (b) Relative emission intensity at 760 nm versus concentration of Neu5Ac in a range of 0–90 μM.

The influence of different monosaccharides (1, 5, and 10 equiv of mannose, glucose, lactose, sucrose, galactose) on the emission spectra of **3** has been studied (Figure S45). **3** is found to show no obvious spectroscopic change toward other monosaccharides, demonstrating that the sensing ability of **3** toward polySia would not be interfered by other monosaccharides. Such a high selectivity would facilitate further applications in cell imaging experiments. Given the intracellular and extracellular pH range of normal cells of around 7.2 and 7.4 ^56,57^ as well as the mildly acidic extracellular environment of tumor cells at pH 6.8–7.0,^58^ the effect of pH on the sensing behavior of **3** toward polySia has also been examined. The UV–vis and emission spectra in different aqueous buffers (pH = 6.8, 7.2, 7.4 and 9.0) show insignificant changes compared to that acquired in the aqueous buffer (pH = 8.0) (Figures S46–S48), which are consistent with the findings that the imidazole moiety is the predominant species (percentage of platinum­(II) complexes in the system obtained from the Henderson–Hasselbach equation that contains the charge-neutral imidazole moiety are 98.8% at pH 7.4, 96.9% at pH 7.0, and 95.2% at pH 6.8). The analysis indicates that the physiological and pathological pH range (pH = 6.8–7.4) poses insignificant interference toward the sensing capability of **3**, as further supported by the minimal difference in emission spectra of **3** at different pH values (Figure S49). These confirm its promising bioimaging application. The limit of detection (LOD) of **3** for polySia has been determined, in which a low LOD value of 73.78 nM is found (Figure S50).

As a result of its distinct responsive behaviors toward Neu5Ac and polySia as well as its high sensing selectivity toward polySia even under cellular conditions, biological application of **3** in the differentiation of cancer cells from normal cells has been studied. Other platinum­(II) complexes have also been used in cell imaging experiments for comparison. Cell imaging assays have been performed in two cancer cell lines, HepG2 cells, HeLa cells, and a normal cell line, HEK293T cells. A HeLa cell exhibits approximately 4.6 × 10^7^ sialic acid molecules,^59^ while a HepG2 cell exhibits around 5.4 × 10^9^ sialic acid molecules.^60^ Although the exact sialic acid content of HEK293T cells was not reported, the obvious difference of sialic acid levels between HEK293T and HeLa cells monitored by the HPLC method exhibited low distribution of sialic acids on the surface of the normal cell line.^61^ Particularly, concentration-dependent and time-dependent cell imaging experiments have been conducted to optimize the incubation conditions (Figures S51 and S52), in which 10 μM was chosen as the working concentration, while 0.5 h was chosen as the optimal incubation time.

To investigate the ability of platinum­(II) complexes in the differentiation of cancer cells from normal cells, **1**–**5** have been applied in bioimaging of HepG2 cells and HEK293T cells. Upon incubation with **3** at 37 °C for 0.5 h, obvious luminescence signals are observed on the surface of HepG2 cells, while the absence of such signals is found on the surface of HEK293T cells from the confocal images, confirming the ability of **3** to differentiate cancer cells from normal cells (Figure 5). Similar luminescence signals are observed in **4** and **5** upon incubation, but with weaker luminescence signals observed on the surface of HepG2 cells (Figures S53 and S54). HeLa cells incubated with **3** have also been imaged, demonstrating that **3** is able to visualize the sialic acids on the surface of HeLa cells (Figure S55). In sharp contrast to complexes **3**–**5**, complexes **1** and **2** are found to induce cell death in HEK293T cells, suggesting that complexes **1** and **2** are cytotoxic toward normal cells. This may be due to the tendency of **1** and **2** to form aggregates in the aqueous solution, as revealed from the TEM images. Moreover, no luminescence signal is observed in the confocal images of HepG2 cells (Figure S56). These show that **3**–**5** can differentiate cancer cells from normal cells, and **3** is the most optimal bioimaging agent among them, which is in good agreement with results from spectroscopic studies.

**Figure 5:**
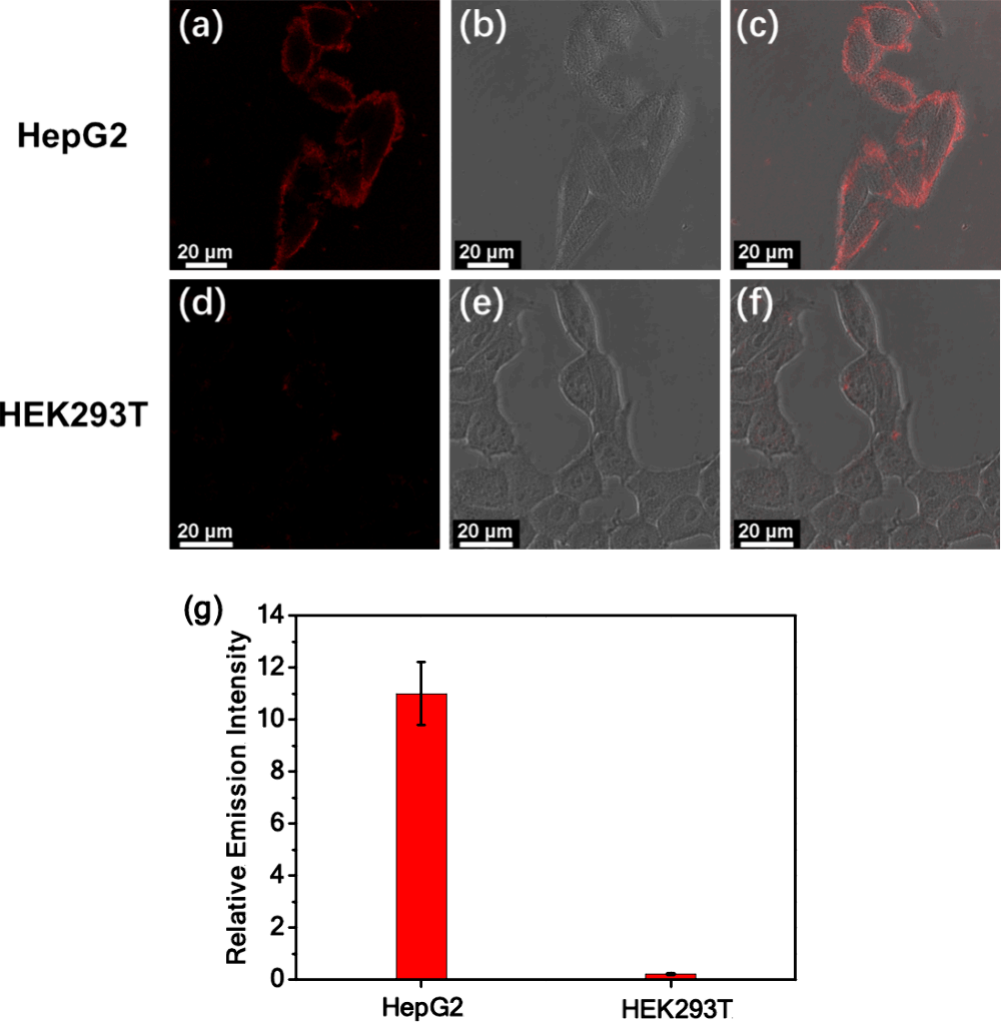
Confocal images of (a–c) live HepG2 cells and (d–f) live HEK293T cells stained with **3** (10 μM) at 37 °C for 30 min. (a, d) Luminescence, (b, e) bright-field, and (c, f) merged confocal images with bright-field and emission collected at 700–800 nm upon photoexcitation at 450 nm. (g) A bar graph comparing the relative emission intensity of HepG2 and HEK293T cells stained with **3**.

Since sialic acids are located at the terminal end of glycans on the cellular surfaces, the colocalization assays of **3** with a commercial membrane tracker have been studied in HepG2 cells. The high degree of colocalization indicates that **3** is mostly localized on the cellular membranes (Figure S57). Further comparison of the cell imaging ability of **3** to a commercial dye for sialic acids, FITC-conjugated lectin, has been conducted in cancer cells. Similar localization of **3** and FITC-conjugated lectin on the surface of HepG2 cells is found from the confocal images (Figure 6a–d) with a high colocalization coefficient of 0.87, confirming the staining ability of **3** toward sialic acids on the cellular surface (Figure S58). Such a high degree of colocalization can be viewed by the yellow color in the merged images (Figure 6d). Upon further comparison of the sensing ability of **3** and FITC-conjugated lectin, it is found that no luminescence signals for **3** but obvious fluorescence signals for FITC-conjugated lectin can be observed on the membrane of HEK293T cells (Figure 6b,f). The difference can be explained by their distinct sensing strategies. Commercial dyes for sialic acids, usually comprising a fluorescent dye and a recognition moiety, target sialic acids on the cellular surface and exhibit fluorescence signals that is proportional to the level of sialic acids. However, this approach can lead to false-positive results in some normal cell lines with high level of sialic acids, limiting their potential clinical application. In stark contrast, the novel sensing strategy based on the presence and absence of assembly behaviors of platinum­(II) complexes under environments with high and low levels of sialic acids can differentiate cancer cells from normal cells *via* obviously different luminescence signals. This approach provides an improved solution for the accurate detection and diagnosis of cancer cells.

**Figure 6:**
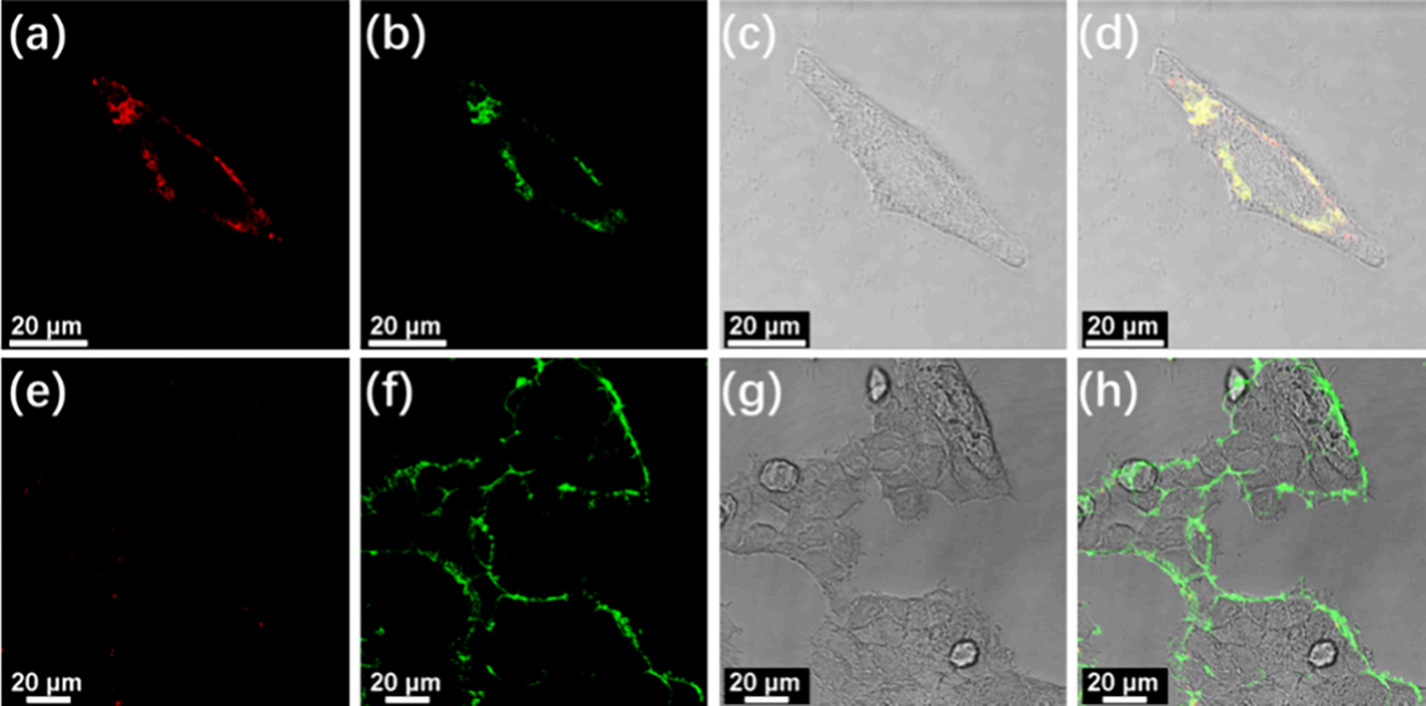
Confocal images of (a–d) live HepG2 cells and (e–h) live HEK293T cells stained with **3** (10 μM) at 37 °C for 0.5 h, followed by incubation with paraformaldehyde fix solution for 15 min and FITC-conjugated lectins (20 μg/mL) at 37 °C for 1 h. (a, e) Luminescence collected at 700–800 nm with excitation wavelength at 405 nm; (b, f) luminescence collected at 500–550 nm with excitation wavelength at 488 nm; (c) bright-field and (d) merged confocal images with emission collected at 700–800, 500–550 nm, and bright-field.

Apart from the differentiation of cancer cells from normal cells, another application of **3** in screening inhibitors for sialic acids has been evaluated. Inhibitors to remove sialic acids from the surface of cancer cells have been reported as a novel anticancer strategy, leading to an urgent need to develop a tool to screen highly effective inhibitors.^62^ Neuraminidase is one example that can decrease the level of sialic acids on the cellular surface upon incubation with HepG2 cells.^63^ The confocal images show decreasing luminescence signals in live HepG2 cells stained with **3** after treatment with neuraminidase (0.1 U/mL) (Figure S59), suggesting that the complex can visualize sialic acids on the cellular surface by the variation in the luminescence intensity. It is envisaged that **3** can contribute to novel anticancer medicine discovery and investigation on the mechanism during pathological processes, which is of great significance in anticancer therapy.

The cytotoxicity of **3** toward HepG2 cells has been conducted *via* an MTT cell viability assay to investigate the biocompatibility. Clinical probes such as ICG were reported to be administered *in vivo* 24 or 48 h before surgery for therapeutic guidance, with varying concentrations based on the specific clinical application and patient condition.^64,65^ Thus, in order to further explore the long-term effects of varying concentrations of complex **3** and to mimic the clinical settings, cells viability has been assessed after 24 and 48 h of incubation with varying concentrations of **3** (Figure S60). The results show high cell viability (over 99 and 70%, respectively) across all concentrations. Since a very low concentration of **3** is chosen in the biological experiments, together with its good biocompatibility and low cytotoxicity, it will make **3** suitable for application in biological sensing and imaging assays.

## Conclusions

A series of alkynylplatinum­(II) complexes has been designed as biological probes for sialic acid detection and bioimaging tools to differentiate cancer cells from normal cells. Upon addition of polySia, the appearance of the MMLCT absorption and ^3^MMLCT emission band of the platinum­(II) complexes as well as morphological changes suggest the formation of supramolecular ensembles. Comparison of binding constants has confirmed the importance of the histidine group and the positively charged trimethylammonium group in conferring their high specificity and affinity toward sialic acids, driven by specific hydrogen bonding and electrostatic interaction between the platinum­(II) complexes and sialic acids. The application of platinum­(II) complexes in cell imaging facilitated by the supramolecular assembly of a series of complexes *via* Pt­(II)**···**Pt­(II) and π–π stacking interactions between adjacent molecules has been studied. As compared to a commercial dye FITC-conjugated lectin, platinum­(II) complexes have been demonstrated to differentiate cancer cells from normal cells, which is beneficial in avoiding false-positive results. This novel sensing strategy has been developed by the distinct responsive behaviors, in which the assembly of platinum­(II) complexes has been observed when binding to high level of sialic acids on the surface of cancer cells to give low-energy NIR emission. This is in sharp contrast to the lack of assembly behavior toward low level of sialic acids on the surface of normal cells. Furthermore, the complex has also been utilized in visualizing the variation of sialic acids after incubating with sialic acid inhibitors. This can be developed to screen the anticancer therapeutic agents that remove sialic acids on the cellular surface. The present study illustrates the desirable properties of platinum­(II) complexes, which can be used as promising probes for sialic acid detection, screening inhibitors for sialic acids and differentiation of cancer cells from normal cells.

## Supplementary Material



## Notes

Experimental procedures, UV–vis absorption spectra, emission spectra, DLS data, zeta potential data, TEM images, STEM image, STEM-EDX, EDX spectrum, ^1^H NMR spectra, confocal images, MTT cell viability data, and computational details (PDF)

## References

[1] Dube D. H., Bertozzi C. R. (2005). Glycans in Cancer and Inflammation − Potential
for Therapeutics and Diagnostics. Nat. Rev.
Drug Discovery.

[2] Laughlin S.
T., Bertozzi C. R. (2009). Imaging
the Glycome. Proc. Natl.
Acad. Sci. U. S. A..

[3] Reily C., Stewart T. J., Renfrow M. B., Novak J. (2019). Glycosylation in Health
and Disease. Nat. Rev. Nephrol..

[4] Schauer R. (2000). Achievements
and Challenges of Sialic Acid Research. Glycoconj.
J..

[5] Pinho S. S., Reis C. A. (2015). Glycosylation in
Cancer: Mechanisms and Clinical Implications. Nat. Rev. Cancer.

[6] Narayanan S. (1994). Sialic Acid
as a Tumor Marker. Ann. Clin. Lab. Sci..

[7] Fuster M. M., Esko J. D. (2005). The Sweet and Sour
of Cancer: Glycans as Novel Therapeutic
Targets. Nat. Rev. Cancer.

[8] Crocker P. R., Paulson J. C., Varki A. (2007). Siglecs and
Their Roles in the Immune
System. Nat. Rev. Immunol..

[9] Yogeeswaran G., Salk P. L. (1981). Metastatic Potential
Is Positively Correlated with
Cell Surface Sialylation of Cultured Murine Tumor Cell Lines. Science.

[10] Macauley M. S., Crocker P. R., Paulson J. C. (2014). Siglec-Mediated Regulation of Immune
Cell Function in Disease. Nat. Rev. Immunol..

[11] Zhou X., Yang G., Guan F. (2020). Biological
Functions and Analytical
Strategies of Sialic Acids in Tumor. Cells.

[12] Mechref Y., Novotny M. V. (2002). Structural Investigations
of Glycoconjugates at High
Sensitivity. Chem. Rev..

[13] Prescher J. A., Bertozzi C. R. (2005). Chemistry in Living
Systems. Nat. Chem. Biol..

[14] Prescher J. A., Bertozzi C. R. (2006). Chemical Technologies
for Probing Glycans. Cell.

[15] Xiong Y., Li M., Lu Q., Qing G., Sun T. (2017). Sialic Acid-Targeted
Biointerface Materials and Bio-Applications. Polymers.

[16] Wang D.-E., Yan J., Jiang J., Liu X., Tian C., Xu J., Yuan M.-S., Han X., Wang J. (2018). Polydiacetylene Liposomes
with Phenylboronic Acid Tags: A Fluorescence Turn-On Sensor for Sialic
Acid Detection and Cell-Surface Glycan Imaging. Nanoscale.

[17] Matsumoto A., Sato N., Kataoka K., Miyahara Y. (2009). Noninvasive Sialic
Acid Detection at Cell Membrane by Using Phenylboronic Acid Modified
Self-Assembled Monolayer Gold Electrode. J.
Am. Chem. Soc..

[18] Matsumoto A., Cabral H., Sato N., Kataoka K., Miyahara Y. (2010). Assessment
of Tumor Metastasis by the Direct Determination of Cell-Membrane Sialic
Acid Expression. Angew. Chem. Int. Ed..

[19] Deshayes S., Cabral H., Ishii T., Miura Y., Kobayashi S., Yamashita T., Matsumoto A., Miyahara Y., Nishiyama N., Kataoka K. (2013). Phenylboronic Acid-Installed Polymeric Micelles for
Targeting Sialylated Epitopes in Solid Tumors. J. Am. Chem. Soc..

[20] Crich S. G., Alberti D., Szabo I., Aime S. (2013). MRI Visualization of
Melanoma Cells by Targeting Overexpressed Sialic Acid with a Gd^III^-dota-en-pba Imaging Reporter. Angew.
Chem. Int. Ed..

[21] Liu H.-W., Law W. H.-T, Lee L. C.-C, Lau J. C.-W, Lo K. K.-W. (2017). Cyclometalated
Iridium­(III) Bipyridine–Phenylboronic Acid Complexes as Bioimaging
Reagents and Luminescent Probes for Sialic Acids. Chem. Asian J..

[22] Schauer R. (2009). Sialic Acids
as Regulators of Molecular and Cellular Interactions. Curr. Opin. Struct. Biol..

[23] Pancera M., Shahzad-Ul-Hussan S., Doria-Rose N. A., McLellan J. S., Bailer R. T., Dai K., Loesgen S., Louder M. K., Staupe R. P., Yang Y., Zhang B., Parks R., Eudailey J., Lloyd K. E., Blinn J., Alam S. M., Haynes B. F., Amin M. N., Wang L. X., Burton D. R., Koff W. C., Nabel G. J., Mascola J. R., Bewley C. A., Kwong P. D. (2013). Structural Basis
for Diverse *N*-Glycan Recognition by HIV-1–Neutralizing
V1–V2–Directed Antibody PG16. Nat. Struct. Mol. Biol..

[24] Wang X., Qian S., Wang D., Wang C., Qin H., Peng L., Lu W., Zhang Y., Qing G. (2021). Self-Assembly
Gel-Based Dynamic Response System for Specific Recognition of *N*-Acetylneuraminic Acid. J. Mater.
Chem. B.

[25] Roundhill D. M., Gray H. B., Che C. M. (1989). Pyrophosphito-Bridged
Diplatinum
Chemistry. Acc. Chem. Res..

[26] Houlding V. H., Miskowski V. M. (1991). The Effect
of Linear Chain Structure on the Electronic
Structure of Pt­(II) Diimine Complexes. Coord.
Chem. Rev..

[27] Yam V. W.-W., Chan L.-P., Lai T.-F. (1993). Syntheses, Photophysics, and X-ray
Structural Characterization of Dinuclear Platinum­(II) Acetylide Complexes,
[Pt_2_(μ-dppm)_2_(μ-PhC≡C)­(PhC≡C)_2_]­ClO_4_ and [Pt_2_(μ-dppm)_2_(μ-^t^BuC≡C)­(^t^BuC≡C)­Cl]­ClO_4_. Organometallics.

[28] Connick W. B., Henling L. M., Marsh R. E., Gray H. B. (1996). Emission Spectroscopic
Properties of the Red Form of Dichloro­(2,2’-bipyridine)­platinum­(II).
Role of Intermolecular Stacking Interactions. Inorg. Chem..

[29] Yam V. W.-W., Tang R. P.-L., Wong K. M.-C., Cheung K.-K. (2001). Synthesis, Luminescence,
Electrochemistry, and Ion-Binding Studies of Platinum­(II) Terpyridyl
Acetylide Complexes. Organometallics.

[30] Wong K. M.-C., Hui C.-K., Yu K.-L., Yam V. W.-W. (2002). Luminescence
Studies of Dinuclear Platinum­(II) Alkynyl Complexes and Their Mixed-Metal
Platinum­(II)–Copper­(I) and –Silver­(I) Complexes. Coord. Chem. Rev..

[31] Yam V. W.-W., Wong K. M.-C., Zhu N. (2002). Solvent-Induced Aggregation
through
Metal···Metal/π···π Interactions:
Large Solvatochromism of Luminescent Organoplatinum­(II) Terpyridyl
Complexes. J. Am. Chem. Soc..

[32] Wong K. M.-C., Yam V. W.-W. (2011). Self-Assembly
of Luminescent Alkynylplatinum­(II) Terpyridyl
Complexes: Modulation of Photophysical Properties through Aggregation
Behavior *Acc*. Chem. Res..

[33] Yam V. W.-W., Au V. K.-M., Leung S. Y.-L. (2015). Light-Emitting
Self-Assembled Materials
Based on d^8^ and d^10^ Transition Metal Complexes. Chem. Rev..

[34] Chan A. K.-W., Yam V. W.-W. (2018). Precise Modulation of Molecular Building
Blocks from
Tweezers to Rectangles for Recognition and Stimuli-Responsive Processes. Acc. Chem. Res..

[35] Moussa J., Haddouche K., Chamoreau L.-M., Amouri H., Gareth Williams J. A. (2016). New N^C^N-Coordinated
Pd­(II) and Pt­(II) Complexes of A Tridentate *N*-Heterocyclic
Carbene Ligand Featuring A 6-Membered Central Ring: Synthesis. Structures and Luminescence. Dalton Trans..

[36] Yam V. W.-W., Law A. S.-Y. (2020). Luminescent d^8^ Metal Complexes of Platinum­(II)
and Gold­(III): From Photophysics to Photofunctional Materials and
Probes. Coord. Chem. Rev..

[37] Yu C., Chan K. H.-Y, Wong K. M.-C, Yam V. W.-W. (2006). Single-Stranded
Nucleic Acid-Induced Helical Self-Assembly of Alkynylplatinum­(II)
Terpyridyl Complexes. Proc. Natl. Acad. Sci.
U. S. A..

[38] Yu C., Chan K. H.-Y, Wong K. M.-C., Yam V. W.-W. (2009). Nucleic Acid-Induced
Self-Assembly of A Platinum­(II) Terpyridyl Complex: Detection of G-Quadruplex
Formation and Nuclease Activity. Chem. Commun..

[39] Chung C. Y.-S., Chan K. H.-Y, Yam V. W.-W. (2011). “Proof-of-Principle”
Concept for Label-Free Detection of Glucose and α-Glucosidase
Activity through the Electrostatic Assembly of Alkynylplatinum­(II)
Terpyridyl Complexes. Chem. Commun..

[40] Yeung M. C.-L, Wong K. M.-C., Tsang Y. K. T, Yam V. W.-W. (2010). Aptamer-Induced
Self-Assembly of A NIR-Emissive Platinum­(II) Terpyridyl Complex for
Label- and Immobilization-Free Detection of Lysozyme and Thrombin. Chem. Commun..

[41] Chung C. Y.-S., Yam V. W.-W. (2011). Induced Self-Assembly and Förster Resonance
Energy Transfer Studies of Alkynylplatinum­(II) Terpyridine Complex
Through Interaction With Water-Soluble Poly­(phenylene ethynylene sulfonate)
and the Proof-of-Principle Demonstration of this Two-Component Ensemble
for Selective Label-Free Detection of Human Serum Albumin. J. Am. Chem. Soc..

[42] Chan C. W.-T, Cheng H.-K., Hau F. K.-W, Chan A. K.-W, Yam V. W.-W. (2019). Protamine-Induced
Supramolecular Self-Assembly of Red-Emissive Alkynylplatinum­(II) 2,6-Bis­(benzimidazol-2′-yl)­pyridine
Complex for Selective Label-Free Sensing of Heparin and Real-Time
Monitoring of Trypsin Activity. ACS Appl. Mater.
Interfaces.

[43] Yeung M. C.-L., Yam V. W.-W. (2013). Phosphate Derivative-Induced Supramolecular Assembly
and NIR-emissive Behaviour of Alkynylplatinum­(II) Terpyridine Complexes
for Real-Time Monitoring of Enzymatic Activities. Chem. Sci..

[44] Law A. S.-Y., Yeung M. C.-L., Yam V. W.-W. (2019). A Luminescence
Turn-On Assay for
Acetylcholinesterase Activity and Inhibitor Screening Based on Supramolecular
Self-Assembly of Alkynylplatinum­(II) Complexes on Coordination Polymer. ACS Appl. Mater. Interfaces.

[45] Law A. S.-Y, Lee L. C.-C, Yeung M. C.-L., Lo K. K.-W, Yam V. W.-W. (2019). Amyloid
Protein-Induced Supramolecular Self-Assembly of Water-Soluble Platinum­(II)
Complexes: A Luminescence Assay for Amyloid Fibrillation Detection
and Inhibitor Screening. J. Am. Chem. Soc..

[46] Law A. S.-Y, Lee L. C.-C, Lo K. K.-W, Yam V. W.-W. (2021). Aggregation and
Supramolecular Self-Assembly of Low-Energy Red Luminescent Alkynylplatinum­(II)
Complexes for RNA Detection, Nucleolus Imaging, and RNA Synthesis
Inhibitor Screening. J. Am. Chem. Soc..

[47] Ning Y., Jin G.-Q., Wang M.-X., Gao S., Zhang J.-L. (2022). Recent
Progress in Metal-Based Molecular Probes for Optical Bioimaging and
Biosensing. Curr. Opin. Chem. Biol..

[48] Hong G., Antaris A. L., Dai H. (2017). Near-Infrared
Fluorophores for Biomedical
Imaging. Nat. Biomed. Eng..

[49] Wong K. M.-C., Yam V. W.-W. (2007). Luminescence Platinum­(II) Terpyridyl Complexes—From
Fundamental Studies to Sensory Functions. Coord.
Chem. Rev..

[50] Yam V. W.-W., Chan A. K.-W., Hong E. Y.-H. (2020). Charge-Transfer Processes in Metal
Complexes Enable Luminescence and Memory Functions. Nat. Rev. Chem..

[51] Chan M. H.-Y., Yam V. W.-W. (2022). Toward the Design and Construction
of Supramolecular
Functional Molecular Materials Based on Metal–Metal Interactions. J. Am. Chem. Soc..

[52] Yam V. W.-W. (2023). Using
Synthesis to Steer Excited States and Their Properties and Functions. Nat. Synth..

[53] Zheng X., Chan M. H.-Y., Chan A. K.-W., Cao S., Ng M., Sheong F. K., Li C., Goonetilleke E. C., Lam W. W. Y., Lau T.-C., Huang X., Yam V. W.-W. (2022). Elucidation
of the Key Role of Pt···Pt Interactions in the Directional
Self-Assembly of Platinum­(II) Complexes. Proc.
Natl. Acad. Sci. U. S. A..

[54] Chung C. Y.-S., Li S. P.-Y., Lo K. K.-W, Yam V. W.-W. (2016). Synthesis and
Electrochemical, Photophysical, and Self-Assembly Studies on Water-Soluble
pH-Responsive Alkynylplatinum­(II) Terpyridine Complexes. Inorg. Chem..

[55] Po C., Tam A. Y.-Y., Yam V. W.-W. (2014). Tuning of Spectroscopic Properties *via* Variation of the Alkyl Chain Length: A Systematic Study
of Molecular Structural Changes on Self-Assembly of Amphiphilic Sulfonate-Pendant
Platinum­(II) Bzimpy Complexes in Aqueous Medium. Chem. Sci..

[56] Engin K., Leeper D. B., Cater J. R., Thistlethwaite A. J., Tupchong L., McFarlane J. D. (1993). Extracellular
pH Distribution in
Human Tumors. Int. J. Hyperthermia.

[57] Casey J. R., Grinstein S., Orlowski J. (2010). Sensors and Regulators
of Intracellular
pH. Nat. Rev. Mol. Cell Biol..

[58] Zhang X., Lin Y., Gillies R. J. (2010). Tumor pH
and Its Measurement. J. Nucl. Med..

[59] He X.-N., Wang Y.-N., Wang Y., Xu Z.-R. (2020). Accurate Quantitative
Detection of Cell Surface Sialic Acids with a Background-Free SERS
Probe. Talanta.

[60] Zhang X., Chen B., He M., Zhang Y., Peng L., Hu B. (2016). Boronic Acid Recognition
based-Gold Nanoparticle-Labeling Strategy
for the Assay of Sialic Acid Expression on Cancer Cell Surface by
Inductively Coupled Plasma Mass Spectrometry. Analyst.

[61] Dold J. E.
G. A., Wittmann V. (2021). Metabolic
Glycoengineering with Azide- and Alkene-Modified
Hexosamines: Quantification of Sialic Acid Levels. ChemBioChem..

[62] Gray M. A., Stanczak M. A., Mantuano N. R., Xiao H., Pijnenborg J. F. A., Malaker S. A., Miller C. L., Weidenbacher P. A., Tanzo J. T., Ahn G., Woods E. C., Läubli H., Bertozzi C. R. (2020). Targeted Glycan Degradation Potentiates the Anticancer
Immune Response *in vivo*. Nat.
Chem. Biol..

[63] Alshanski I., Sukhran Y., Mervinetsky E., Unverzagt C., Yitzchaik S., Hurevich M. (2021). Electrochemical Biosensing Platform
based on Complex Biantennary *N*-Glycan for Detecting
Enzymatic Sialylation Processes. Biosens. Bioelectron..

[64] Achterberg F. B., Sibinga Mulder B. G., Meijer R. P. J., Bonsing B. A., Hartgrink H. H., Mieog J. S. D., Zlitni A., Park S.-M., Farina
Sarasqueta A., Vahrmeijer A. l., Swijnenburg R.-J. (2020). Real-time
Surgical Margin Assessment Using ICG-Fluorescence during Laparoscopic
and Robot-Assisted Resections of Colorectal Liver Metastases. Ann. Transl. Med..

[65] Wakabayashi T., Cacciaguerra A. B., Abe Y., Bona E. D., Nicolini D., Mocchegiani F., Kabeshima Y., Vivarelli M., Wakabayashi G., Kitagawa Y. (2022). Indocyanine Green Fluorescence Navigation
in Liver Surgery: A Systematic Review on Dose and Timing of Administration. Ann. Surg..

