# Supplementary material for: Self-Assembly of Alkynylplatinum(II) Complexes for Sialic Acid Detection and Differentiation of Cancer Cells from Normal Cells
Source: J Am Chem Soc. 2025 Jun 12;147(25):21629–37. doi: 10.1021/jacs.5c03210 (PMC12203603; doi:10.1021/jacs.5c03210)
Supplement: Supplementary file 1 [file ja5c03210_si_001.pdf]

Supporting Information

Self-Assembly of Alkynylplatinum(II) Complexes for Sialic Acid Detection, and Differentiation of Cancer Cells from Normal Cells

Jungu Guo,[a,b] Eric Ka-Ho Wong,[a] Guang-Xi Xu,[c] Angela Sin-Yee Law,[a] Michael Ho-Yeung Chan,[a] Jonathan Lam,[a] Ziyong Chen,[a] Kenneth Kam-Wing Lo,[c] and Vivian Wing-Wah Yam*,[a]

[a] Institute of Molecular Functional Materials and Department of Chemistry, The University of Hong Kong, Pokfulam Road, Hong Kong 999077, People's Republic of China

[b] Chemistry and Chemical Engineering of Guangdong Provincial Laboratory, No. 1, College Road, Tuojiang Street, Jinping District, Shantou 515000, Guangdong, People's Republic of China

[c] Department of Chemistry, City University of Hong Kong, Tat Chee Avenue, Kowloon, Hong Kong 999077, People's Republic of China

*E-mail: wwyam@hku.hk

EXPERIMENTAL PROCEDURES

Materials and Reagents

1-Ethyl-3-(3-dimethylaminopropyl)carbodiimide hydrochloride (EDC-HCl, MACKLIN), 1-hydroxybenzotriazole (HOBt, J&K), 4-bromobenzoic acid (Aldrich), triethylamine (Thermo Fisher), copper(I) iodide (J&K), methyl L-histidinate (J&K), *N*-acetylneuraminic acid (Combi Block), polysialic acid (Biosynth Carbosynth), D-(+)-mannose (Sigma-Aldrich), D-(+)-glucose (Sigma-Aldrich), *β*-lactose (Sigma-Aldrich), sucrose (Sigma-Aldrich), and D-galactose (Sigma-Aldrich) were purchased from the corresponding chemical company. PolySia was purchased from Biosynth Carbosynth which is a linear small polysaccharide containing α-2,8-linked sialic acid with 8 to >100 residues with an average molecular weight of 50000–60000 g mol^{-1}. The concentration was represented by the concentration of monomer unit in polySia. Molar equivalence is determined based on the concentration of the repeating unit, indicating that 30 μM is equal to 1 equivalent. HeLa cells, HepG2 cells and HEK293T cells were obtained from American Type Culture Collection (ATCC). FITC-conjugated lectin, Dulbecco's Modified Eagle's medium (DMEM), Ham's F-12 nutrient mixture, fetal bovine serum (FBS), PBS buffer, and 3-(4,5-dimethyl-2-thiazolyl)-2,5-diphenyl-2*H*-tetrazolium bromide (MTT) were purchased from Invitrogen. HEK293T is a derivative of the Human Embryonic Kidney (HEK) 293 cell line, established by the expression of T antigen of simian virus 40. HepG2 is a human liver cancer cell line. HeLa cell is a cervical cancer cell line. All other reagents and solvents were of analytical grade and were used without further purification. Deionized water used was purified with Elga Purelab UHQ system. [Pt(tpy)Cl](PF_6), [Pt{tpy-($C_6H_4OC_{12}H_{25}$-4)-4'}Cl](PF_6), [Pt{tpy-($C_6H_4CH_2NMe_3$-4)-4'}Cl](PF_6) and [Pt{tpy-($C_6H_4CH_2NMe_2$-4)-4'}Cl](PF_6) were synthesized according to a modification of literature procedures.[1]

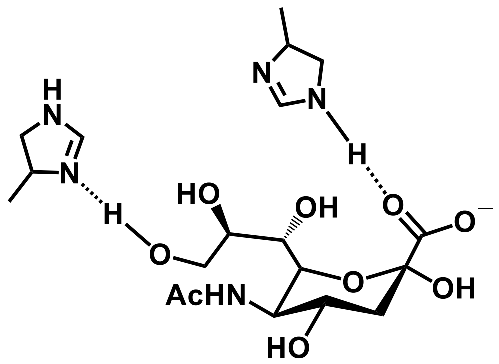

Scheme S1. Schematic diagram showing the hydrogen bonding interactions between histidine moiety and sialic acid.

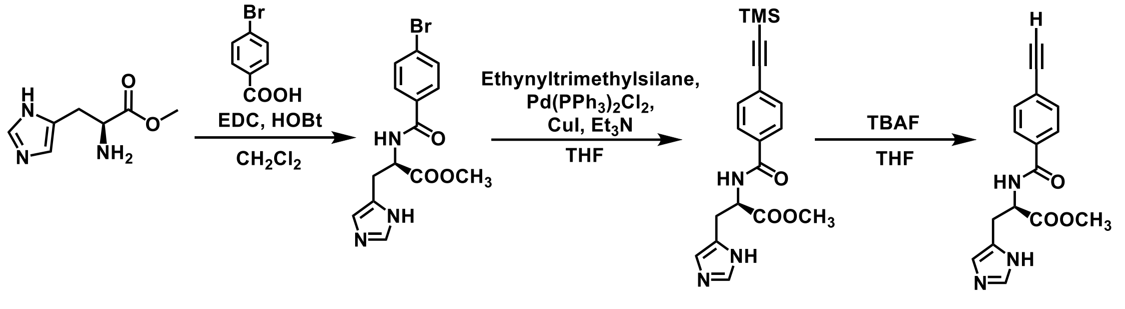

L1 L2 L3

Scheme S2. Synthetic route of **L3**.

Synthesis of L1. Methyl L-histidinate (1.43 g, 5.91 mmol) and triethylamine (2.06 mL, 14.79 mmol) were dissolved in dichloromethane (150 mL). 4-Bromobenzoic acid (0.99 g, 4.93 mmol), EDC-HCl (1.23 g, 6.41 mmol) and HOBt (0.87 g, 6.41 mmol) were added and the reaction mixture was allowed to stir overnight. The reaction mixture was then washed with saturated $NaHCO_3$ solution for three times. The organic layer was dried over anhydrous $MgSO_4$, filtered and evaporated under reduced pressure. The crude product was purified by column chromatography on silica gel using ethyl acetate–methanol (40:1, v/v) as the eluent. The product was obtained as a white solid (1.50 g, 76%). ^{1}H NMR (500 MHz, $CDCl_3$, 298 K, δ/ppm): δ 8.31 (s, 1H, imidazole), 7.81 (d, 2H, J = 8.0 Hz, $-C_6H_4-$), 7.63 (d, 2H, J = 8.0 Hz, $-C_6H_4-$), 7.62 (s, 1H, imidazole), 6.85 (s, 1H, –CON*H*–), 4.97 (m, 1H, –C*H*(NH)–), 3.70 (s, 3H, $-CH_3$), 3.27 (m, 2H, $-CH_2-$). HRMS (positive-ion ESI) cald. for $[C_{14}H_{14}N_3O_3BrNa]^+$ m/z = 374.0111; found: 374.0136 $[M+Na]^+$.

Synthesis of L2. A mixture of **L1** (1.30 g. 3.51 mmol), bis-(triphenylphosphine)palladium(II) chloride (123 mg, 0.175 mmol) and copper(I) iodide (33 mg, 175 mmol) in distilled triethylamine (15 mL) and THF (70 mL) was purged with nitrogen. Tri(methylsilyl)acetylene (1.49 mL, 10.52 mmol) was then added and the reaction mixture was heated under reflux overnight. The reaction mixture was then filtered and the solvent was removed under reduced pressure. The crude product was then purified by column chromatography on silica gel using ethyl acetate–methanol (40:1, v/v) as the eluent. The product was obtained as a yellow solid (0.80 g, 77%). ^{1}H NMR (500 MHz, $CDCl_3$, 298 K, *δ*/ppm): *δ* 8.18 (m, 1H, imidazole), 7.83 (d, 2H, *J* = 8.0 Hz, $-C_6H_4-$), 7.63 (s, 1H, imidazole), 7.53 (d, 2H, *J* = 8.0 Hz, $-C_6H_4-$), 6.86 (s, 1H, –CON*H*–), 4.99 (m, 1H, –C*H*(NH)–), 3.71 (s, 3H, $-CH_3$), 3.28 (m, 2H, $-CH_2-$), 0.26 (s, 9H, $-Si(CH_3)_3$). HRMS (positive-ion ESI) cald. for $[C_{19}H_{23}N_3O_3Si]^+$ m/z = 370.1581; found: 370.1585 $[M+H]^+$.

Synthesis of L3. To a stirred solution of **L2** (0.69 g, 1.87 mmol) in methanol (30 mL) was added K_2CO_3 (0.77 g, 5.60 mmol). The mixture was stirred for 6 h and the solvent was removed under reduced pressure. The crude product was then purified by column chromatography on silica gel using ethyl acetate–methanol (40:1, v/v) as the eluent. The product was obtained as a white solid (0.50 g, 90%).^{1}H NMR (500 MHz, DMSO-d_6, 298 K, *δ*/ppm): *δ* 11.84 (s, 1H, –NH–), 8.96 (m, 1H, imidazole), 7.84 (d, 2H, *J* = 8.0 Hz, $-C_6H_4-$), 7.59 (d, 2H, *J* = 8.0 Hz, $-C_6H_4-$), 7.58 (s, 1H, imidazole), 6.86 (s, 1H, –CON*H*–), 4.67 (m, 1H, –C*H*(NH)–), 4.39 (s, 1H, HC≡C–), 3.62 (s, 3H, $-CH_3$), 3.05 (m, 2H, $-CH_2-$). HRMS (positive-ion ESI) cald. For $[C_{16}H_{15}N_3O_3Na]^+$ m/z = 320.1006; found: 320.1007 $[M+Na]^+$.

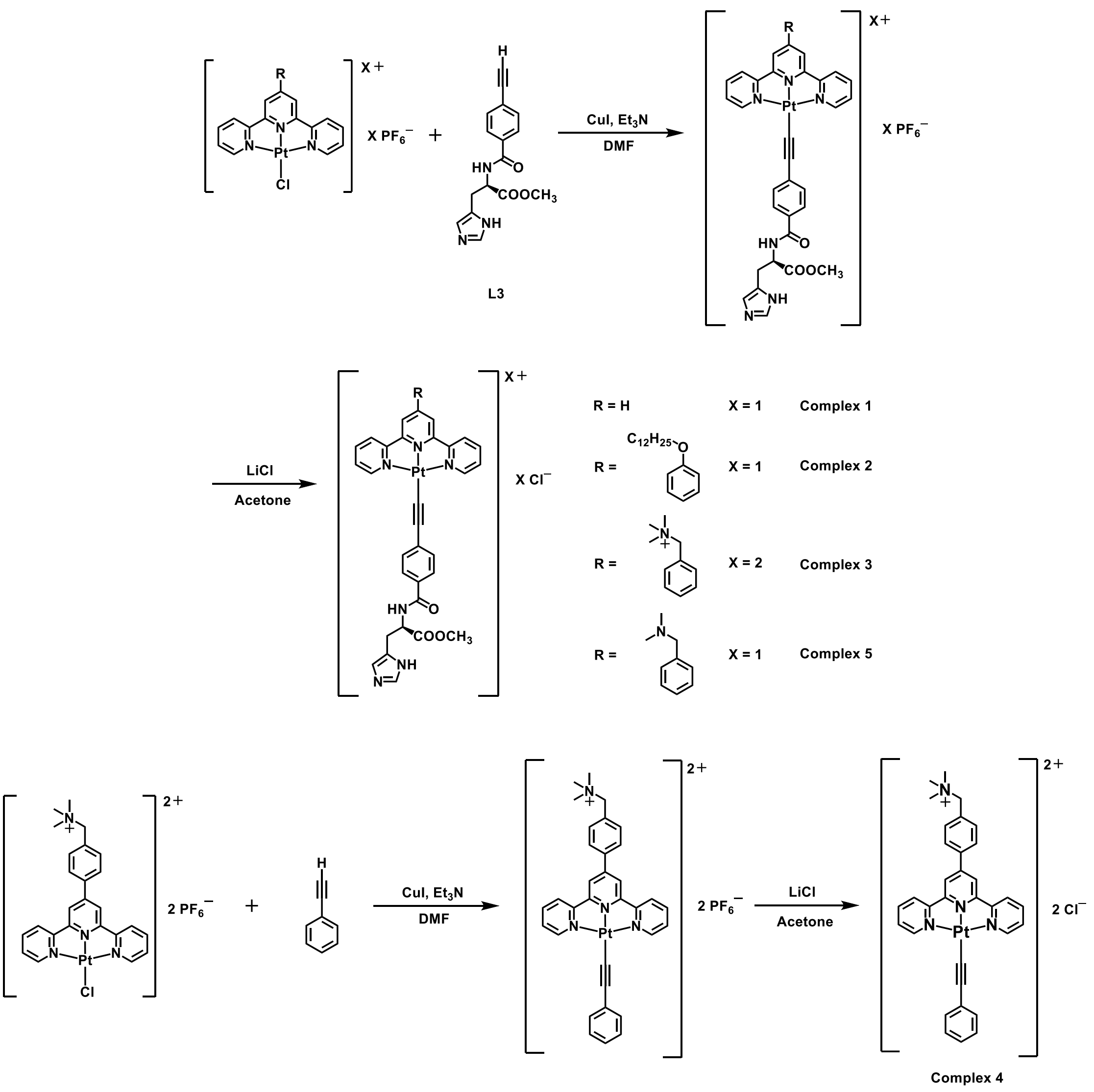

Scheme S3. Synthetic route of **1–5**.

Synthesis of 1. To a solution of [Pt(tpy)Cl](PF_6) (0.12 g, 0.17 mmol) in degassed DMF (6 mL) were added triethylamine (1 mL), **L3** (0.15 g, 0.51 mmol) and a catalytic amount of CuI. The reaction mixture was allowed to stir at room temperature under inert atmosphere. After reaction overnight, the mixture was dropwisely added into diethyl ether and the precipitate formed was collected by filtration. The crude product was dissolved in acetone and 1.5 equiv. of LiCl (0.87 mg, 0.26 mmol) was added into the solution. The obtained precipitate was collected and subsequent recrystallization by diffusion of diethyl ether vapor into a concentrated methanol–acetonitrile solution of the crude product which was washed successively with dichloromethane and diethyl ether. The final product was obtained as a dark-brown solid. Yield: 35 mg (27%). ^{1}H

NMR (500 MHz, DMSO-d_6, 298 K, δ/ppm): δ 9.22 (d, J = 8.6 Hz, 2H, terpyridine), 8.93 (s, 1H, imidazole), 8.71 (t, J = 8.6 Hz, 4H, terpyridine), 8.67 (m, 1H, imidazole), 8.64 (t, J = 8.6 Hz, 1H, terpyridine), 8.55 (t, J = 8.6 Hz, 2H, terpyridine), 7.97 (t, J = 8.6 Hz, 2H, terpyridine), 7.85 (m, 2H, $-C_6H_4-$), 7.63 (m, 2H, $-C_6H_4-$), 4.70 (s, 1H, $-CH$(NH)$-$), 3.63 (s, 3H, $-CH_3$), 3.06 (m, 2H, $-CH_2-$). HRMS (positive-ion ESI) cald. for $[C_{31}H_{25}N_6O_3Pt]^+$ m/z = 724.1633; found: 724.1626 $[M-Cl]^+$.

Synthesis of 2. The procedure was similar to that of **1** except that [Pt{tpy-($C_6H_4OC_{12}H_{25}$-4)-4'}Cl](PF_6) (0.10 g, 0.12 mmol) was used instead of [Pt(tpy)Cl](PF_6). The product was obtained as an orange solid. Yield: 35 mg (20%). ^{1}H NMR (500 MHz, DMSO-d_6, 298 K, δ/ppm): δ 9.21 (d, J = 8.6 Hz, 2H, terpyridine), 9.02 (m, 2H, terpyridine), 8.98 (m, 2H, terpyridine), 8.90 (m, 1H, imidazole), 8.90 (m, 2H, terpyridine), 8.58 (t, 2H, J = 8.6 Hz, $-C_6H_4-$), 8.24 (t, 2H, J = 8.6 Hz, terpyridine), 7.99 (m, 2H, $-C_6H_4-$), 7.85 (m, 1H, imidazole), 7.62 (m, 2H, $-C_6H_4-$), 7.23 (d, 2H, J = 8.0 Hz, $-C_6H_4-$), 4.12 (t, 2H, J = 4.12 Hz, $-O(CH_2)-$), 3.64 (s, 3H, $-CH_3$), 3.07 (s, 2H, $-CH_2-$), 1.79 (m, 2H, $-CH_2-$), 1.45 (m, 2H, $-CH_2-$), 1.26 (m, 16H, $-CH_2-$), 0.87 (t, 3H, J = 0.85 Hz, $-CH_3$). HRMS (positive-ion ESI) cald. for $[C_{49}H_{53}N_6O_4Pt]^+$ m/z = 984.3774; found: 984.3817 $[M-Cl]^+$.

Synthesis of 3. The procedure was similar to that of **1** except that [Pt{tpy-($C_6H_4CH_2NMe_3$-4)-4'}Cl](PF_6) (0.10 g, 0.11 mmol) was used instead of [Pt(tpy)Cl](PF_6). The product was obtained as a dark-brown solid. Yield: 36 mg (35%). ^{1}H NMR (500 MHz, DMSO-d_6, 298 K, δ/ppm): δ 9.27 (d, J = 8.6 Hz, 2H, terpyridine), 9.14 (s, 2H, terpyridine), 8.92 (d, J = 8.6 Hz, 2H, terpyridine), 8.92 (m, 1H, imidazole), 8.63 (t, J = 8.6 Hz, 2H, terpyridine), 8.36 (d, J = 8.0 Hz, 2H, $-C_6H_4-$), 8.01 (t, J = 8.6 Hz, 2H, terpyridine), 7.87 (m, 4H, $-C_6H_4-$), 7.87 (m, 1H, imidazole), 7.65 (m, 2H, $-C_6H_4-$), 4.65 (s, 1H, $-CH$(NH)$-$), 4.65 (s, 2H, $-CH_2N^+(CH_3)_3$), 3.63 (s, 3H, $-CH_3$), 3.11 (s, 9H, $-N^+(CH_3)_3$). HRMS (positive-ion ESI) cald. for $[C_{41}H_{39}N_7O_3Pt]^{2+}$ m/z = 436.1377; found: 436.1368 $[M-2Cl]^{2+}$.

Synthesis of 4. The procedure was similar to that of **3** except that phenylacetylene (36

μL, 0.33 mmol) was used instead of **L3**. The product was obtained as a dark-red solid. Yield: 19 mg (23%). ^{1}H NMR (500 MHz, DMSO-d_6, 298 K, δ/ppm): δ 9.29 (d, J = 8.6 Hz, 2H, terpyridine), 9.15 (s, 2H, terpyridine), 8.93 (d, J = 8.6 Hz, 2H, terpyridine), 8.64 (t, J = 8.6 Hz, 2H, terpyridine), 8.37 (d, J = 8.0 Hz, 2H, $-C_6H_4-$), 8.03 (t, J = 8.6 Hz, 2H, terpyridine), 7.87 (d, J = 8.0 Hz, 2H, $-C_6H_4-$), 7.56 (d, J = 8.0 Hz, 2H, $-C_6H_5$), 7.40 (m, 2H, $-C_6H_5$), 7.31 (m, 1H, $-C_6H_5$), 4.66 (s, 2H, $-C\mathit{H}_2N^+(CH_3)_3$), 3.11 (s, 9H, $-CH_2N^+(C\mathit{H}_3)_3$). HRMS (positive-ion ESI) cald. for $[C_{33}H_{30}N_4Pt]^{2+}$ m/z = 338.6055; found: 338.6061 $[M-2Cl]^{2+}$.

Synthesis of 5. The procedure was similar to that of **1** except that [Pt{tpy-($C_6H_4CH_2NMe_2$-4)-4'}Cl](PF_6) (0.10 g, 0.13 mmol) was used instead of [Pt(tpy)Cl](PF_6). The product was obtained as a dark-brown solid. Yield: 10.7 mg (9.2%). ^{1}H NMR (500 MHz, DMSO-d_6, 298 K, δ/ppm): δ 9.24 (m, 2H, terpyridine), 9.11 (s, 2H, terpyridine) 8.92 (d, J = 8.6 Hz, 2H, terpyridine), 8.92 (m, 1H, imidazole), 8.61 (t, 2H, J = 8.6 Hz, terpyridine), 8.27 (d, 2H, J = 8.0 Hz, $-C_6H_4-$), 7.98 (t, 2H, J = 8.6 Hz, terpyridine), 7.98 (m, 1H, imidazole), 7.85 (m, 2H, $-C_6H_4-$), 7.78 (m, 2H, $-C_6H_4-$), 7.64 (s, 2H, $-C_6H_4-$), 4.73 (m, 1H, $-C\mathit{H}(NH)-$), 4.18 (m, 2H, $-C\mathit{H}_2N(CH_3)_2$), 3.65 (s, 6H, $-CH_2N(C\mathit{H}_3)_2$). HRMS (positive-ion ESI) cald. for $[C_{40}H_{36}N_7O_3Pt]^+$ m/z = 857.2525; found: 857.2521 $[M-Cl]^+$.

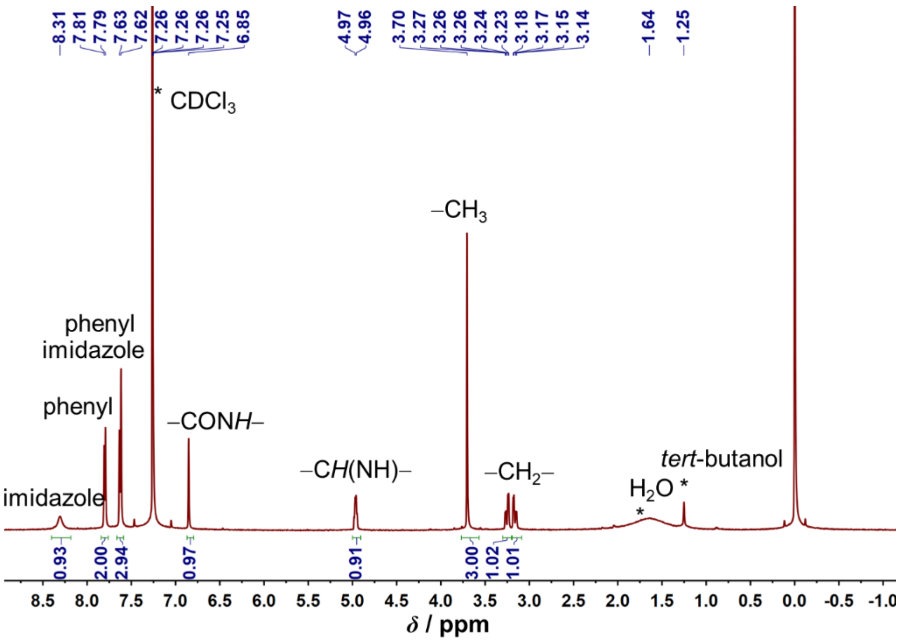

Figure S1. ^{1}H NMR spectrum of **L1** in $CDCl_3$ at 298 K.

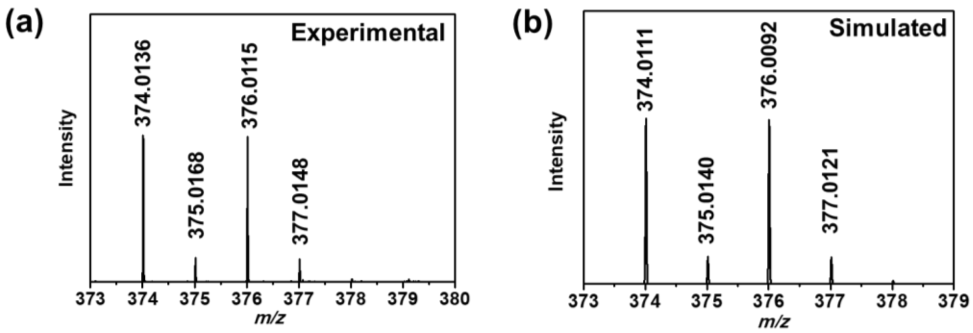

Figure S2. (a) Expanded ion cluster of high-resolution positive electrospray ionization (ESI) mass spectrum of **L1**, and (b) the corresponding simulated isotope pattern of $[M+Na]^+$.

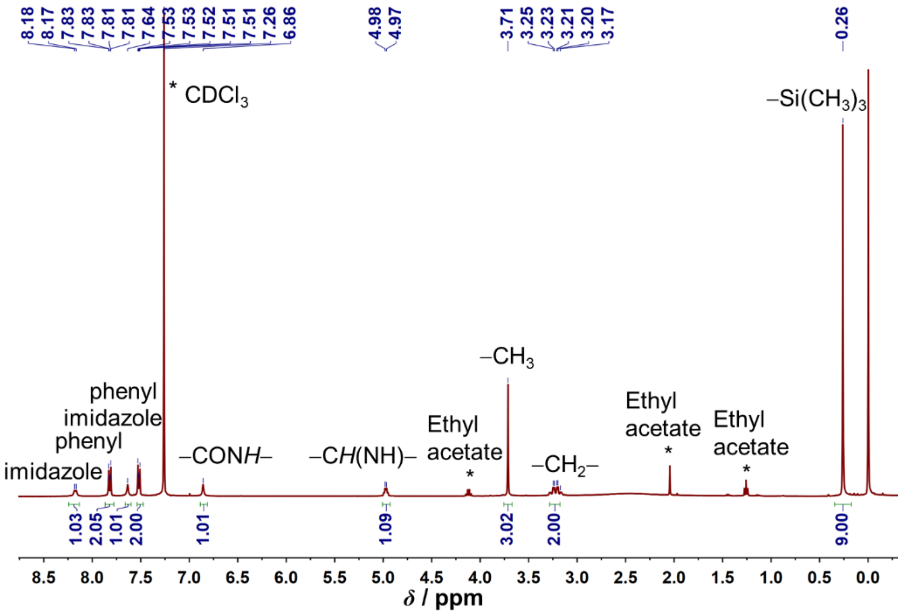

Figure S3. ^{1}H NMR spectrum of **L2** in $CDCl_3$ at 298 K.

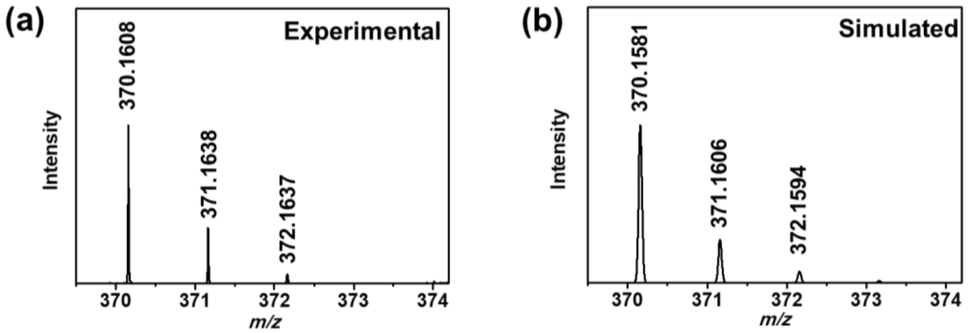

Figure S4. (a) Expanded ion cluster of high-resolution positive electrospray ionization (ESI) mass spectrum of **L2**, and (b) the corresponding simulated isotope pattern of $[M+H]^{+}$.

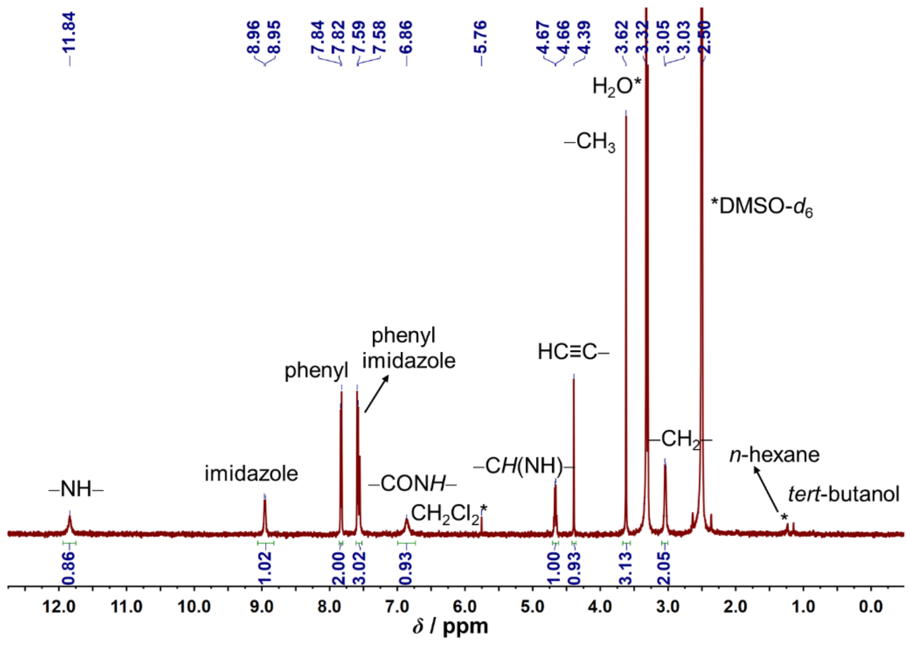

Figure S5. ^{1}H NMR spectrum of **L3** in DMSO-d_6 at 298 K.

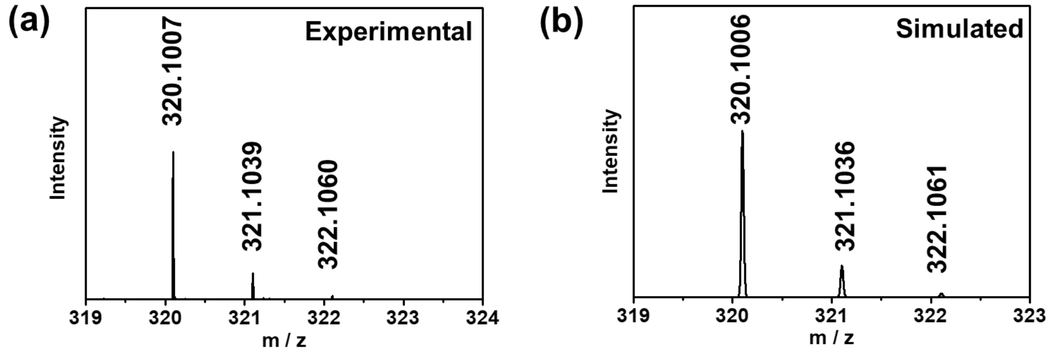

Figure S6. (a) Expanded ion cluster of high-resolution positive electrospray ionization (ESI) mass spectrum of **L3**, and (b) the corresponding simulated isotope pattern of $[M+Na]^{+}$.

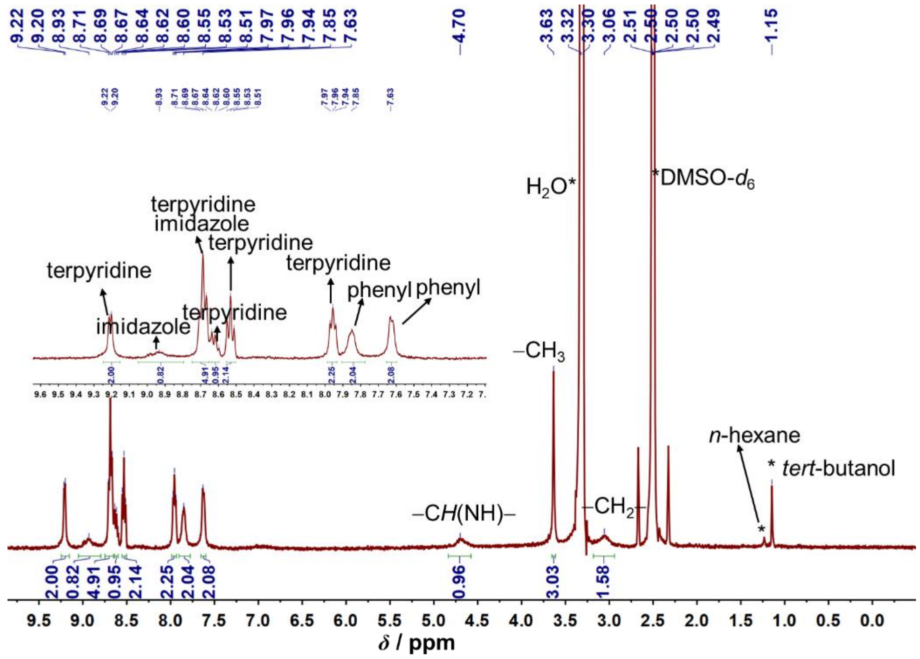

Figure S7. ^{1}H NMR spectrum of **1** in DMSO-d_6 at 298 K.

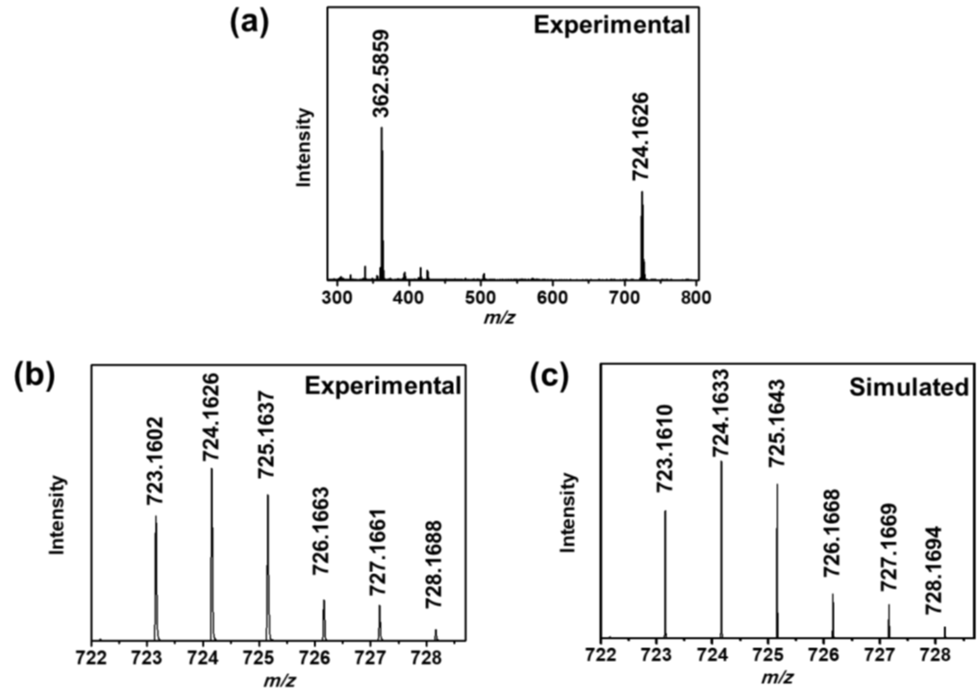

Figure S8. (a) High-resolution positive electrospray ionization (ESI) mass spectrum of **1**. (b) Expanded ion cluster of high-resolution positive electrospray ionization (ESI) mass spectrum of **1** and (c) the corresponding simulated isotope pattern of $[M{-}Cl]^+$.

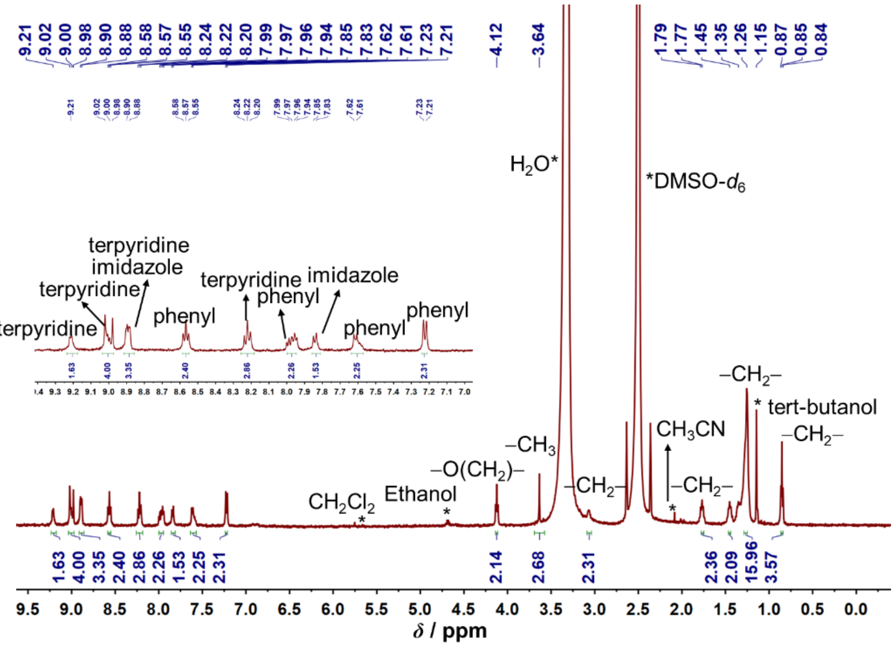

Figure S9. ^{1}H NMR spectrum of **2** in DMSO-d_6 at 298 K.

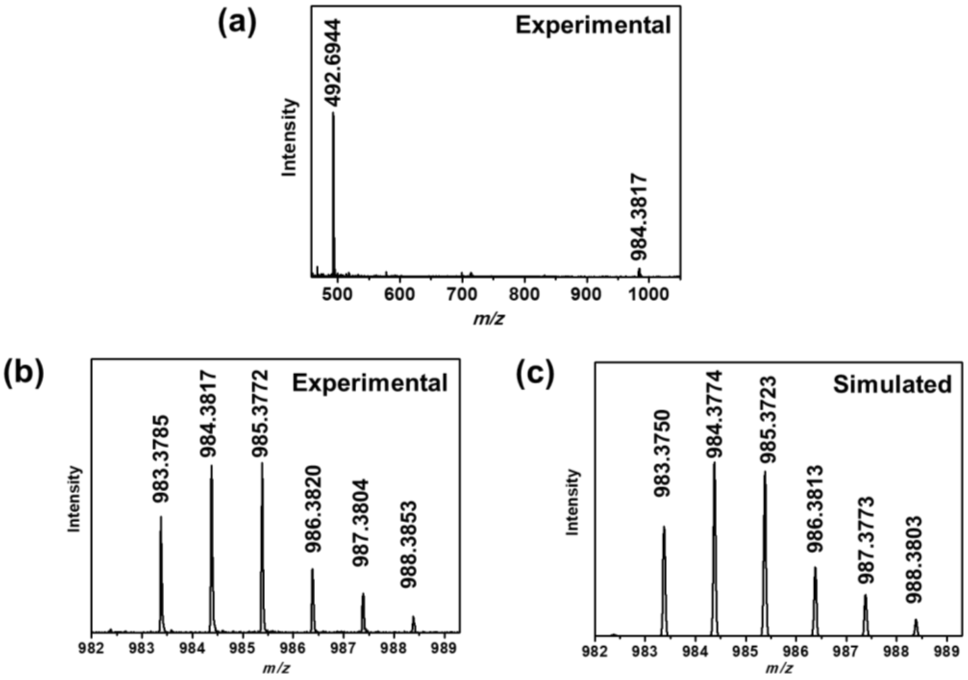

Figure S10. (a) High-resolution positive electrospray ionization (ESI) mass spectrum of **2**. (b) Expanded ion cluster of high-resolution positive electrospray ionization (ESI) mass spectrum of **2** and (c) the corresponding simulated isotope pattern of $[M{-}Cl]^+$.

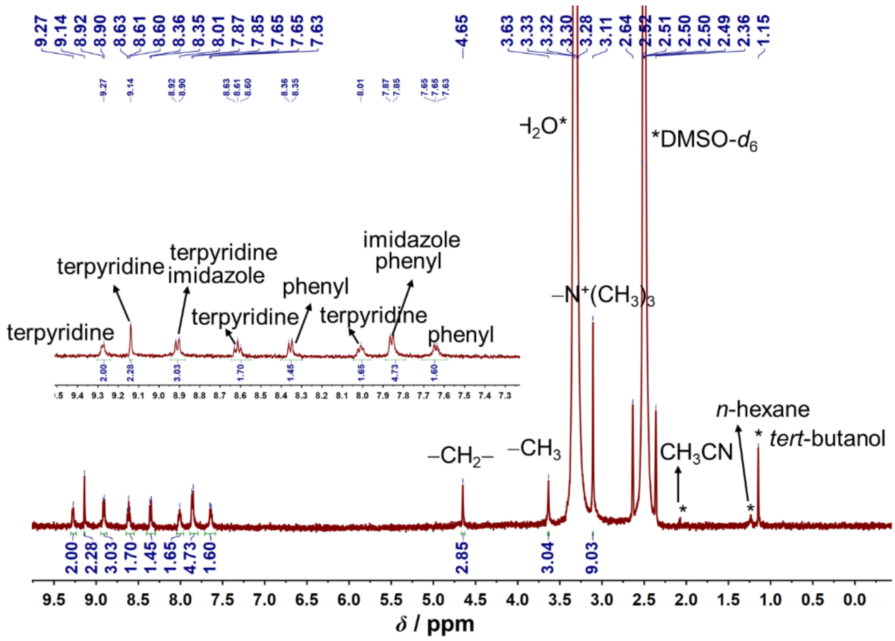

Figure S11. ^1H NMR spectrum of **3** in DMSO-d_6 at 298 K.

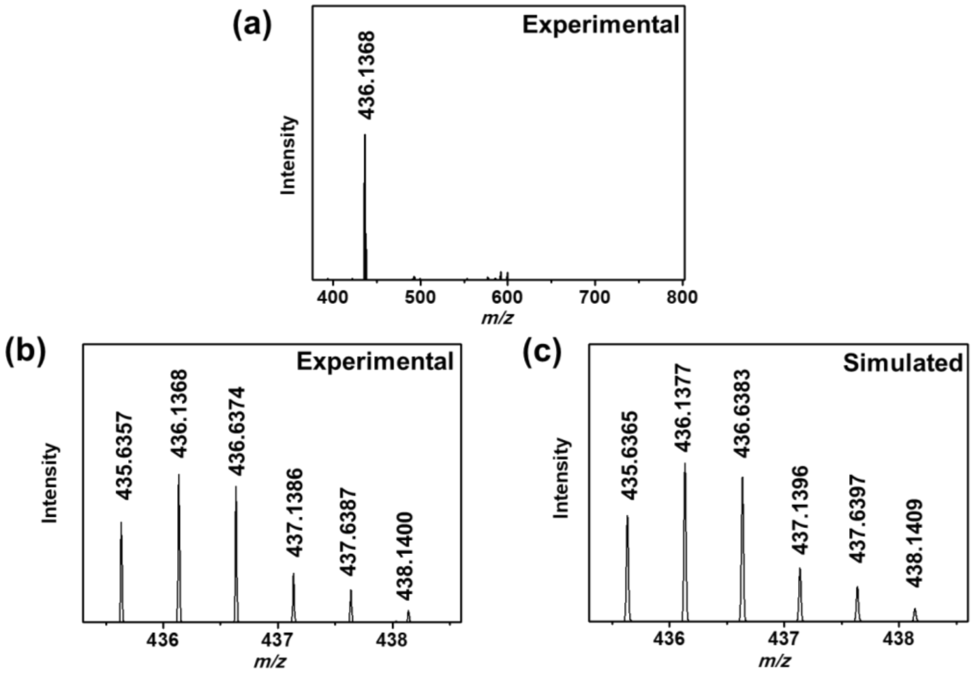

Figure S12. (a) High-resolution positive electrospray ionization (ESI) mass spectrum of **3**. (b) Expanded ion cluster of high-resolution positive electrospray ionization (ESI) mass spectrum of **3** and (c) the corresponding simulated isotope pattern of $[M{-}2Cl]^{2+}$.

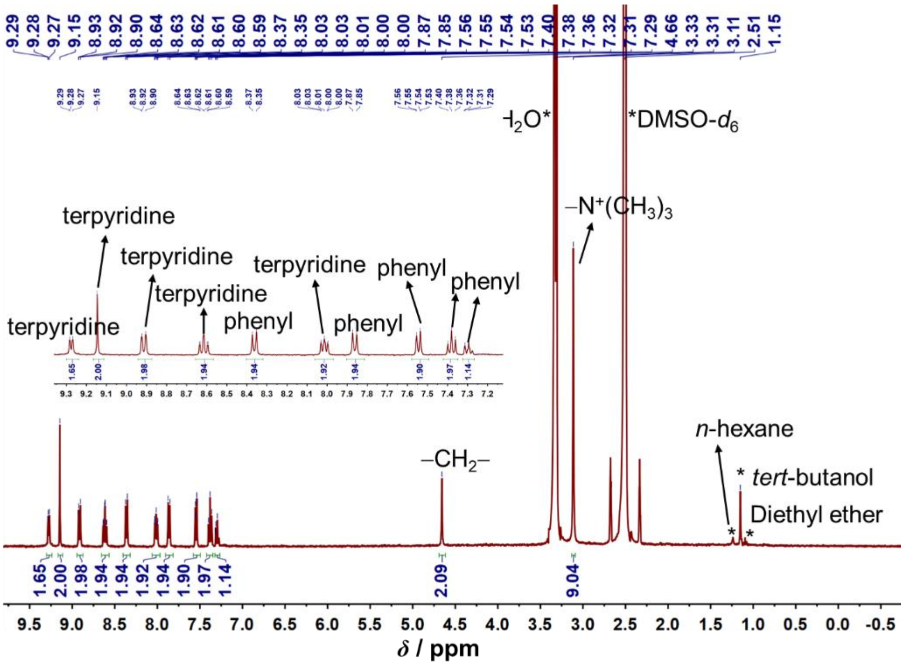

Figure S13. ^{1}H NMR spectrum of **4** in DMSO-d_6 at 298 K.

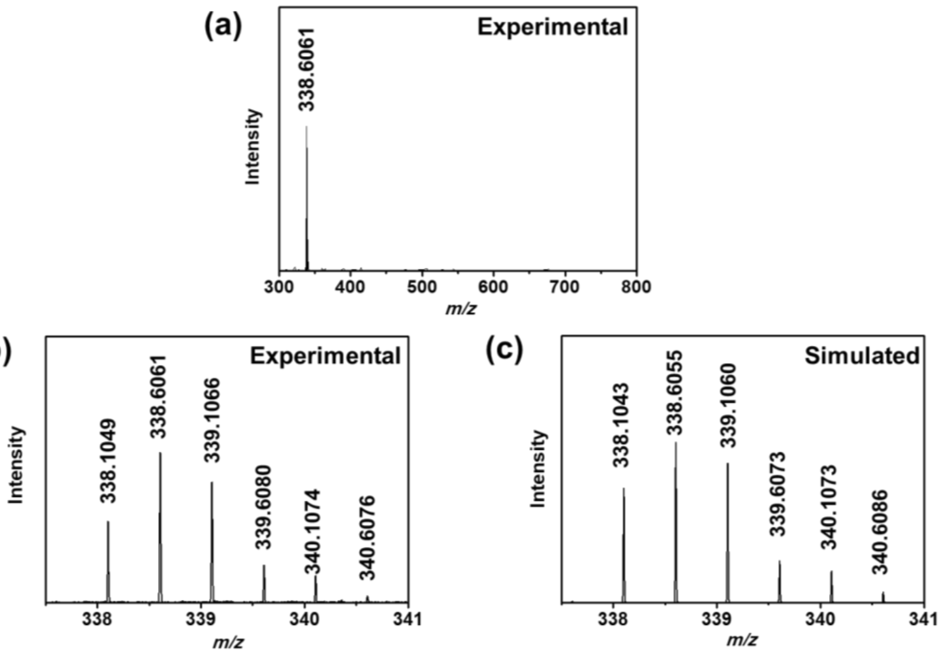

Figure S14. (a) High-resolution positive electrospray ionization (ESI) mass spectrum of **4**. (b) Expanded ion cluster of high-resolution positive electrospray ionization (ESI) mass spectrum of **4** and (c) the corresponding simulated isotope pattern of $[M{-}2Cl]^{2+}$.

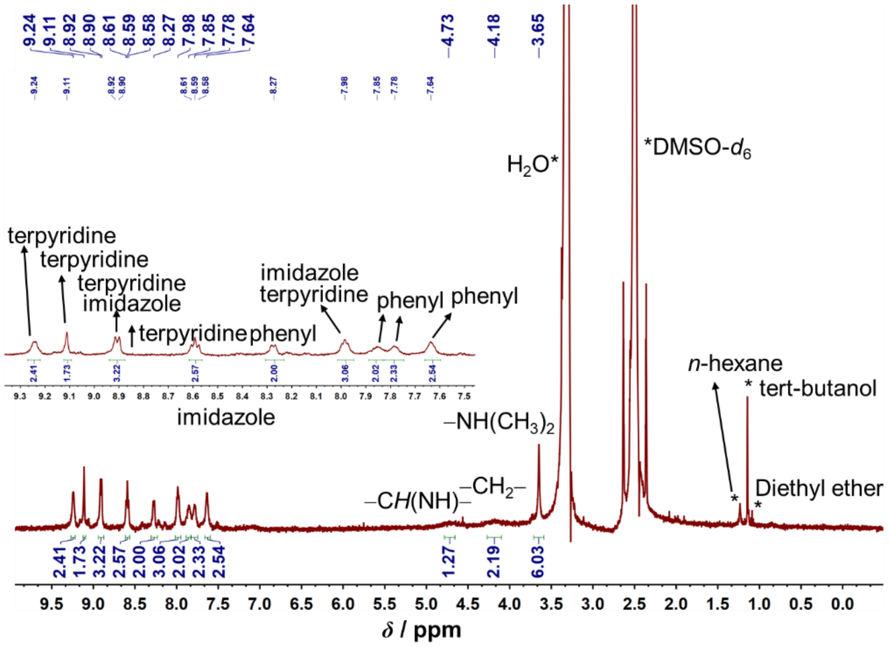

Figure S15. ^{1}H NMR spectrum of **5** in DMSO-d_6 at 298 K.

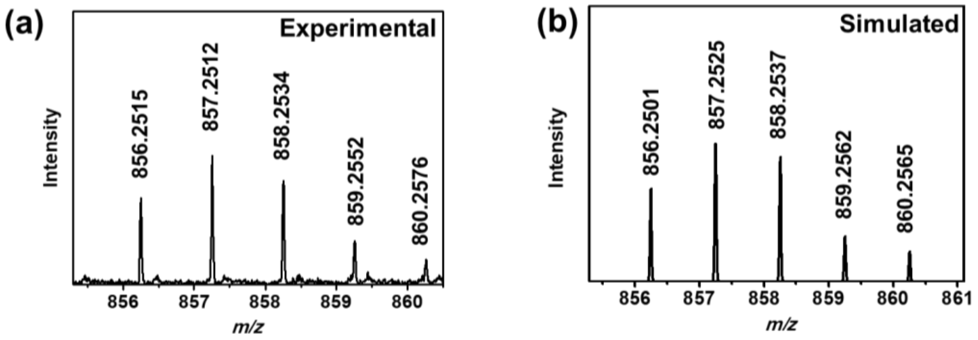

Figure S16. (a) Expanded ion cluster of high-resolution positive electrospray ionization (ESI) mass spectrum of **5** and (b) the corresponding simulated isotope pattern of $[M–Cl]^+$.

Physical Measurements and Instrumentation

^{1}H NMR spectra were recorded on a Bruker AVANCE 500 Fourier Transform NMR Spectrometer (500 MHz) with tetramethylsilane as an internal standard. Positive-ion and electrospray ionization (ESI) mass spectra were recorded on a Bruker maXis II high-resolution ESI-QTOF mass spectrometer. UV–Vis absorption spectra were collected on an Agilent Cary 60 UV–Vis Spectrophotometer with a Xenon flash lamp.

The emission spectra were recorded on an a Spex Fluorolog-3 model FL3-211 fluorescence spectrofluorometer equipped with an R2659P PMT detector. Dynamic light scattering (DLS) data and zeta potential data were collected on a Malvern Zetasizer Nano ZS90 equipped with an internal HeNe laser (λ_o = 633.0 nm) at 298 K. Transmission electron microscopy (TEM), scanning transmission electron microscopy (STEM), and scanning transmission electron microscopy−energy-disperse X-ray spectroscopy (STEM−EDX) elemental mapping experiments were performed on a FEI Tecnai G2 20 S-TWIN Scanning Transmission Electron Microscope with an accelerating voltage of 200 kV. The microscope was equipped with a Gatan ORIUS SC600 Model 831 CCD Camera (2.7 K × 2.7 K pixel) for digital imaging. Confocal microscopy experiments were performed on a Leica TCS SPE Confocal Scanning Microscope.

Spectroscopic Studies on 1–5 upon Addition of Different Concentration of Neu5Ac and PolySia

Different concentrations of Neu5Ac and polySia was added to a solution of **1–5** (30 μM) in Tris−HCl buffer (10 mM Tris, 10 mM NaCl, pH 8.0). The UV−vis absorption spectra, emission spectra, DLS data and zeta potential data were then recorded at 25 °C upon addition of increasing concentrations of Neu5Ac and polySia. The emission spectra of **1–5** were recorded with the respective excitation wavelength (**1**: λ_{ex} = 325 nm; **2**: λ_{ex} = 330 nm; **3**: λ_{ex} = 530 nm; **4**: λ_{ex} = 330 nm; **5**: λ_{ex} = 530 nm).

Determination of Apparent Binding Constant Between 1–5 and PolySia and Analysis for Cooperativity of Binding

To a solution of **1–5** (30 μM) in Tris−HCl buffer (10 mM Tris, 10 mM NaCl, pH 8.0), different concentrations of polySia were added. The emission spectra were recorded at 25 °C at an excitation wavelength of 530 nm.

The emission intensity at the emission maximum was fitted to the Hill equation below:

$$y = \frac{x^n}{K_d + x^n}$$

Wherein:

y stands for the corrected emission intensity; x stands for the concentration of polySia; n stands for the Hill coefficient, which can be used to indicate the cooperativity of binding to polySia; K_d stands for the apparent dissociation constant.

Self-Assembly Studies on the Complexes upon Addition of PolySia

TEM samples were prepared from an aqueous solution containing 30 μM platinum(II) complexes with or without 30 μM polySia. 5 μL of solution was then drop-cast onto carbon-coated copper grids using an autopipette. The copper grids were air dried overnight before measurement.

The samples for confocal imaging were prepared by mixing platinum(II) complexes with 3 equiv. of polySia in the Tris–HCl buffer (10 mM Tris, 10 mM NaCl, pH = 8.0). The confocal images were taken under a 63× objective using a solid-state laser with an excitation wavelength of 405 nm, and the emission was collected at 650–750 nm.

The samples for DLS and zeta potential measurement were prepared by mixing platinum(II) complexes with different concentrations of polySia in the Tris–HCl buffer (10 mM Tris, 10 mM NaCl, pH = 8.0).

Determination of p*K*a

The pK_a values of complexes was obtained using a revised version of Henderson-Hasselbach equation below:[2]

$$pK_a = pH + log\frac{[y_{acid} - y]}{[y - y_{base}]}$$

wherein:

y stands for absorbance at 610 nm; y_{acid} and y_{base} stand for the absorbance of absolute acid form and absolute conjugate base form, respectively.

LOD Assay

The limit of detection (LOD) of **3** for the detection of polySia has been determined by the emission titration spectra using the method based on the signal-to-noise ratio (S/N), following the equation 3σ/k, where σ is the standard deviation of the blank acquired from 30 independent assays and k is the slope of the linear calibration curve.

Selectivity Assay

1, 2, 3 equiv. of polySia and/or 1, 5, 10 equiv. of other monosaccharides (mannose, glucose, lactose, sucrose, galactose and Neu5Ac) were added to a solution of **3** (30 μM) in Tris–HCl buffer (10 mM Tris, 10 mM NaCl, pH 8.0). The emission spectra were recorded at 25 °C with an excitation wavelength of 530 nm.

Cell Culture

HeLa cells, HepG2 cells, and HEK293T cells were cultured with DMEM supplemented with 10% FBS and 1% penicillin/streptomycin in a humidified incubator at 37 °C where the carbon dioxide level was kept constant at 5%. They were subcultured every 2 to 3 days.

Confocal Imaging of Live Cells Stained with Platinum(II) Complexes

Concentration-dependent imaging assays were conducted as follows: Live HepG2 cells were adhered onto a sterile cover-slip in a 35-mm cell culture dish and were cultured for 48 h. The cells were washed in PBS buffer (1 mL) for three times and a solution of **3** (10 μM, 20 μM, 30 μM) in PBS buffer was applied and the cells were incubated at 37 °C for 0.5 h. After staining, the labeling solution was removed and the cells were washed in PBS buffer (1 mL) for three times. The confocal images were taken under a 63× objective using a solid-state laser with an excitation wavelength of 405 nm, and emission was collected at 700–800 nm.

The procedure of time-dependent imaging assays was similar to that of concentration-dependent assays except that the incubation time of cells with **3** (10 μM) were 0.5 h, 1 h, 2h, 6 h, 8 h.

The procedure of confocal imaging assays of live HepG2 and live HeLa Cells, live HEK293T stained with **1–5** was similar to that of concentration-dependent assays except that the incubation of live cells was carried out using **1–5** (10 μM) for 0.5 h.

The procedure of confocal imaging of live HepG2 cells co-stained with **3** and FITC-conjugated lectins was similar to that of concentration-dependent assays except that the

cells were incubated with **3** (10 μM) at 37 ºC for 0.5 h followed by incubation with paraformaldehyde fix solution for 15 mins and FITC-conjugated lectins (20 μg/mL) at 37 °C for 1 h. The red channel was collected with an excitation wavelength of 405 nm, and emission band of 700−800 nm. The green channel was collected with an excitation wavelength of 488 nm, and emission band of 500−550 nm.

The procedure of confocal imaging of live HepG2 cells co-stained with **3** and membrane-tracker was similar to that of concentration-dependent assays except that the cells were incubated with **3** at 37 ºC for 0.5 h followed by incubation of membrane tracker (5 μg/mL) at 37 °C for 10 min. The red channel was collected with an excitation wavelength of 405 nm, and emission band of 700−800 nm. The green channel was collected with an excitation wavelength of 635 nm, and emission band of 650−670 nm.

The procedure of confocal imaging of live HepG2 stained with **3** after treatment of neuraminidase was similar to that of concentration-dependent assays except that a dish of HepG2 cells was treated with the neuraminidase (an enzyme that can remove sialic acids) for 1 h at 37 ºC. Another dish of HepG2 cells was used a control group without any pretreatment. Then two dishes of cells were incubated with **3** at 37 ºC for 0.5 h.

MTT Cell Viability Assay

HepG2 cells were adhered onto a 96-well plate at around 10,000 cells per well respectively and were cultured with the corresponding growth media (100 μL) in a humidified incubator at 37 ºC for 24 h where the carbon dioxide level was kept constant at 5%. Different amounts of 1 (0, 0.78, 1.56, 3.13, 6.25, 12.5, 25, 50, 100 μM) in the corresponding growth media were applied and the cells were incubated at 37 ºC for 24 h. Wells containing cells without **3** were used as controls. Subsequently, 10 μL of MTT solution in PBS buffer (5 mg mL^{-1}) was added to each well and the plate was incubated at 37 ºC for 3 h. The solution was removed and the precipitated formazan was dissolved in DMSO (200 μL). After solubilization, the absorbance of formazan at 570 nm was measured with a microplate absorbance reader. The cell viability was expressed as a percentage ratio of the absorbance of the cells treated with **3** to that of the controls.

Computational Studies

All calculations were performed using the Gaussian16 suite of programs[3] on research computing facilities offered by Information Technology Service at the University of Hong Kong. The ground-state structures were optimized at PBE0 (Perdew-Burke-Ernzerhof parameter-free hybrid functional)[4,5] level in conjunction with the D3 version of Grimme's empirical dispersion with Becke-Johnson dampling (GD3BJ). The bulk solvent effect of the water environment was simulated with the SMD (solvation model based on density)[6] model to mimic the binding process in aqueous solution. Vibrational frequencies were calculated to confirm that optimized structures are true a minim on the potential energy surface, as no imaginary frequencies were observed (NIMAG = 0). Platinum centers were described using the Stuttgart (SDD) effective core potentials (ECP) and the associated basis set,[7] with two additional f-type polarization functions (ζ = 0.70 and 0.14),[8] whereas all remaining atoms were described using the 6-31G(d, p) basis set.[9–12] All DFT calculations were performed with a pruned (99,590) grid for numerical integration. Non-covalent interactions (NCI) between platinum(II) complexes and sialic acid were calculated with NCIPLOT,[13–15] which makes use of the electron density and its gradient at critical points between molecules, and the resulting isosurfaces were visualized using VMD (version 1.9.3) package.[16]

Calculation of binding energy

On the basis of the optimized geometries, electronic energies of the platinum(II) complexes, sialic acid (SA) and the ensemble of SA–platinum(II) complexes at the complete basis set (CBS) limit were extrapolated using correlation-consistent basis sets.[17] The basis set combination cc-pV*n*Z-PP (and the associated ECP) for platinum and cc-pV*n*Z[17] for the remaining atoms is abbreviated as V*n*Z (*n* = D, T, Q), corresponding to double-zeta, triple-zeta and quadruple-zeta respectively. Binding energies (ΔE_{bind}) were calculated by subtracting the electronic energy of the ensemble complexes from the sum of individual platinum(II) complexes and SA. The extrapolated CBS limit can minimize the basis set superposition error (BSSE), leading

to accurate ΔE_{bind} at PBE0-D3BJ levels of theory.[18]

The three-point exponential function scheme[19] was used to extrapolate the CBS limit,

$$E(X) = E_{\infty} + Be^{-aX}$$

where $X = 2$, 3, and 4 for VDZ, VTZ, and VQZ, respectively. E_{∞} is the electronic energy at the CBS limit, and B and a are fitting parameters.

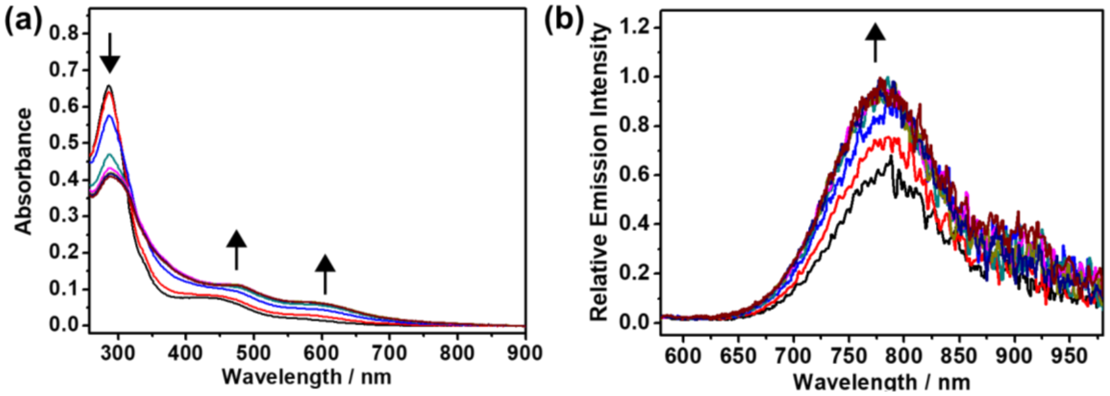

Figure S17. (a) Electronic absorption spectra and (b) emission spectra of **1** (30 μM) upon addition of polySia (0–2 equiv) in Tris–HCl buffer (10 mM Tris, 10 mM NaCl, pH = 8.0, containing 1% DMSO).

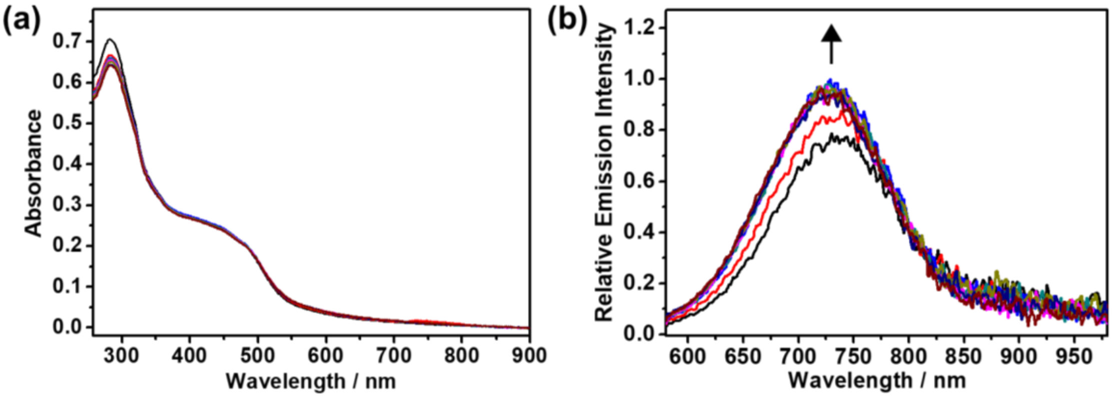

Figure S18. (a) Electronic absorption spectra and (b) emission spectra of **2** (30 μM) upon addition of polySia (0–2 equiv) in Tris–HCl buffer (10 mM Tris, 10 mM NaCl, pH = 8.0, containing 1% DMSO).

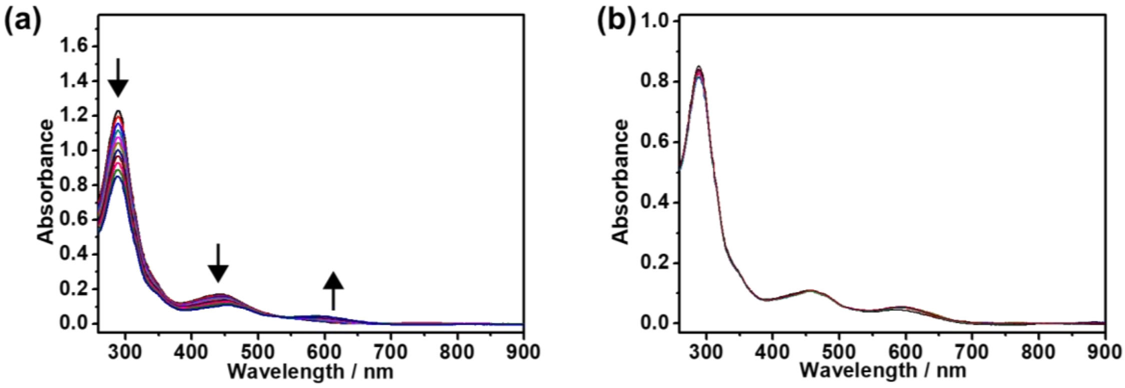

Figure S19. Electronic absorption spectra of **3** (30 μM) upon addition of (a) 0–2 equiv, (b) 2–3.8 equiv of polySia in Tris–HCl buffer (10 mM Tris, 10 mM NaCl, pH = 8.0)

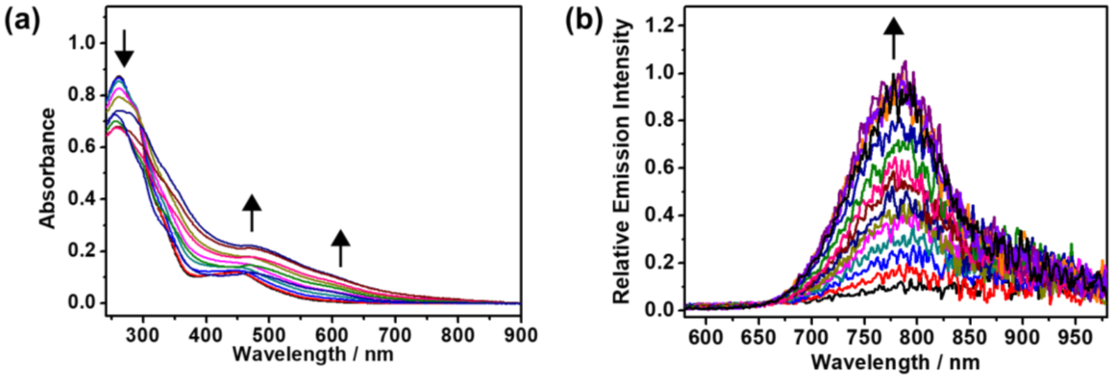

Figure S20. (a) Electronic absorption spectra and (b) emission spectra of **4** (30 μM) upon addition of polySia (0–3 equiv) in Tris–HCl buffer (10 mM Tris, 10 mM NaCl, pH = 8.0).

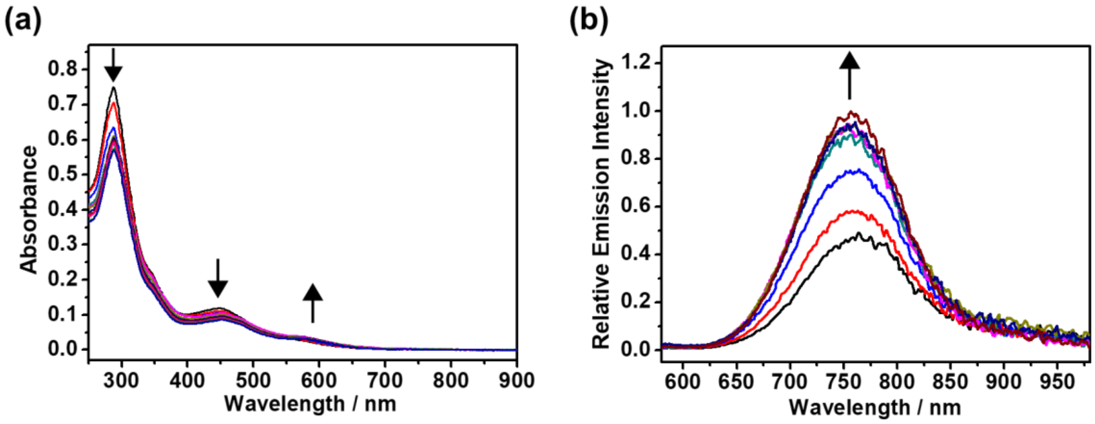

Figure S21. (a) Electronic absorption spectra and (b) emission spectra of **5** (30 μM) upon addition of polySia (0–2 equiv) in Tris–HCl buffer (10 mM Tris, 10 mM NaCl, pH = 8.0).

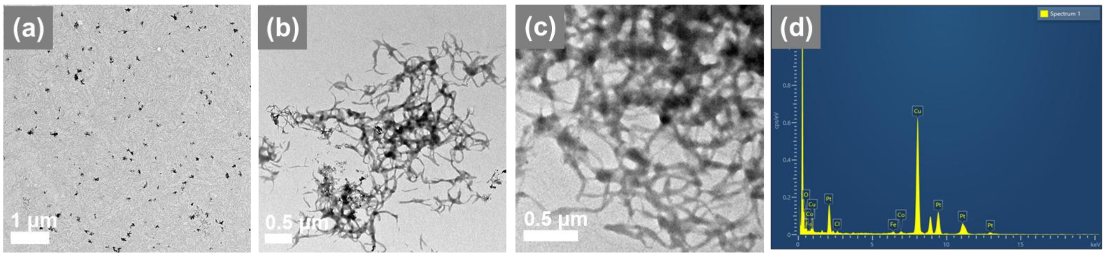

Figure S22. TEM images of (a) **1** (30 µM) and (b) **1** (30 µM) upon addition of polySia (30 µM) in an aqueous solution. (c) STEM image and (d) EDX spectrum of a solution containing **1** (30 µM) and polySia (30 µM) in H_2O (1% DMSO).

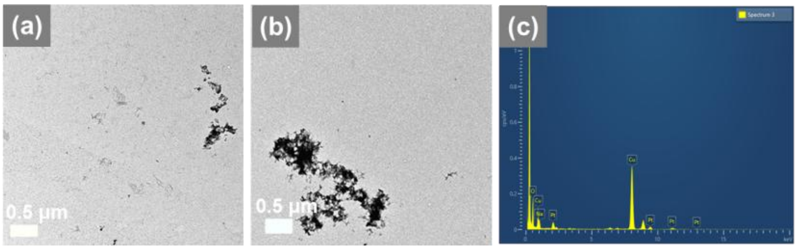

Figure S23. TEM images of (a) **2** (30 µM) and (b) **2** (30 µM) upon addition of polySia (30 µM) in an aqueous solution. (c) EDX spectrum of a solution containing **2** (30 µM) and polySia (30 µM) in H_2O (1% DMSO).

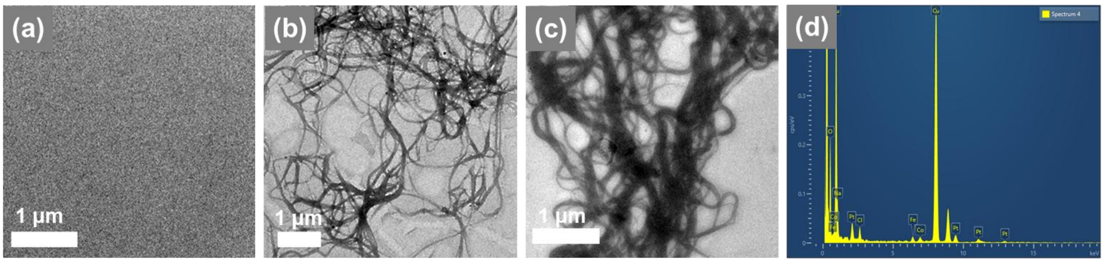

Figure S24. TEM images of (a) **3** (30 µM) and (b) **3** (30 µM) upon addition of polySia (30 µM) in an aqueous solution. (c) STEM image and (d) EDX spectrum of a solution containing **3** (30 µM) and polySia (30 µM) in H_2O.

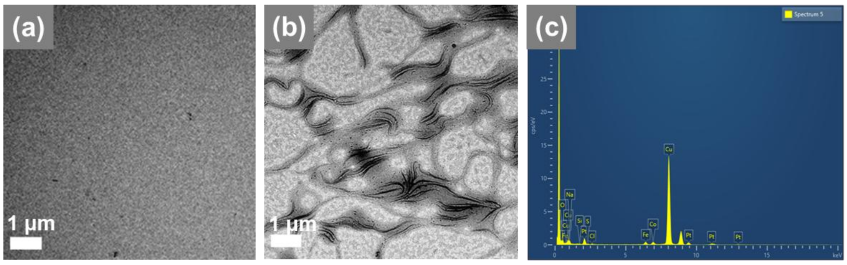

Figure S25. TEM images of (a) **4** (30 μM) and (b) **4** (30 μM) upon addition of polySia (30 μM) in an aqueous solution. (c) EDX spectrum of a solution containing **4** (30 μM) and polySia (30 μM) in H_2O.

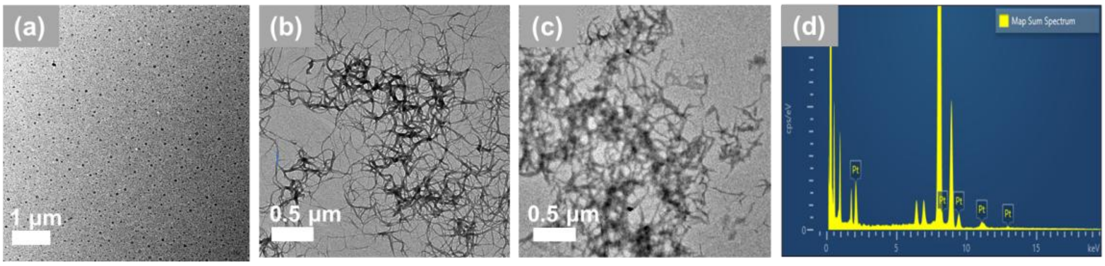

Figure S26. TEM images of (a) **5** (30 μM) and (b) **5** (30 μM) upon addition of polySia (30 μM) in an aqueous solution. (c) STEM image and (d) EDX spectrum of a solution containing **5** (30 μM) and polySia (30 μM) in H_2O.

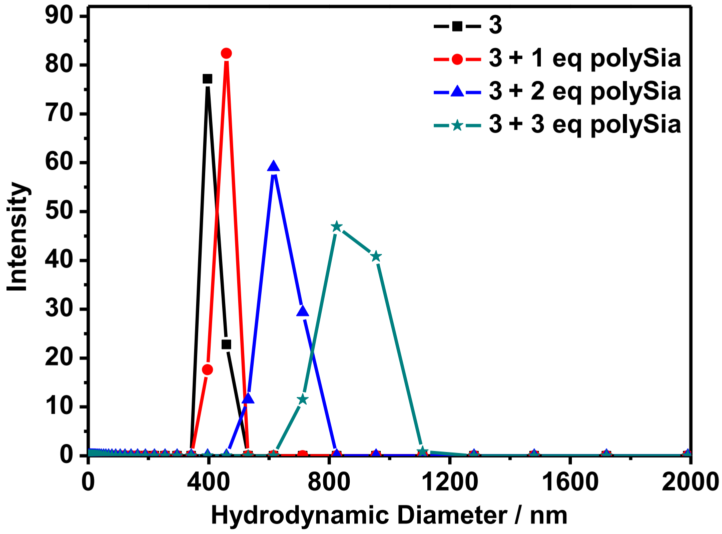

Figure S27. DLS data of **3** (30 μM) upon addition of polySia (0–3 equiv) in the Tris–HCl buffer (10 mM Tris, 10 mM NaCl, pH = 8.0).

Table S1. Hydrodynamic diameter of **3** in the presence of 1–3 equiv of polySia

Sample	Hydrodynamic Diameter / nm
**3**	410
**3** + 1 eq polySia	448
**3** + 2 eq polySia	634
**3** + 3 eq polySia	868

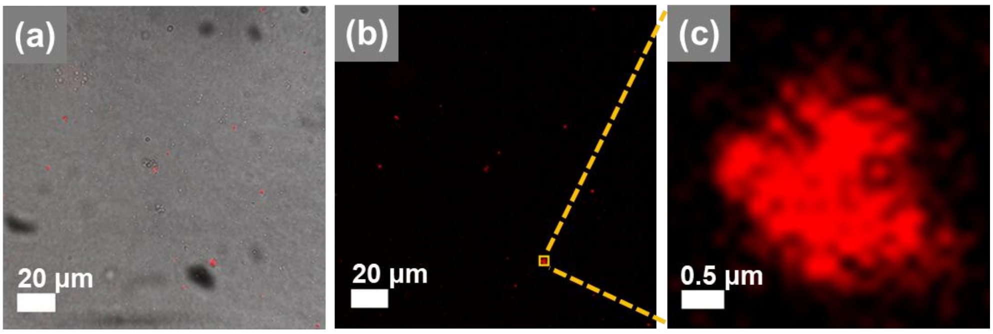

Figure S28. (a) The merged image of bright-field and luminescence image (650–750 nm), (b) luminescence image, and (c) 35× magnified luminescence image of **1** upon addition of 3 equiv of polySia in the Tris–HCl buffer (10 mM Tris, 10 mM NaCl, pH = 8.0).

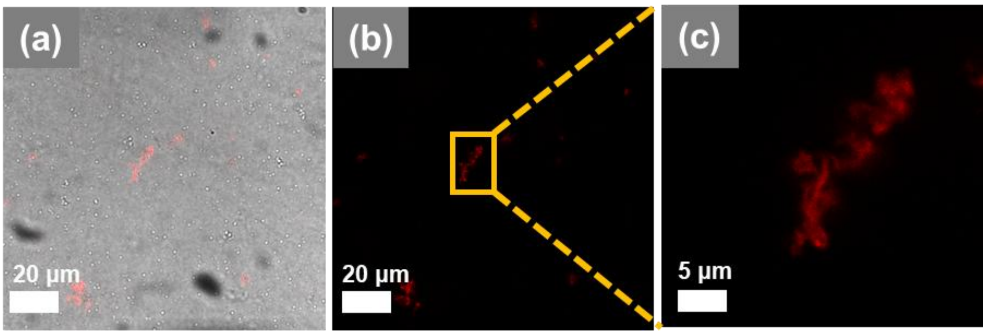

Figure S29. (a) The merged image of bright-field and luminescence image (650–750 nm), (b) luminescence image, and (c) 5× magnified luminescence image of **3** upon addition of 3 equiv of polySia in the Tris–HCl buffer (10 mM Tris, 10 mM NaCl, pH = 8.0).

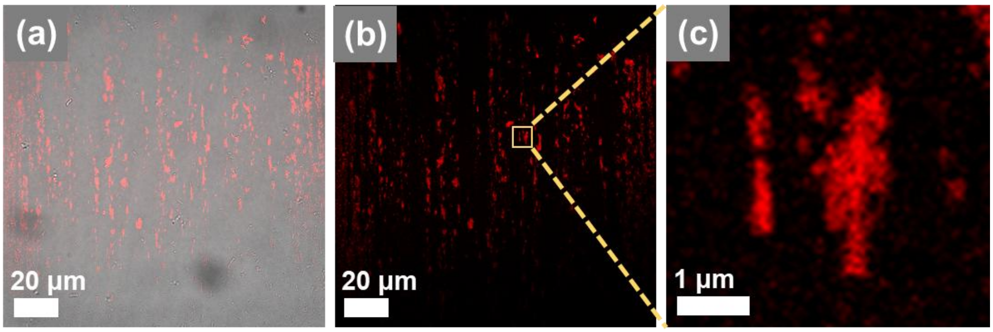

Figure S30. (a) The merged image of bright-field and luminescence image (650–750 nm), (b) luminescence image, and (c) 15× magnified luminescence image of **4** upon addition of 3 equiv of polySia in the Tris–HCl buffer (10 mM Tris, 10 mM NaCl, pH = 8.0).

Table S2. *d*-Spacing values of **1–4** in the presence of 1 equiv of polySia

Sample	*d*-spacing
**1**	3.259 Å
**3**	3.229 Å
**4**	3.488 Å

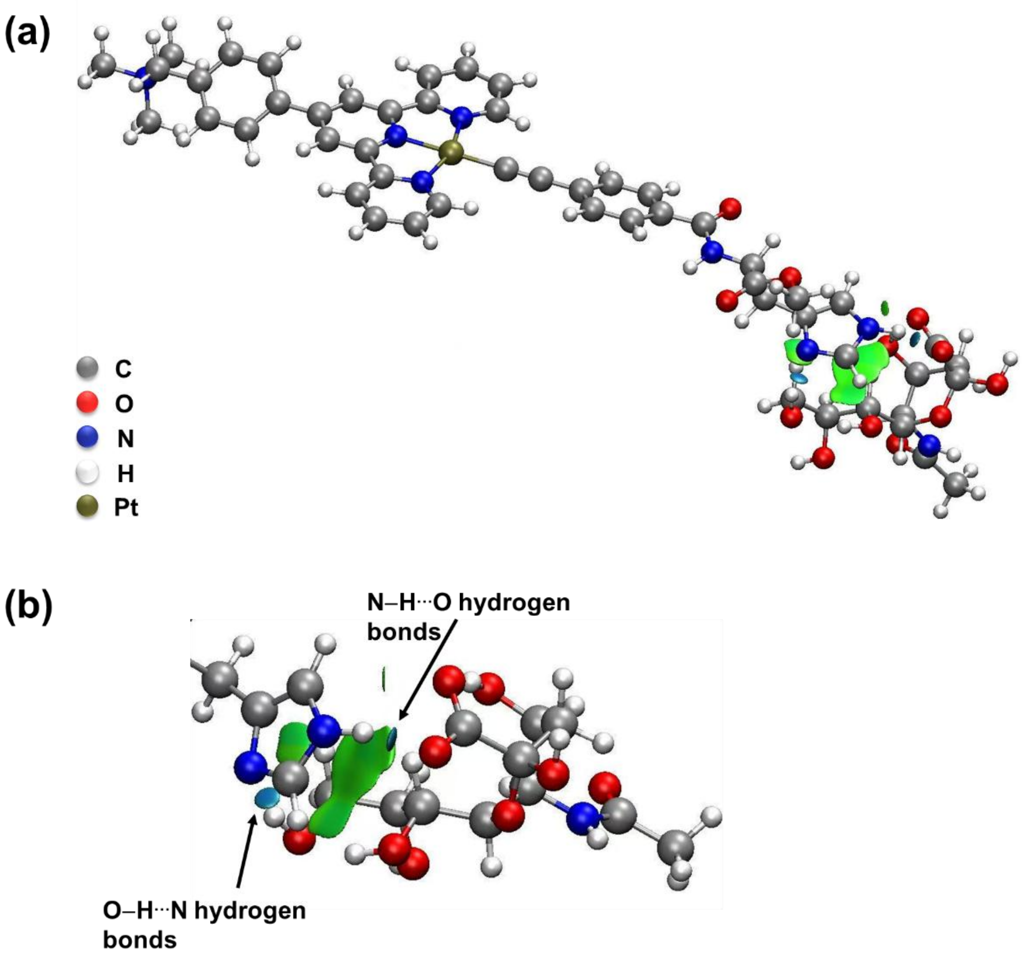

Figure S31. (a) Isosurfaces of noncovalent interactions (NCI) for the system comprising **3** and sialic acid. (b) Magnified images showing the isosurfaces of NCI. Two blue regions indicated by black arrows represent strong attraction resulting from hydrogen bonds while green region represents weak interaction.

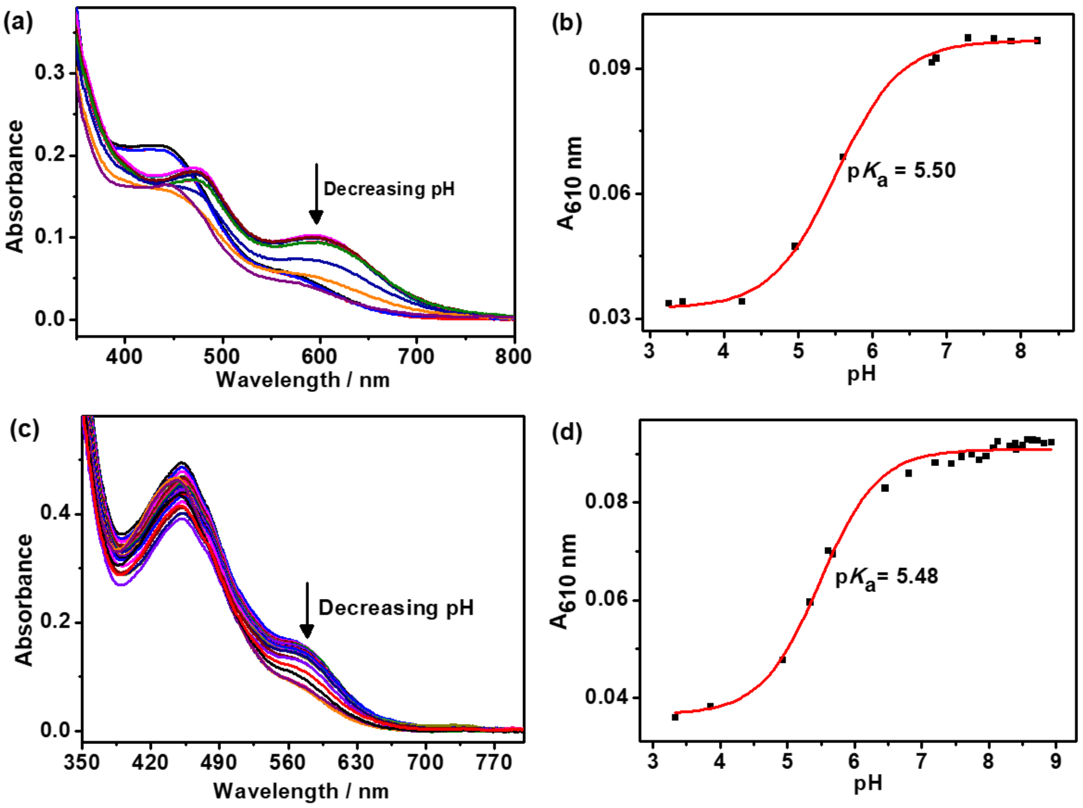

Figure S32. Electronic absorption spectra of (a) **1** (100 μM) and (c) **3** (100 μM) in aqueous buffer upon decreasing pH (a) from 8.22 to 3.26 and (c) from 8.93 to 3.33. A plot of absorbance at 610 nm of (b) **1** and (d) **3** versus pH.

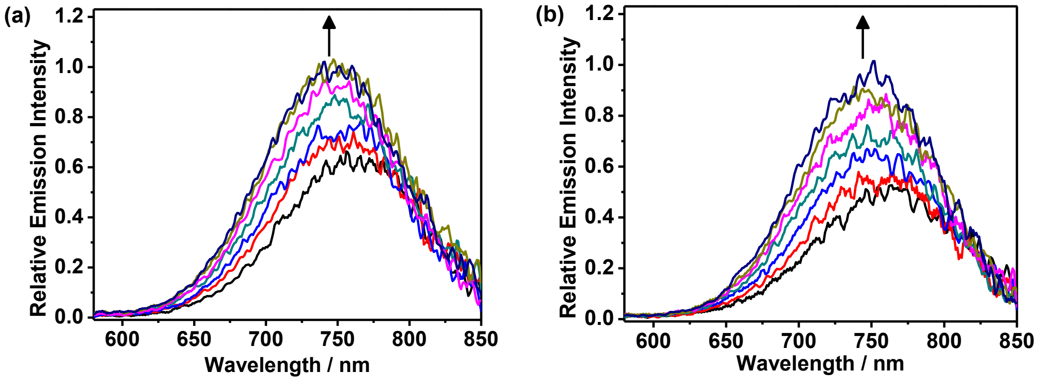

Figure S33. Emission spectra of **3** (30 μM) upon the addition of polySia (0–36 μM) in Tris–HCl buffer (10 mM Tris, 10 mM NaCl) with pH of (a) 6.2 and (b) 5.5. Excitation wavelength was at 530 nm.

Table S3. Binding constants (log *K*), dissociation constants (K_d) and Hill coefficients (*n*) of **3** determined by Hill plots in Tris–HCl buffer with different pH (10 mM Tris, 10 mM NaCl)

pH	log *K*	K_d / M	*n*
5.5	5.71	1.95×10^{-5}	1.28
6.2	6.18	6.55×10^{-7}	1.41
8.0	8.02	9.52×10^{-9}	1.75

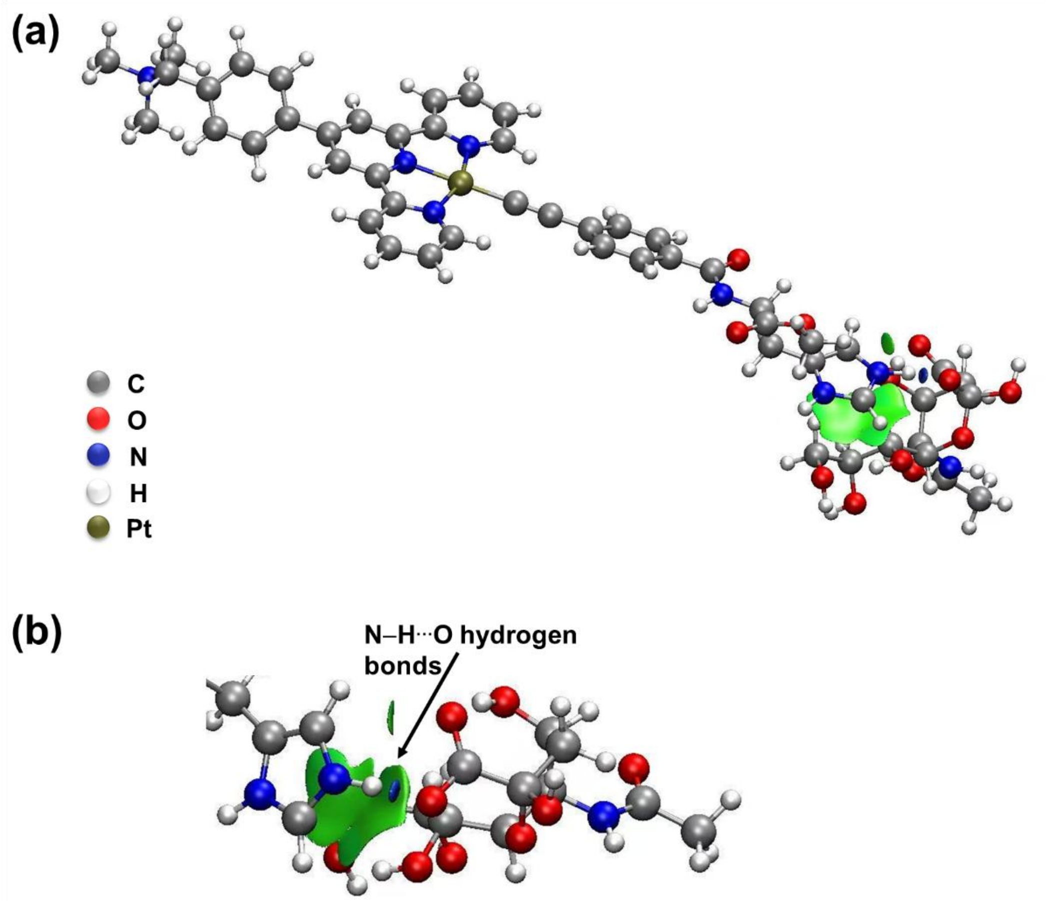

Figure S34. (a) Isosurfaces of noncovalent interactions (NCI) for the system comprising protonation form of **3** (with imidazolium moiety) and sialic acid. (b) Magnified images showing the isosurfaces of NCI. Blue region indicated by black arrows represents strong attraction while green region represents weak interaction.

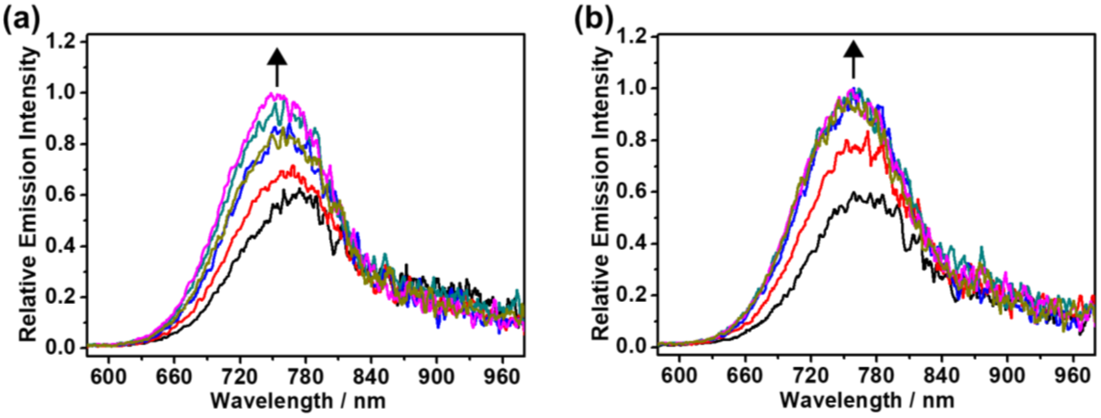

Figure S35. Emission spectra of **5** (30 μM) upon addition of polySia in Tris–HCl buffer (10 mM Tris, 10 mM NaCl) with (a) pH 8.5 and (b) pH 9.0.

Table S4. Binding constants (log *K*), dissociation constants (K_d) and Hill coefficients (*n*) of **5** determined by Hill plots in Tris–HCl buffer with different pH (10 mM Tris, 10 mM NaCl)

pH	log *K*	K_d / M	*n*
8.0	6.85	1.40×10^{-7}	1.47
8.5	5.65	2.24×10^{-6}	1.25
9.0	4.80	1.58×10^{-5}	1.04

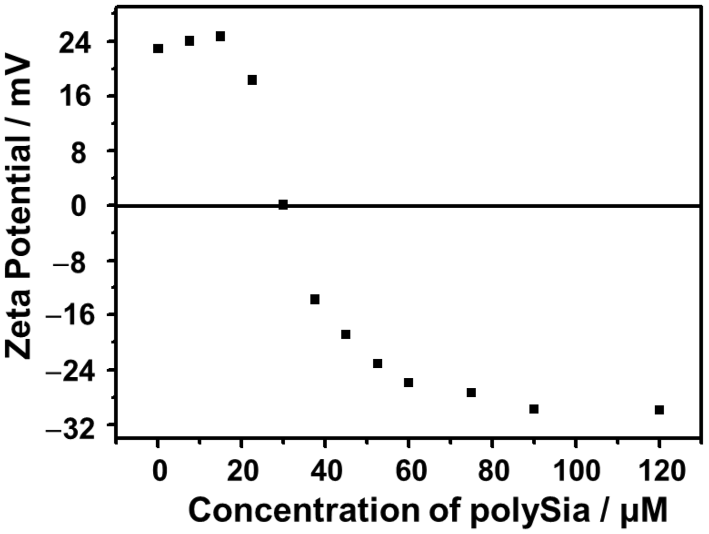

Figure S36. Zeta potential of **3** (30 μM) upon addition of polySia (0–4 equiv) in Tris–HCl buffer (10 mM Tris, 10 mM NaCl, pH = 8.0).

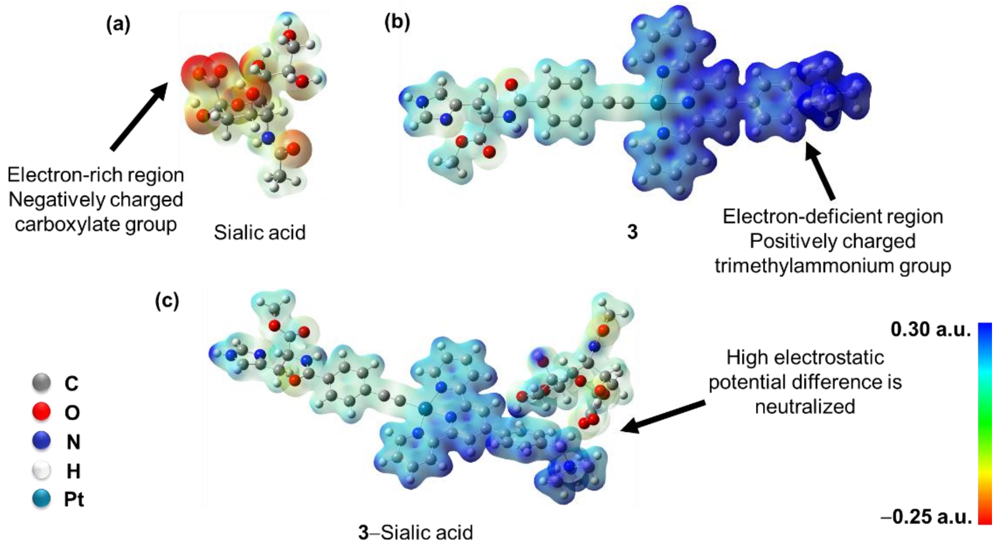

Figure S37. Electrostatic potential surfaces (isovalue = 0.02) of (a) sialic acid, (b) **3**, and (c) the ensemble of **3**–sialic acid. Red and blue areas correspond to negative and positive charge, respectively.

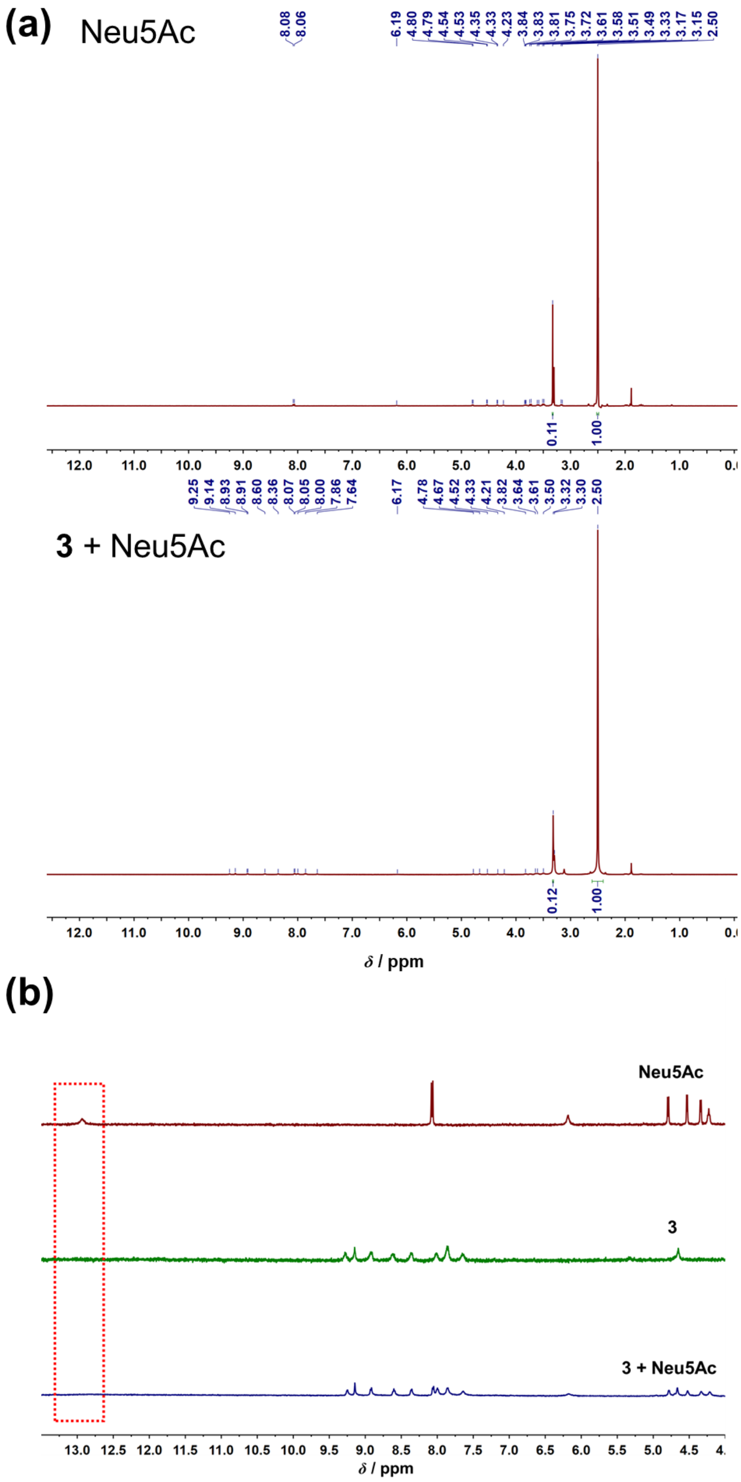

Figure S38. (a) ^{1}H NMR spectra of Neu5Ac (200 μM), and the mixture of **3** (200 μM) and Neu5Ac (200 μM) in DMSO-d_6 at 298 K. (b) Partial ^{1}H NMR spectra of Neu5Ac (200 μM), **3** (200 μM), and their mixture in DMSO-d_6 at 298 K.

Table S5. The energies (E) (hartree) of platinum(II) complex, sialic acid (SA) and the ensemble of platinum(II) complexes–SA and the binding energies (ΔE_{bind}) between histidine and NMe_3 moiety of **3**, NMe_2 moiety of **5** and SA calculated using VDZ, VTZ and VQZ basis sets, followed by the complete basis set (CBS) extrapolation. All energy values are given in hartrees.

	VDZ	VTZ	VQZ	CBS
E(**3**(His)–SA)	–3472.705577	–3473.599692	–3473.850355	–3473.948004
E(**3**(NMe_3)–SA)	–3472.712281	–3473.605541	–3473.855829	–3473.953258
E(**5**(NMe_2)–SA)	–3432.974808	–3433.859241	–3434.108222	–3434.205777
E(**3**)	–2311.840925	–2312.395013	–2312.552015	–2312.614092
E(**5**)	–2272.114484	–2272.656656	–2272.811574	–2272.873548
E(SA)	–1160.842625	–1161.190525	–1161.287196	–1161.324393
ΔE_{bind}(**3**(His)–SA)	–0.02202654	–0.01415353	–0.01114451	–0.00951906
ΔE_{bind}(**3**(NMe_3)–SA)	–0.02873031	–0.02000241	–0.01661812	–0.01477313
ΔE_{bind}(**5**(NMe_2)–SA)	–0.01769777	–0.01206019	–0.00945265	–0.00783584

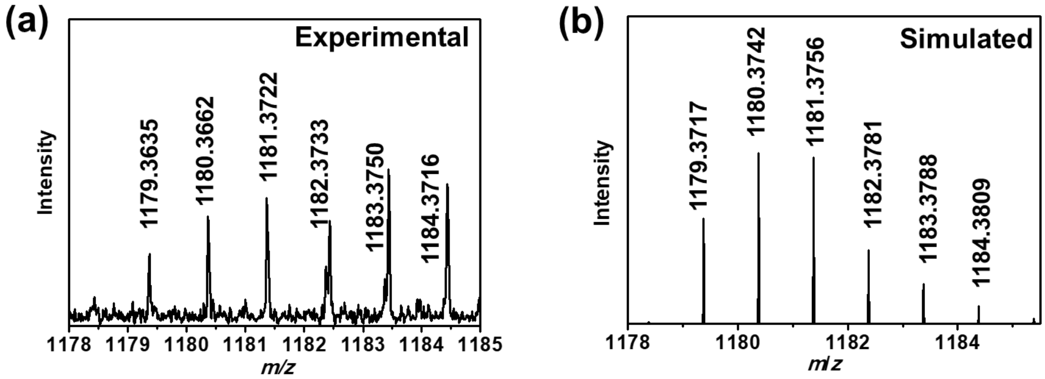

Figure S39. (a) Expanded ion cluster of high-resolution positive electrospray ionization (ESI) mass spectrum of the ensemble of **3** and Neu5Ac, and (b) the corresponding simulated isotope pattern of **3** and Neu5Ac.

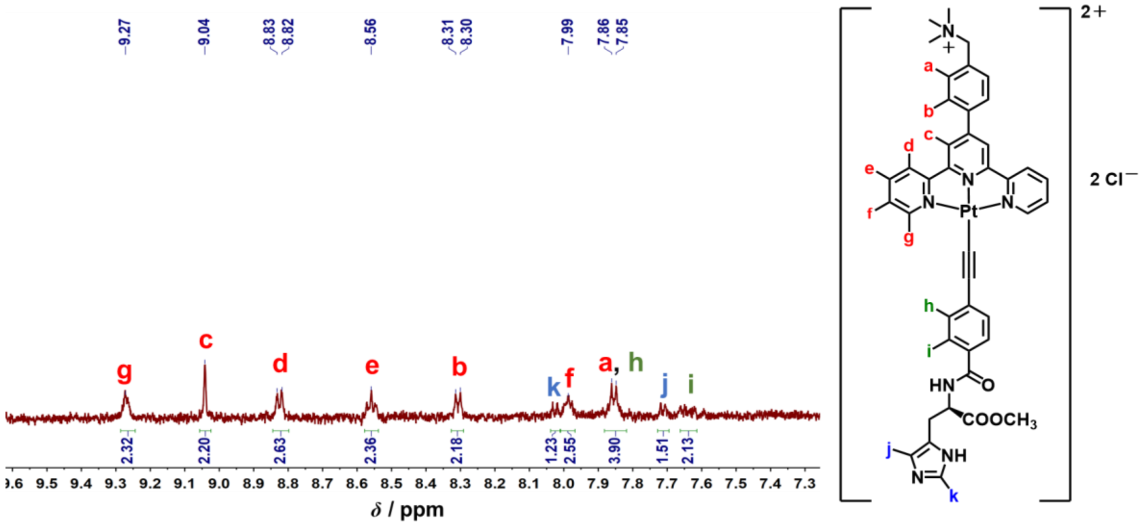

Figure S40. ^{1}H NMR spectrum of **3** in DMSO-d_6–D_2O (9:1, v/v) at 298K.

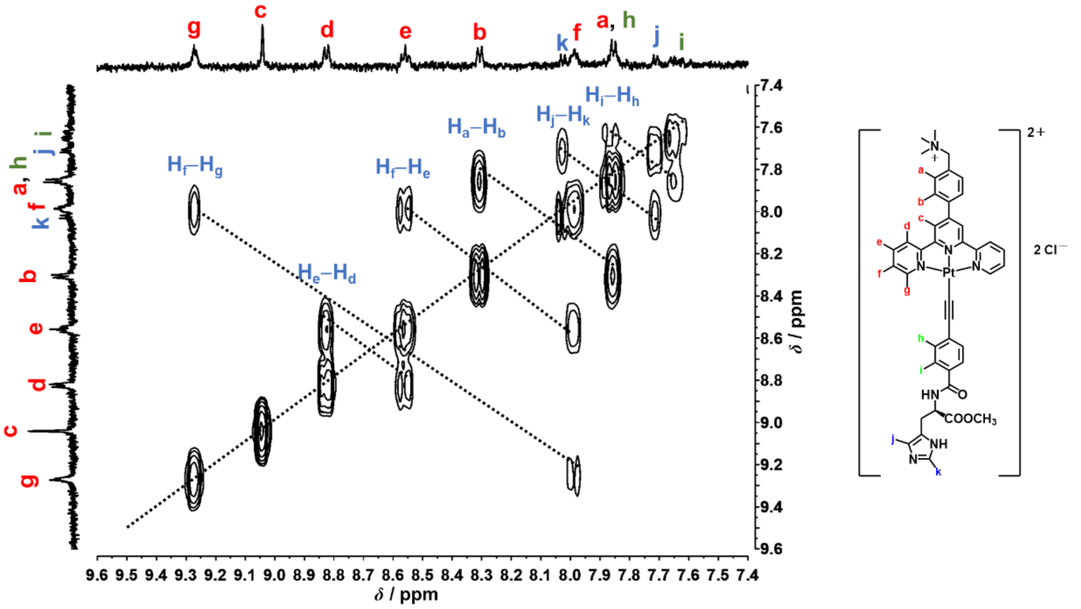

Figure S41. ^{1}H–^{1}H COSY NMR spectrum of **3** (200 μM) upon addition of polySia (200 μM) in DMSO-d_6–D_2O (9:1, v/v) at 298K. The signals between neighboring protons marked by text with blue color have been observed, including H_f and H_g, H_e and H_d, H_f and H_e, H_a and H_b, H_j and H_k, H_i and H_h.

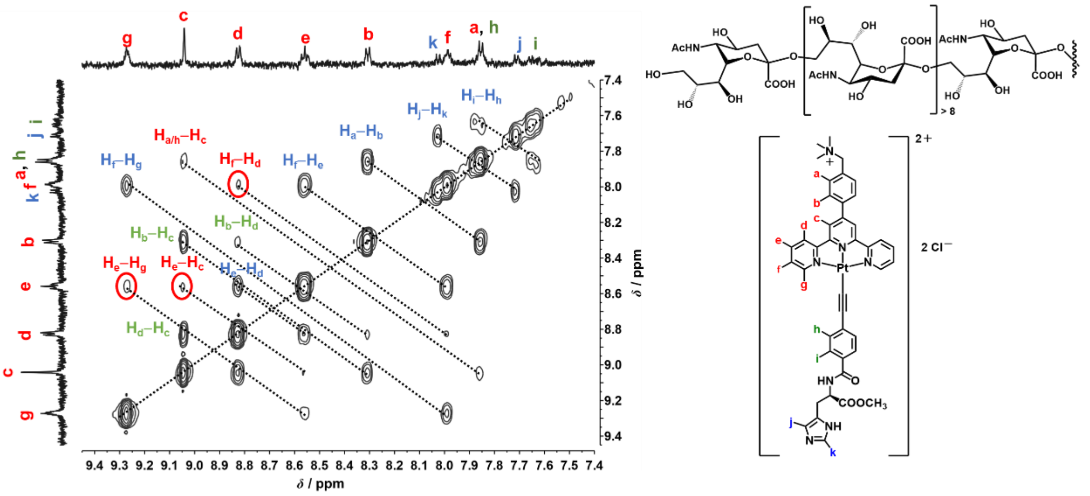

Figure S42. ^1H–^1H NOESY NMR spectrum of **3** (200 μM) upon addition of polySia (200 μM) in DMSO-d_6–D_2O (9:1, v/v) at 298K. The signals between neighboring protons marked by text with blue color have been observed, including H_f and H_g, H_e and H_d, H_f and H_e, H_a and H_b, H_j and H_k, H_i and H_h. The signals between protons in spatial proximity marked by text with green color have been observed, including H_b and H_c, H_d and H_c, H_b and H_d. Cross-peaks between terpyridine protons (H_e and H_g, H_e and H_c, H_f and H_d) with NOE interactions marked by red circles have been observed.

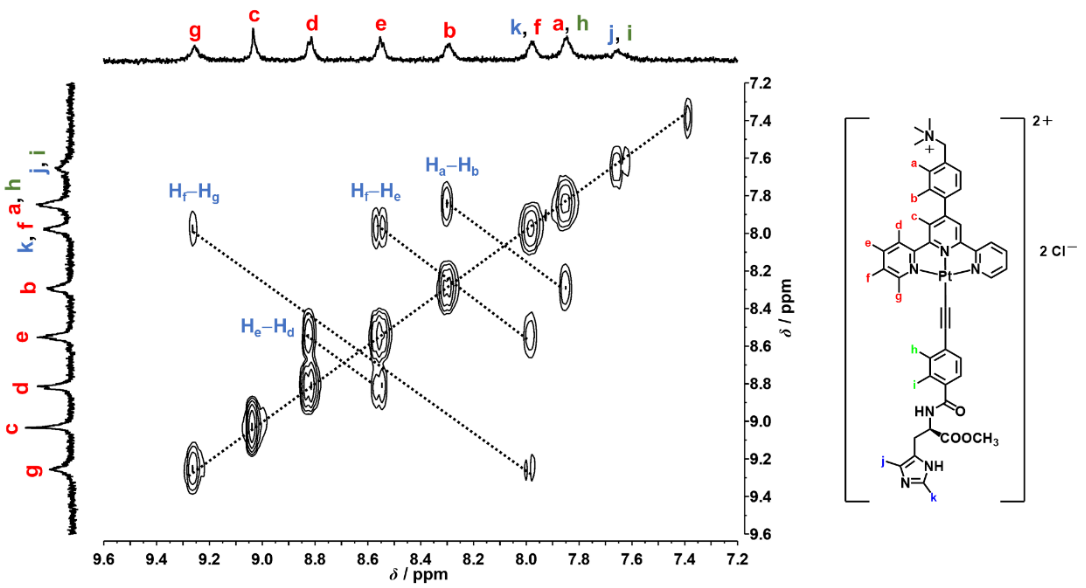

Figure S43. ^{1}H–^{1}H COSY NMR spectrum of **3** (200 μM) upon addition of Neu5Ac (200 μM) in DMSO-d_6–D_2O (9:1, v/v) at 298K. The signals between neighboring protons marked by text with blue color have been observed, including H_f and H_g, H_e and H_d, H_f and H_e, H_a and H_b.

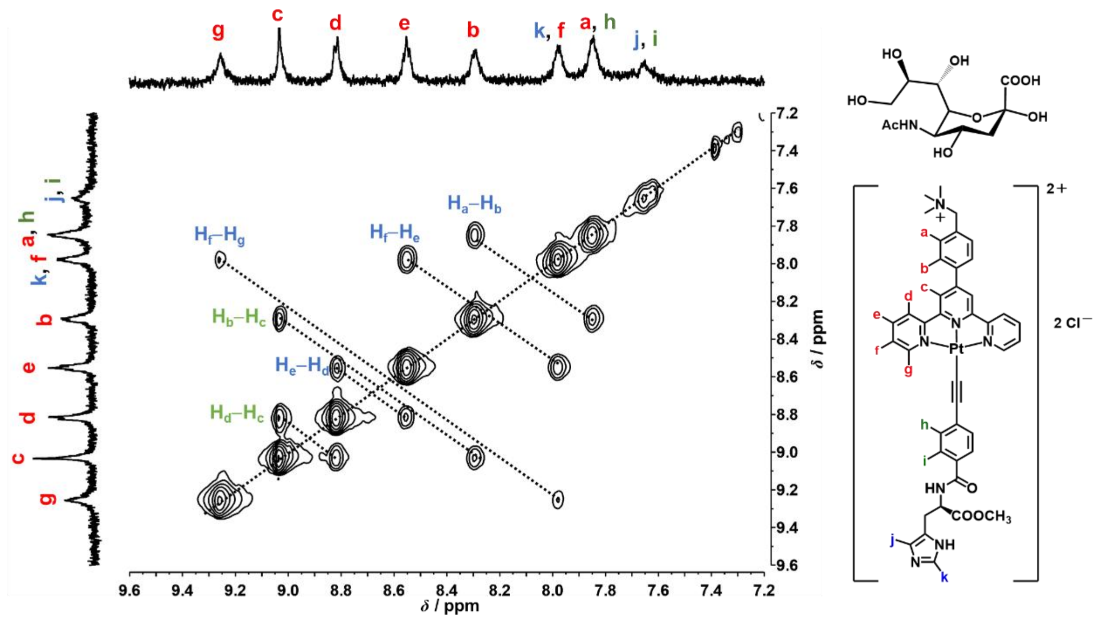

Figure S44. ^1H–^1H NOESY NMR spectrum of **3** (200 μM) upon addition of Neu5Ac (200 μM) in DMSO-d_6–D_2O (9:1, v/v) at 298K. The signals between neighboring protons marked by text with blue color have been observed, including H_f and H_g, H_e and H_d, H_f and H_e, H_a and H_b, matching with COSY NMR spectrum. The signals between protons in spatial proximity marked by text with green color have been observed, including H_b and H_c, H_d and H_c.

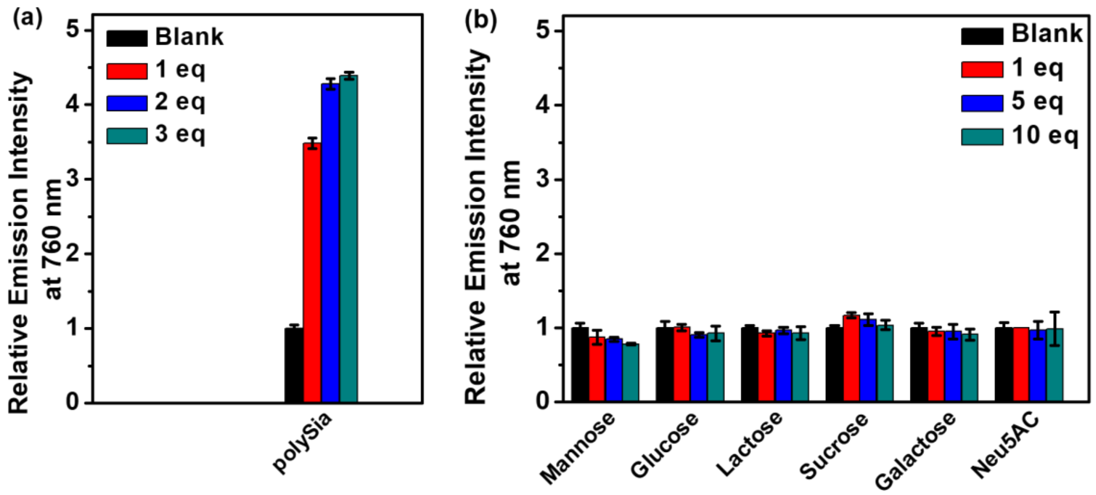

Figure S45. Relative emission intensity at 760 nm of **3** (30 μM) upon the addition of (a) polySia (30, 60, 90 μM) and (b) different monosaccharides (mannose, glucose, lactose, sucrose, galactose and Neu5Ac) (30, 150, 300 μM) in the Tris-HCl buffer (10 mM Tris, 10 mM NaCl, pH = 8.0).

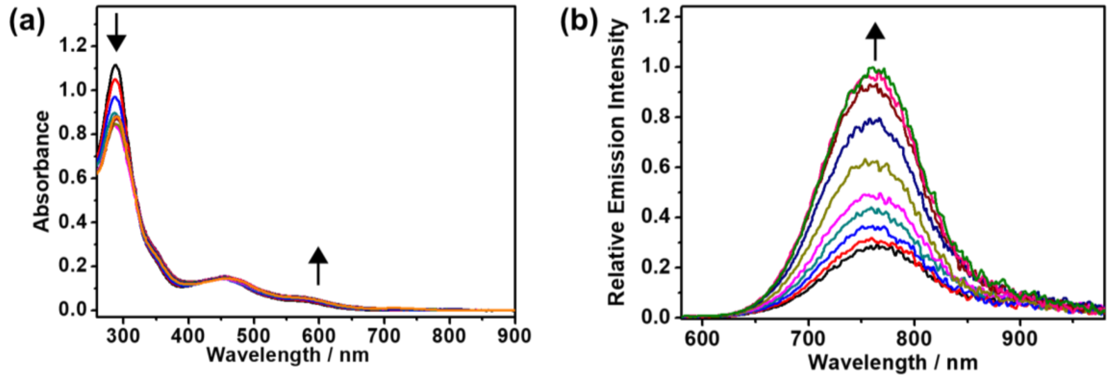

Figure S46. (a) Electronic absorption spectra and (b) corrected emission spectra of **3** (30 μM) upon addition of polySia (0–105 μM) in Tris–HCl buffer (10 mM Tris, 10 mM NaCl, pH = 7.4).

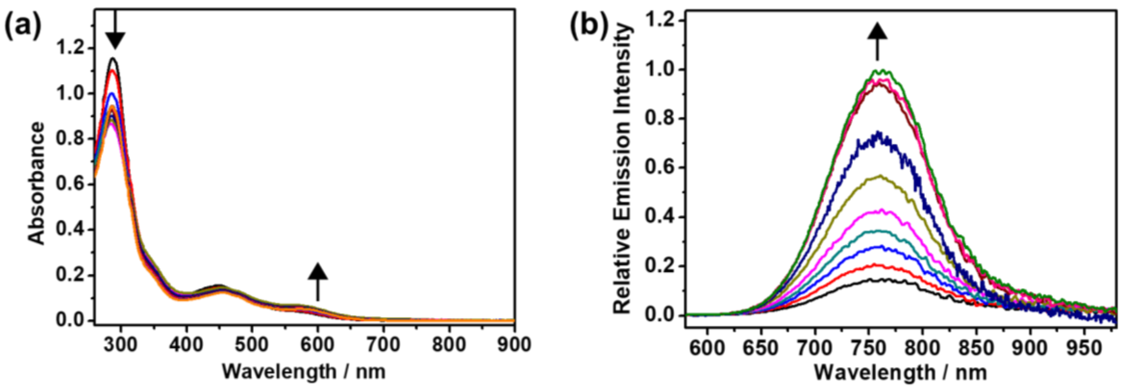

Figure S47. (a) Electronic absorption spectra and (b) corrected emission spectra of **3** (30 μM) upon addition of polySia (0–120 μM) in Tris–HCl buffer (10 mM Tris, 10 mM NaCl, pH = 9.0).

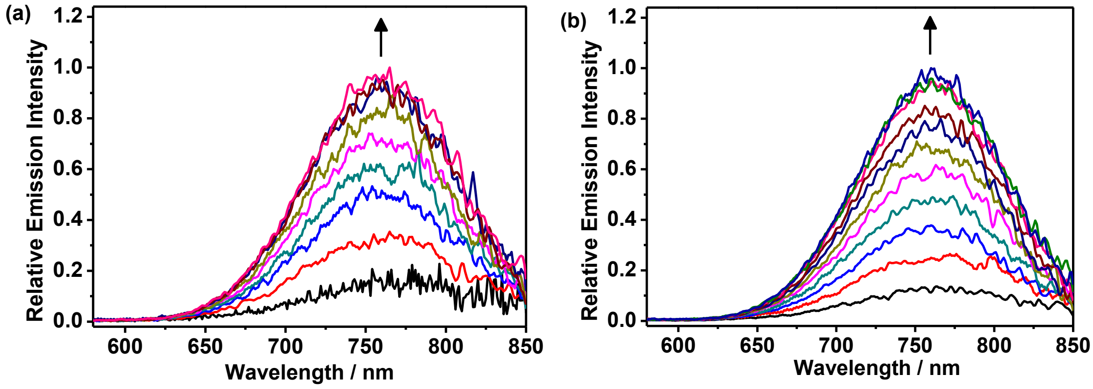

Figure S48. Emission spectra of **3** (30 μM) upon the addition of polySia in Tris–HCl buffer (10 mM Tris, 10 mM NaCl) with (a) pH 6.8 and (b) pH 7.2. Excitation wavelength was at 530 nm.

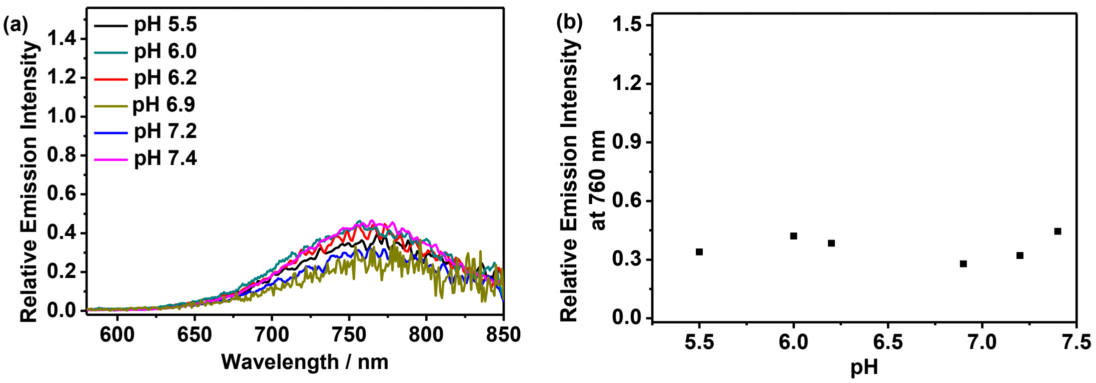

Figure S49. (a) Emission spectra of **3** (30 μM) in Tris–HCl buffer (10 mM Tris, 10 mM NaCl) at different pH (5.5, 6.0, 6.2, 6.9, 7.2, 7.4). (b) Plot of emission intensity at 760 nm of versus pH.

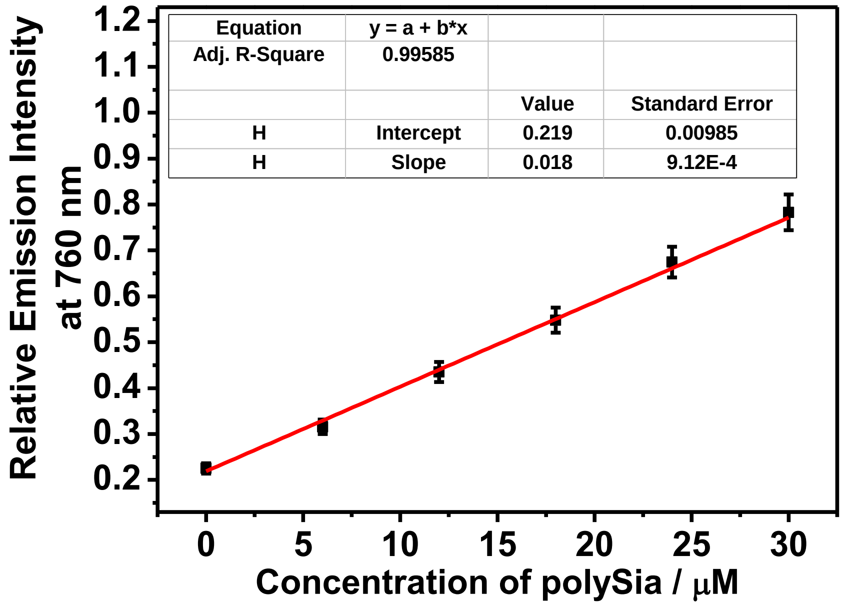

Figure S50. The relative emission intensity at 760 nm versus concentration of polySia in the range of 0–30 μM. The plot are obtained from emission spectra of **3** (30 μM) upon addition of polySia (0–3 equiv) in Tris–HCl buffer (10 mM Tris, 10 mM NaCl, pH = 8.0).

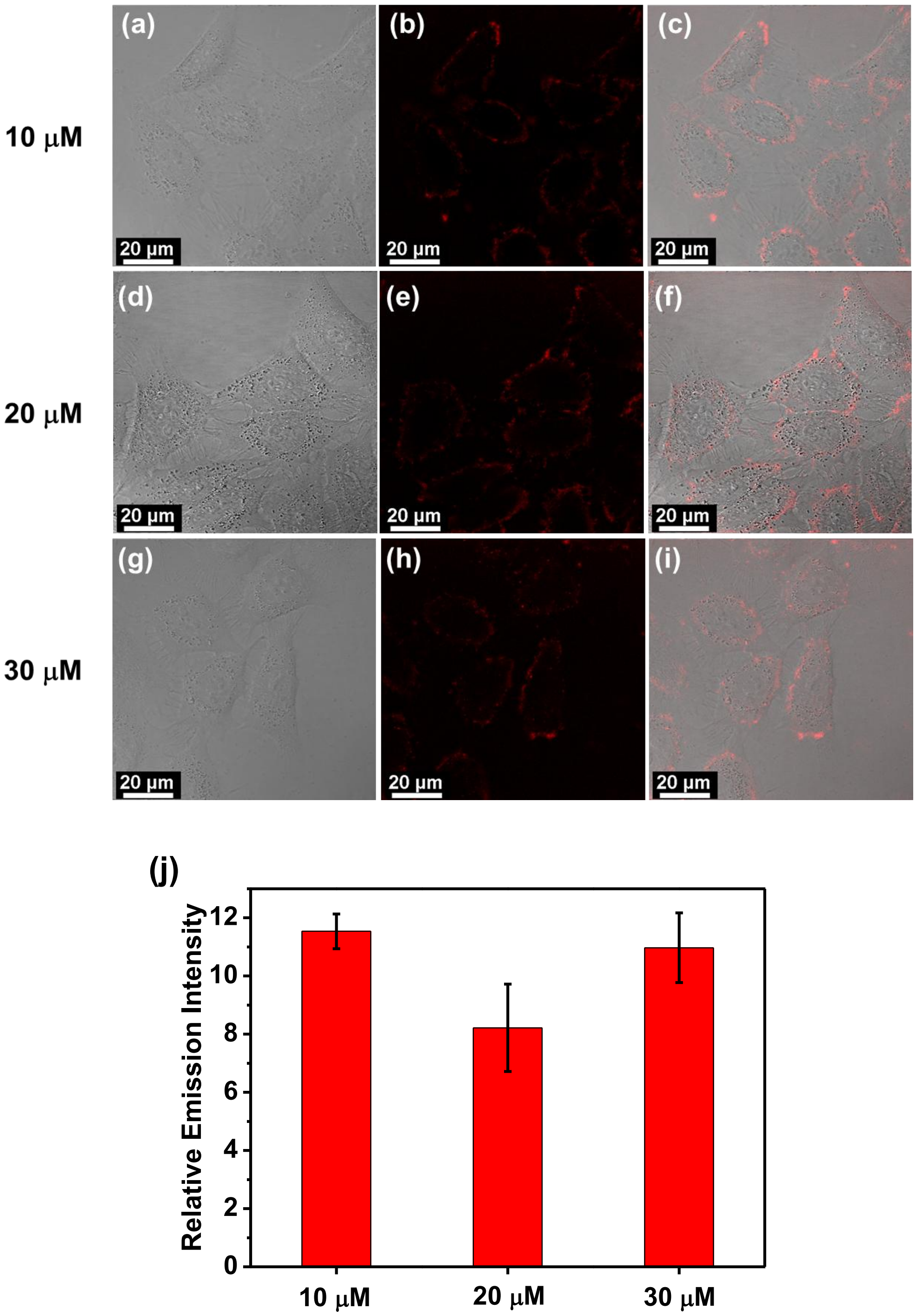

Figure S51. Confocal images of live HeLa cells stained with **3** of different concentrations of (a–c) 10 μM, (d–f) 20 μM, (g–i) 30 μM at 37 °C for 1 h. (a, e, g) Bright-field, (b, e, h) luminescence and (c, f, i) merged confocal images with bright-field and emission collected at 700–800 nm. (j) A bar graph comparing the relative emission intensity of live HeLa cells stained with **3** of the different concentrations.

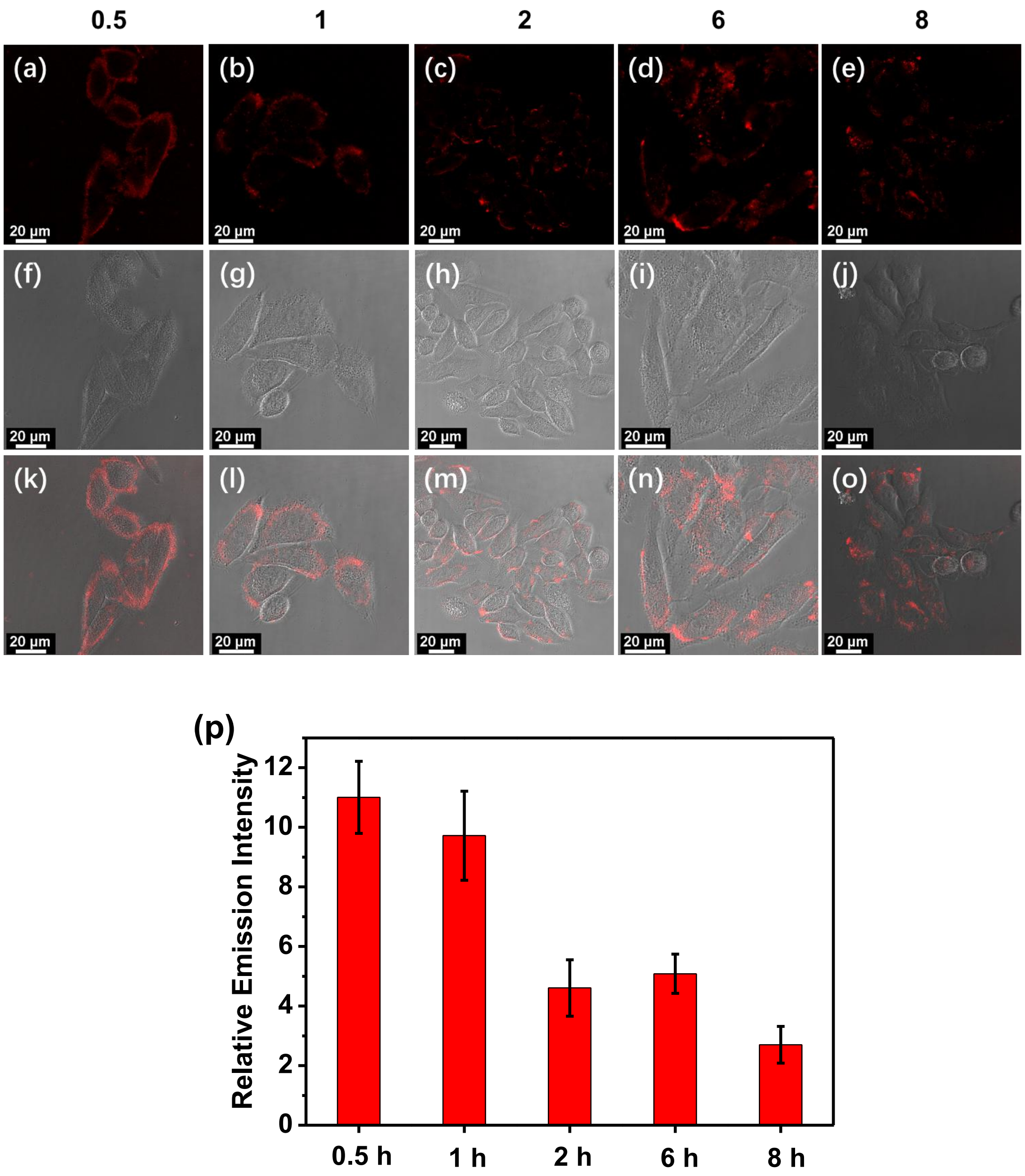

Figure S52. Confocal images of live HepG2 cells incubated with **3** (10 μM) at 37 °C for (a, f, k) 0.5 h, (b, g, l) 1 h, (c, h, m) 2 h, (d, i, n) 6 h, (e, j, o) 8 h. (a–e) Luminescence, (f–j) bright-field and (k-o) merged confocal images with bright-field and emission collected at 700–800 nm. (p) A bar graph comparing the relative emission intensity of live HepG2 cells stained with **3** for 0.5 h, 2 h, 6 h, and 8 h.

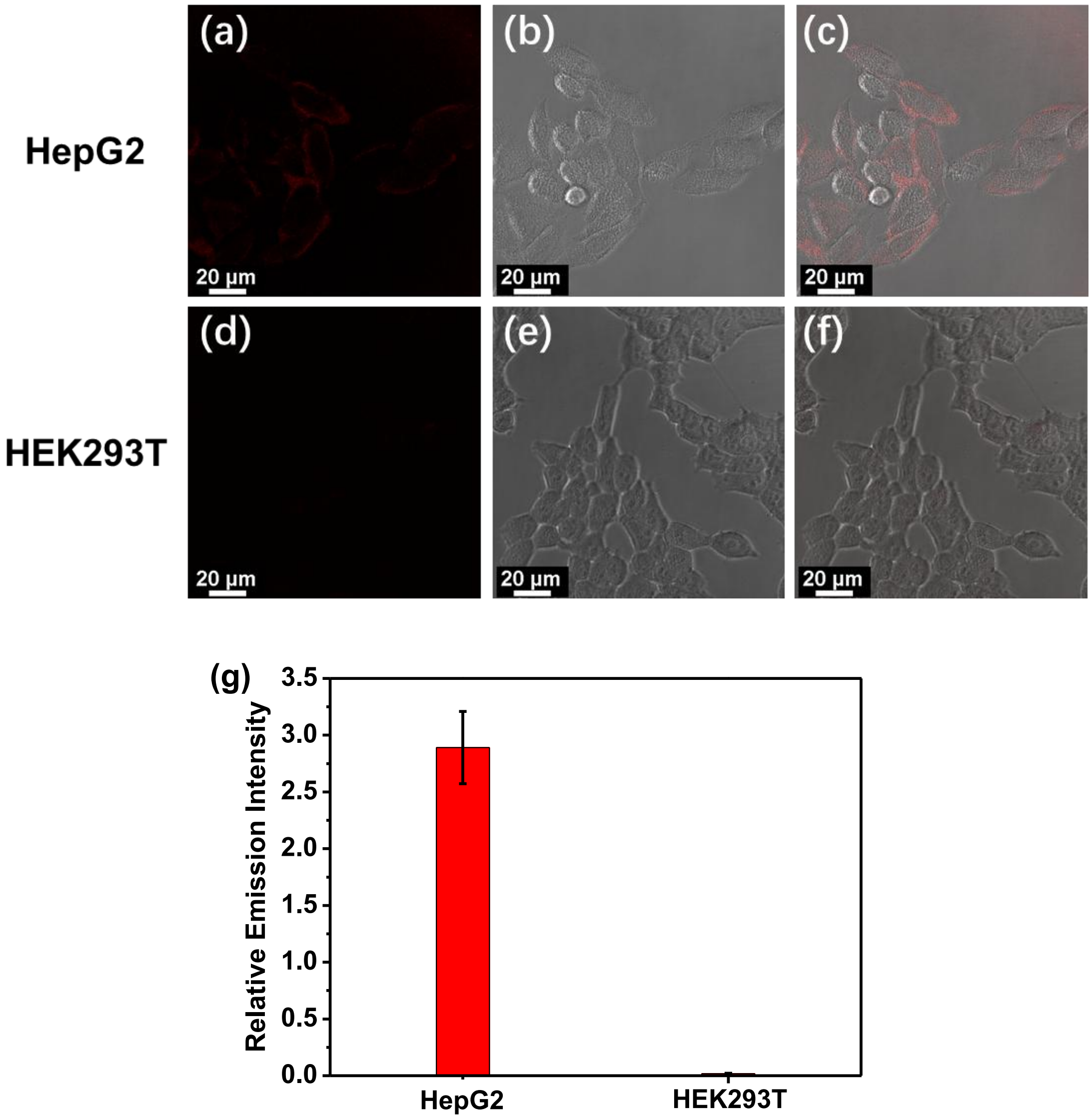

Figure S53. Confocal images of (a–c) live HepG2 and (d–f) live HEK293T cells stained with **4** (10 μM) at 37 °C for 30 mins. (a, d) Luminescence, (b, e) bright-field and (c, f) merged confocal images with bright-field and emission collected at 700–800 nm. (g) A bar graph comparing the relative emission intensity of the HepG2 and HEK293T cells stained with **4**.

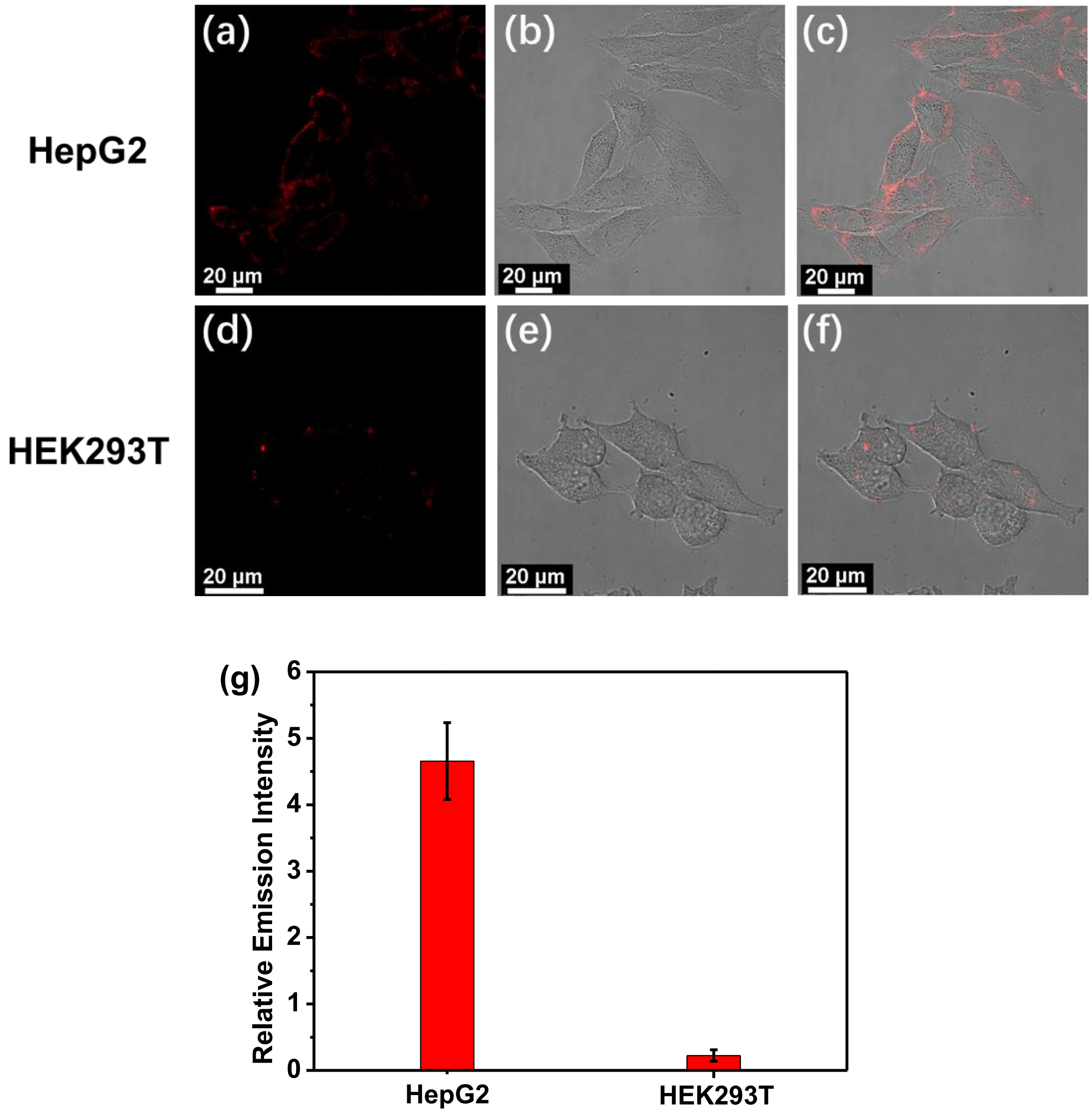

Figure S54. Confocal images of (a–c) live HepG2 and (d–f) live HEK293T cells stained with **5** (10 μM) at 37 °C for 30 mins. (a, d) Luminescence, (b, e) bright-field and (c, f) merged confocal images with bright-field and emission collected at 700–800 nm. (g) A bar graph comparing the relative emission intensity of HepG2 and HEK293T cells stained with **5**.

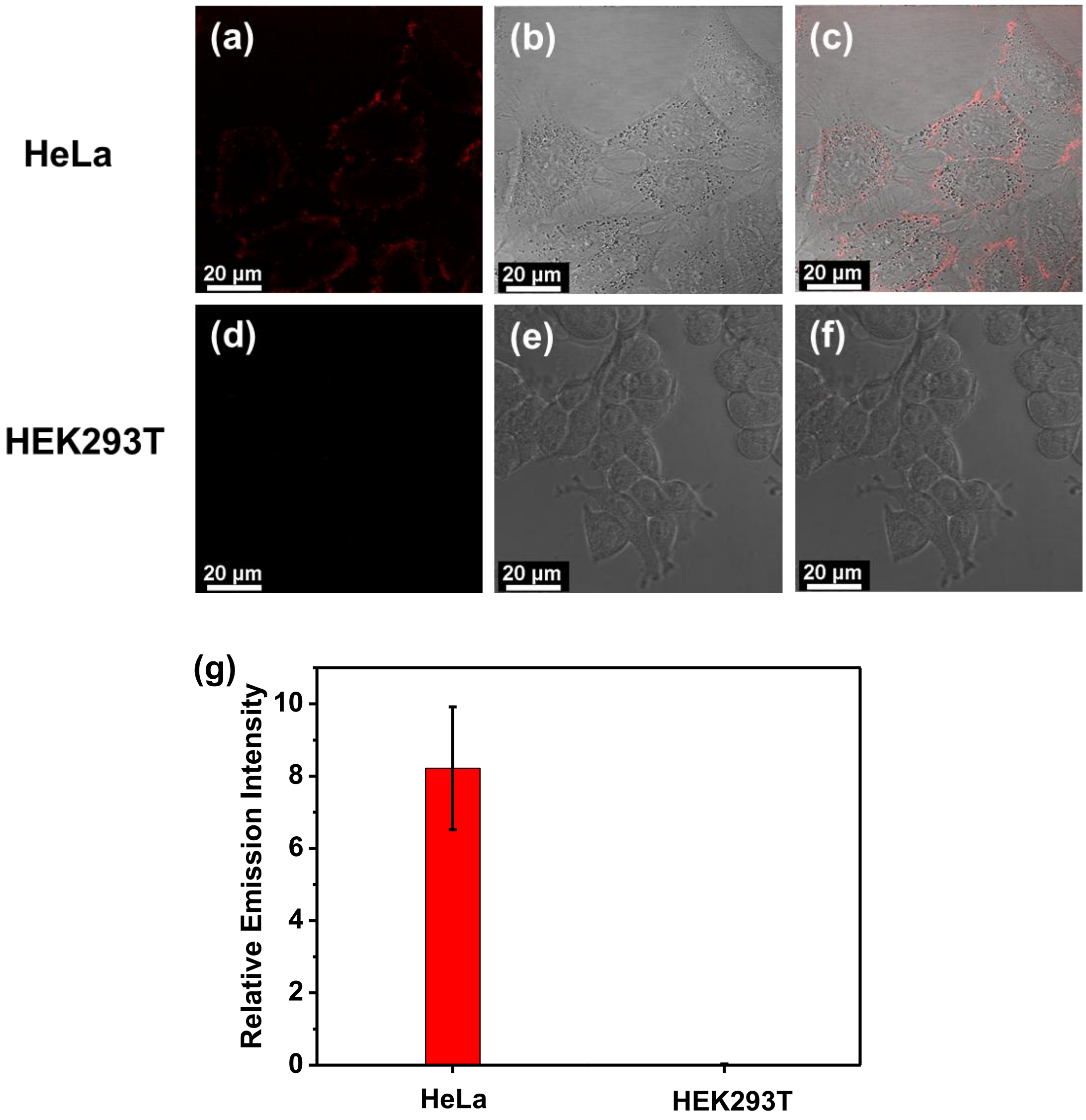

Figure S55. Confocal images of (a–c) live HeLa and (d–f) live HEK293T cells stained with **3** (10 μM) at 37 °C for 30 mins. (a, d) Luminescence, (b, e) bright-field and (c, f) merged confocal images with bright-field and emission collected at 700–800 nm. (g) A bar graph comparing the relative emission intensity of HeLa and HEK293T cells stained with **3**.

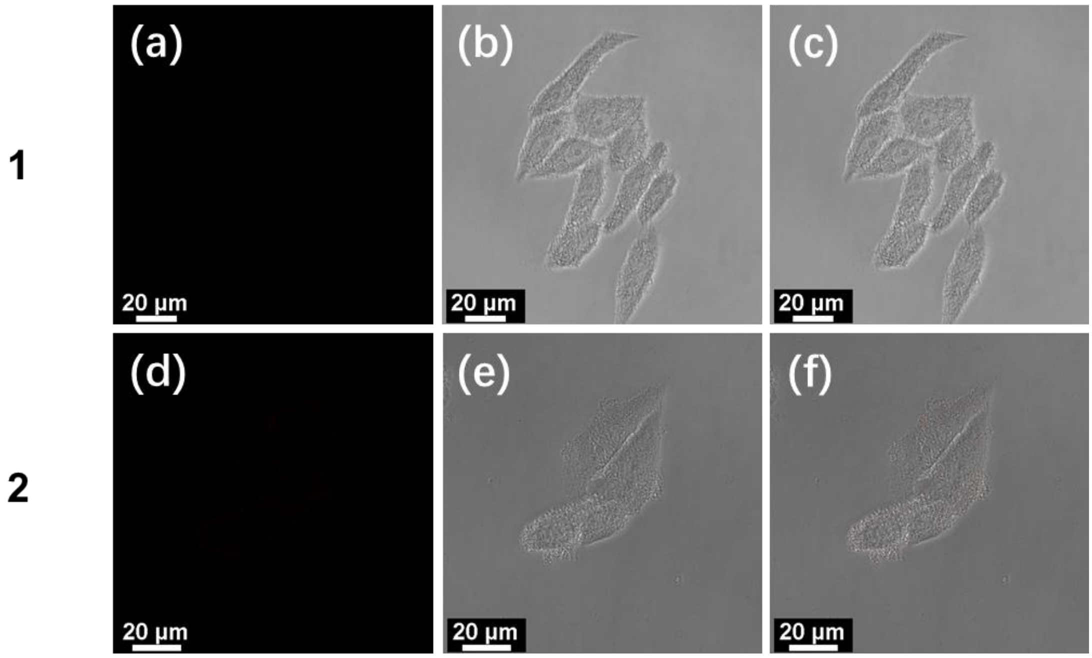

Figure S56. Confocal images of live HepG2 cell stained with (a–c) **1** (10 μM) and (d–f) **2** (10 μM) at 37 ºC for 30 mins. (a, d) Luminescence, (b, e) bright-field and (c, f) merged confocal images with bright-field and emission collected at 700–800 nm.

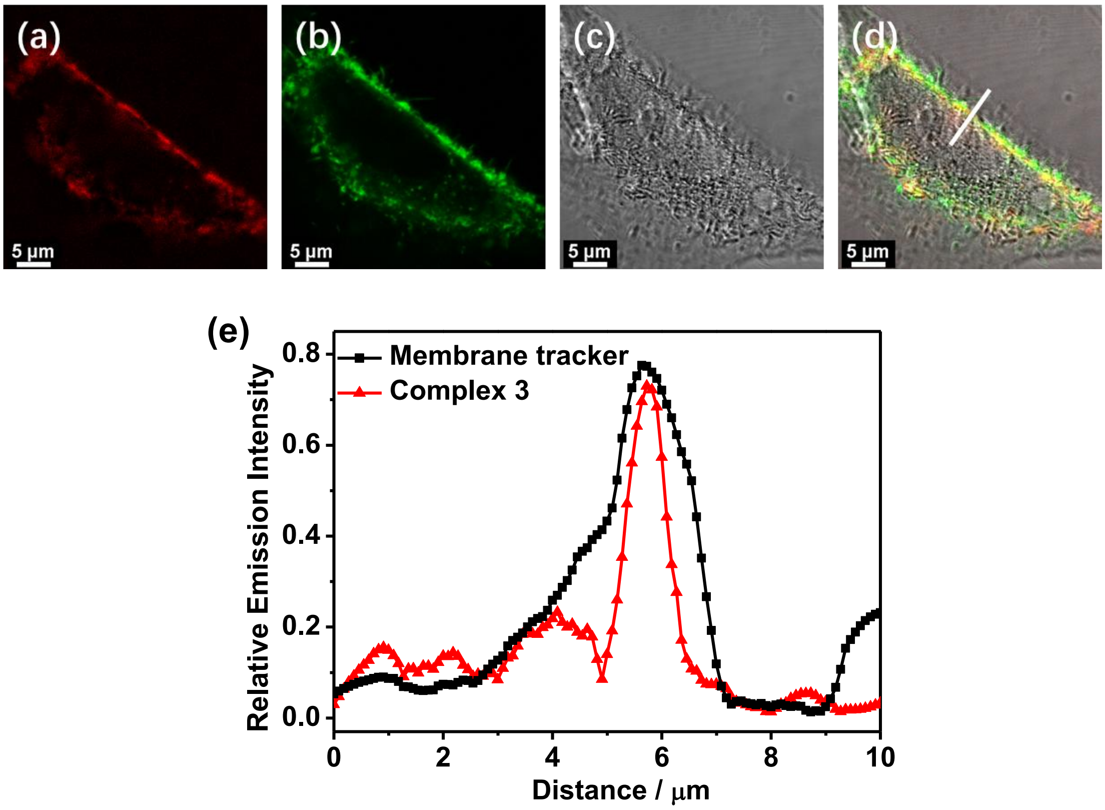

Figure S57. Confocal images of live HepG2 cells stained with **3** (10 μM) at 37 °C for 30 mins, followed by incubation of membrane tracker (5 μg/mL) at 37 °C for 10 min. Luminescence confocal image with emission collected at (a) 700–800 nm, (b) 650–670 nm as well as (c) bright-field and (d) merged confocal images. (e) A line graph showing the overlapping luminescence signals of live HepG2 cells stained with **3** along the membrane tracker with the marked line (-) in (d).

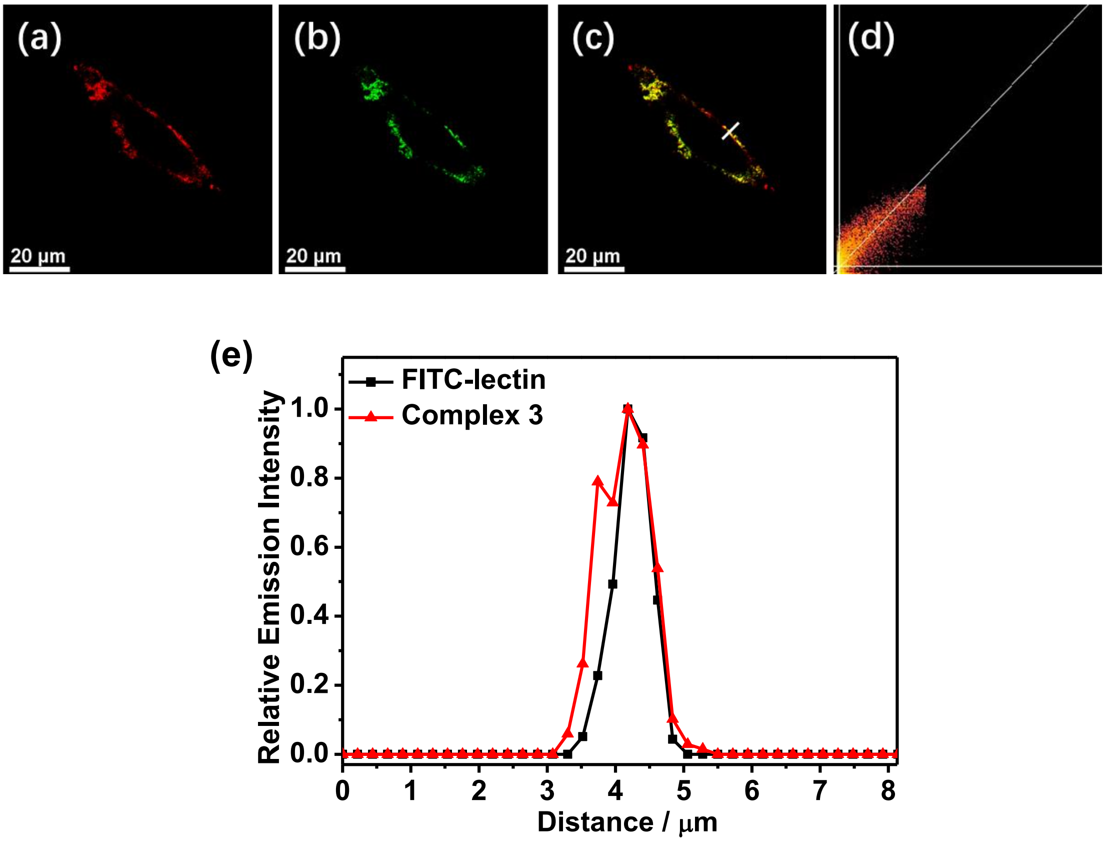

Figure S58. Confocal images of (a–d) live HepG2 cells stained with **3** (10 μM) at 37 °C for 0.5 h, followed by incubation with paraformaldehyde fix solution for 15 mins and FITC-conjugated lectins (20 μg/mL) at 37 °C for 1 h. Luminescence confocal image with emission collected at (a) 700–800 nm, (b) 500–550 nm and (c) merged confocal images. (d) The intensity curve of (a) and (b). (e) A line graph showing the overlapping luminescence signals of live HepG2 cells stained with **3** along FITC-conjugated lectins with the marked line (-) in (c).

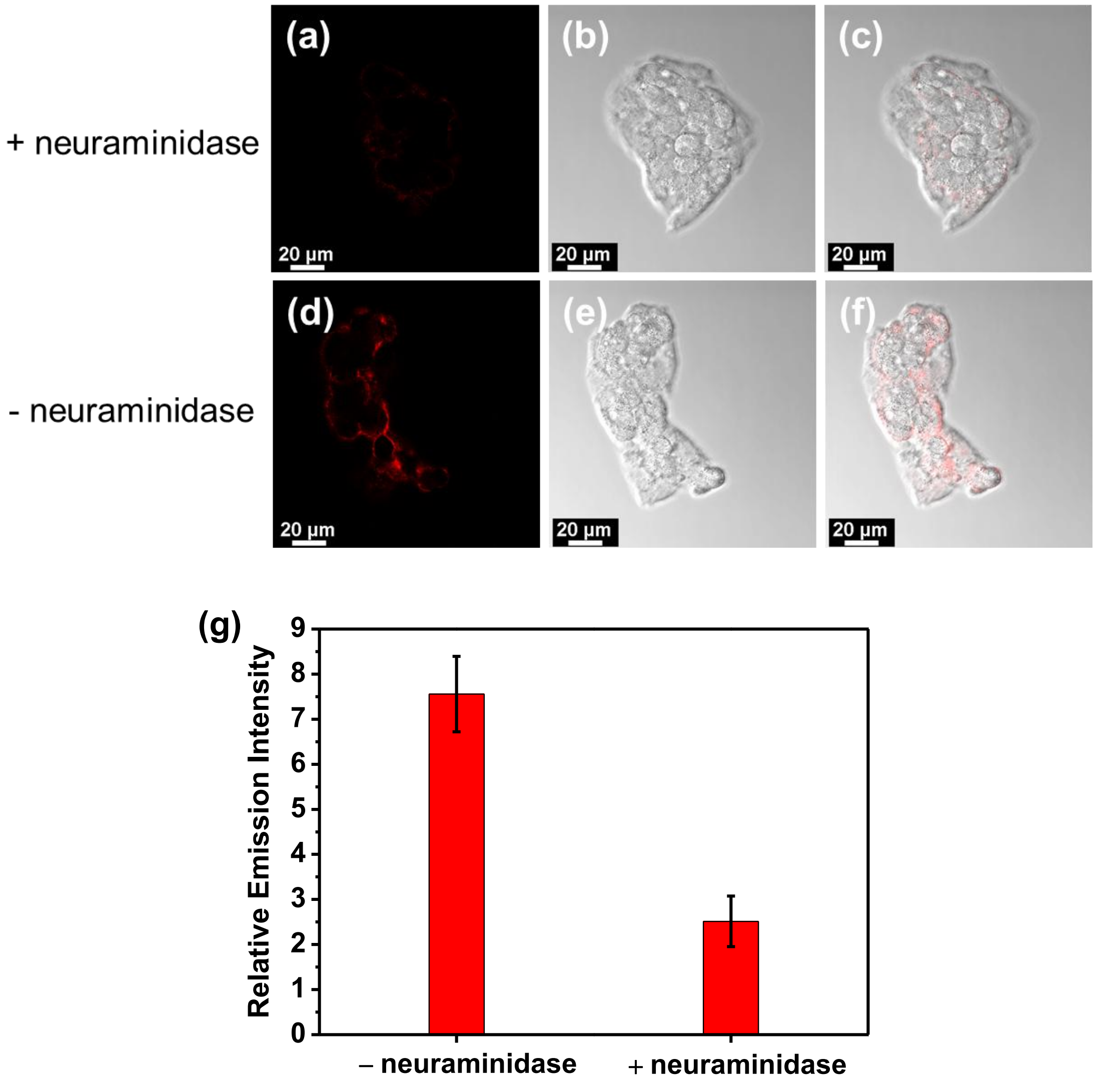

Figure S59. Confocal images of live HepG2 cells stained with **3** and treated (a–c) with the neuraminidase (an enzyme that can remove sialic acids) for 1 h at 37 ºC and (d–e) without the neuraminidase. (a, d) Luminescence, (b, e) bright-field and (c, f) merged confocal images of bright-field and emission collected at 700−800 nm. (g) A bar graph comparing the relative emission intensity of HepG2 cells treated with and without neuraminidase.

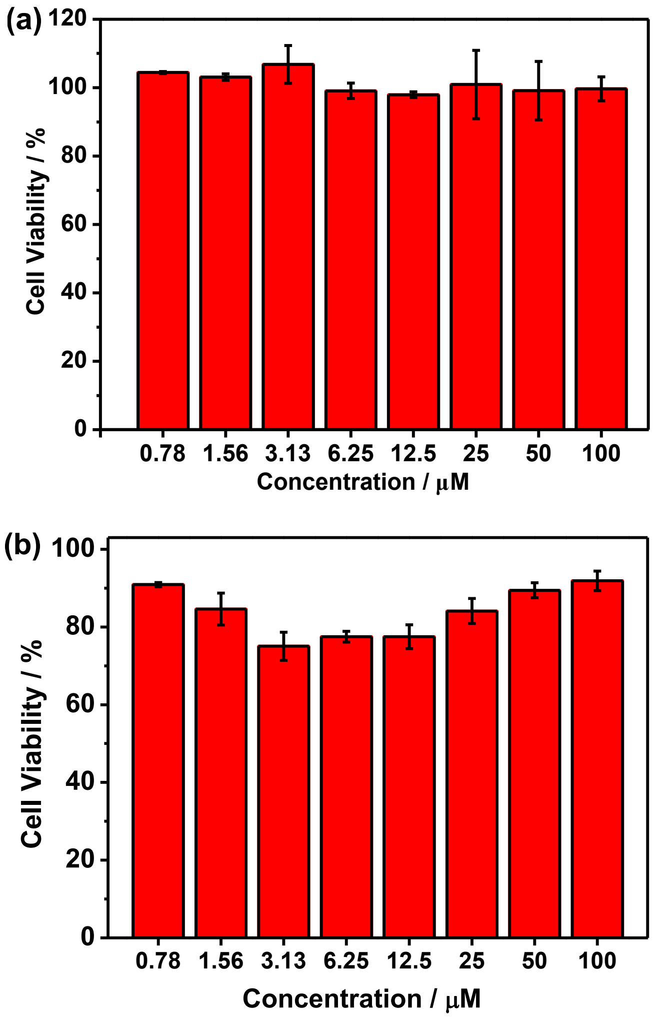

Figure S60. Cell viability of HepG2 cells incubated with different concentrations of **3** (0.78, 1.56, 3.13, 6.25, 12.5, 25, 50, 100 µM) after incubation at 37 °C for (a) 24 h and (b) 48 h.

References

S1. (a) Chung, C. Y.-S.; Li, S. P.-Y.; Lo, K. K.-W.; Yam, V. W.-W. Synthesis and Electrochemical, Photophysical, and Self-Assembly Studies on Water-Soluble pH-Responsive Alkynylplatinum(II) Terpyridine Complexes. *Inorg. Chem.* **2016**, *55*, 4650–4663; (b) Bailey, J. A.; Hill, M. G.; Marsh, R. E.; Miskowski, V. M.; Schaefer, W. P.; Gray, H. B. Electronic Spectroscopy of Chloro(terpyridine)platinum(II). *Inorg. Chem.* **1995**, *34*, 4591–4599.

S2. Reijenga, J.; Van Hoof, A.; Van Loon, A.; Teunissen, B. Development of Methods for the Determination of pK_a Values. *Anal. Chem. Insights* **2013**, *8*, 53–71.

S3. Gaussian 16, Revision C.01, Frisch, M. J.; Trucks, G. W.; Schlegel, H. B.; Scuseria, G. E.; Robb, M. A.; Cheeseman, J. R.; Scalmani, G.; Barone, V.; Petersson, G. A.; Nakatsuji, H.; Li, X.; Caricato, M.; Marenich, A. V.; Bloino, J.; Janesko, B. G.; Gomperts, R.; Mennucci, B.; H. Hratchian, P.; Ortiz, J. V.; Izmaylov, A. F.; Sonnenberg, J. L.; Williams-Young, D.; Ding, F.; Lipparini, F.; Egidi, F.; Goings, J.; Peng, B.; Petrone, A.; Henderson, T.; Ranasinghe, D.; Zakrzewski, V. G.; Gao, J.; Rega, N.; Zheng, G.; Liang, W.; Hada, M.; Ehara, M.; Toyota, K.; Fukuda, R.; Hasegawa, J.; Ishida, M.; Nakajima, T.; Honda, Y.; Kitao, O.; Nakai, H.; Vreven, T.; Throssell, K.; Montgomery, J. A.; Peralta, Jr., J. E.; Ogliaro, F.; Bearpark, M. J.; Heyd, J. J.; Brothers, E. N.; Kudin, K. N.; Staroverov, V. N.; Keith, T. A.; Kobayashi, R.; Normand, J.; Raghavachari, K.; Rendell, A. P.; Burant, J. C.; Iyengar, S. S.; Tomasi, J.; Cossi, M.; Millam, J. M.; Klene, M.; Adamo, C.; Cammi, R.; Ochterski, J. W.; Martin, R. L.; Morokuma, K.; Farkas, O.; Foresman, J. B.; Fox, D. J. Gaussian, Inc., Wallingford CT, 2019.

S4. Perdew, J. P.; Ernzerhof, M.; Burke, K. Rationale for Mixing Exact Exchange with Density Functional Approximations. *J. Chem. Phys.* **1996**, *105*, 9982–9985.

S5. Adamo, C.; Barone, V. Toward Rliable Density Functional Methods Without Adjustable Parameters: The PBE0 Model. *J. Chem. Phys.* **1999**, *110*, 6158–6170.

S6. Marenich, A. V.; Cramer, C. J.; Truhlar, D. G. Universal Solvation Model Based on Solute Electron Density and on a Continuum Model of the Solvent Defined by the Bulk Dielectric Constant and Atomic Surface Tensions. *J. Phys. Chem. B* **2009**, *113*, 6378–6396.

S7. Andrae, D.; Häußermann, U.; Dolg, M.; Stoll, H.; Preuß, H. Energy-Adjustedab Initio Pseudopotentials for the Second and Third Row Transition Elements. *Theor. Chim. Acta* **1990**, *77*, 123–141.

S8. Dolg, M.; Pyykkö, P.; Runeberg, N. Calculated Structure and Optical Properties of $Tl_2Pt(CN)_4$. *Inorg. Chem.* **1996**, *35*, 7450–7451.

S9. Hehre, W. J.; Ditchfield, R.; Pople, J. A. Self-Consistent Molecular Orbital Methods. XII. Further Extensions of Gaussian—Type Basis Sets for Use in Molecular Orbital Studies of Organic Molecules. *J. Chem. Phys.* **1972**, *56*, 2257–2261.

S10. Hariharan, P. C.; Pople, J. A. The Influence of Polarization Functions on Molecular Orbital Hydrogenation Energies. *Theor. Chim. Acta* **1973**, *28*, 213–222.

S11. Dill, J. D.; Pople, J. A. Self-Consistent Molecular Orbital Methods. XV. Extended

Gaussian-Type Basis Sets for Lithium, Beryllium, and Boron. *J. Chem. Phys.* **1975**, *62*, 2921−2923.

S12. Francl, M. M.; Pietro, W. J.; Hehre, W. J.; Binkley, J. S.; Gordon, M. S.; DeFrees, D. J.; Pople, J. A. Self-Consistent Molecular Orbital Methods. XXIII. A Polarization-Type Basis Set for Second-Row Elements. *J. Chem. Phys.* **1982**, *77*, 3654−3665.

S13. Boto, R. A.; Peccati, F.; Laplaza, R.; Quan, C.; Carbone, A.; Piquemal, J.-P.; Maday, Y.; Contreras-Garcia, J. NCIPLOT4: Fast, Robust, and Quantitative Analysis of Noncovalent Interactions. *J. Chem. Theory Comput.* **2020**, *16*, 4150–4158.

S14. Johnson, E. R.; Keinan, S.; Mori-Sanchez, P.; Contreras-Garcia, J.; Cohen, A. J.; Yang, W. Revealing Noncovalent Interactions. *J. Am. Chem. Soc.* **2010**, *132*, 6498−6506.

S15. Contreras-Garcia, J.; Johnson, E. R.; Keinan, S.; Chaudret, R.; Piquemal, J-P.; Beratan, D. N.; Yang, W. NCIPLOT: A Program for Plotting Noncovalent Interaction Regions. *J. Chem. Theory Comput.* **2011**, *7*, 625−632.

S16. Humphrey, W.; Dalke, A.; Schulten, K. VMD: Visual Molecular Dynamics. *J. Mol. Graphics* **1996**, *14*, 33−38.

S17. Dunning, T. H. Gaussian Basis Sets for Use in Correlated Molecular Calculations. I. The Atoms Boron through Neon and Hydrogen. *J. Chem. Phys.* **1989**, *90*, 1007−1023.

S18. Figgen, D.; Peterson, K. A.; Dolg, M.; Stoll, H. Energy-Consistent Pseudopotentials and Correlation Consistent Basis Sets for the 5d Elements Hf–Pt. *J. Chem. Phys.* **2009**, *130*, 164108.

S19. Halkie, A.; Helgaker, T.; Jørgensen, P.; Klopper, W.; Olsen, J. Basis-Set Convergence of the Energy in Molecular Hartree–Fock Calculations. *Chem. Phys. Lett.* **1999**, *302*, 437−446.
